# *Candida albicans* disorder is associated with gastric carcinogenesis

**DOI:** 10.7150/thno.55209

**Published:** 2021-03-05

**Authors:** Mengya Zhong, Yubo Xiong, Jiabao Zhao, Zhi Gao, Jingsong Ma, Zhengxin Wu, Yongxi Song, Xuehui Hong

**Affiliations:** 1 Department of Gastrointestinal Surgery, Zhongshan Hospital of Xiamen University, Xiamen, Fujian, China.; 2 School of Medicine, Xiamen University, Xiamen, Fujian, China.; 3 National Center for International Research of Biological Targeting Diagnosis and Therapy, Guangxi Key laboratory of Biological Targeting Diagnosis and Therapy Research, Guangxi Medical University, Nanning, Guangxi, China.; 4 School of Medicine, Guangxi University, Nanning, Guangxi, China.; 5 Department of Surgical Oncology and General Surgery, Key Laboratory of Precision Diagnosis and Treatment of Gastrointestinal Tumors, Ministry of Education, The First Affiliated Hospital of China Medical University, Shenyang, Liaoning, China.

**Keywords:** Gastric cancer, fungal imbalance, *Candida albicans*, mycobiome, biomarker

## Abstract

**Background:** Bacterial infection is associated with gastric carcinogenesis. However, the relationship between nonbacterial components and gastric cancer (GC) has not been fully explored. We aimed to characterize the fungal microbiome in GC.

**Methods:** We performed ITS rDNA gene analysis in cancer lesions and adjacent noncancerous tissues of 45 GC cases from Shenyang, China. Obtaining the OTUs and combining effective grouping, we carried out species identifications, alpha and beta diversity analyses, and FUNGuild functional annotation. Moreover, differences were compared and tested between groups to better investigate the composition and ecology of fungi associated with GC and find fungal indicators.

**Results:** We observed significant gastric fungal imbalance in GC. Principal component analysis revealed separate clusters for the GC and control groups, and Venn diagram analysis indicated that the GC group showed a lower OTU abundance than the control. At the genus level, the abundances of 15 fungal biomarkers distinguished the GC group from the control, of which *Candida* (*p* = 0.000246) and *Alternaria* (*p* = 0.00341) were enriched in GC, while *Saitozyma* (*p* = 0.002324) and *Thermomyces* (*p* = 0.009158) were decreased. Combining the results of Welch's t test and Wilcoxon rank sum test, *Candida albicans* (*C. albicans*) was significantly elevated in GC. The species richness Krona pie chart further revealed that *C. albicans* occupied 22% and classified GC from the control with an area under the receiver operating curve (AUC) of 0.743. Random forest analysis also confirmed that *C. albicans* could serve as a biomarker with a certain degree of accuracy. Moreover, compared with that of the control, the alpha diversity index was significantly reduced in the GC group. The Jaccard distance index and the Bray abundance index of the PCoA clarified separate clusters between the GC and control groups at the species level (*p* = 0.00051). Adonis (PERMANOVA) analysis and ANOVA showed that there were significant differences in fungal structure among groups (*p* = 0.001). Finally, FUNGuild functional classification predicted that saprotrophs were the most abundant taxa in the GC group.

**Conclusions:** This study revealed GC-associated mycobiome imbalance characterized by an altered fungal composition and ecology and demonstrated that *C. albicans* can be a fungal biomarker for GC. With the significant increase of *C. albicans* in GC, the abundance of* Fusicolla acetilerea, Arcopilus aureus, Fusicolla aquaeductuum* were increased, while *Candida glabrata, Aspergillus montevidensis*, *Saitozyma podzolica* and *Penicillium arenicola* were obviously decreased. In addition, *C. albicans* may mediate GC by reducing the diversity and richness of fungi in the stomach, contributing to the pathogenesis of GC.

## Introduction

Gastric cancer (GC) is the fourth most common malignancy and one of the main causes of cancer-related deaths worldwide 1. The majority of GC cases are the intestinal type of noncardia gastric cancer, which undergoes a predictable histological progression from atrophic gastritis (AG) to intestinal metaplasia (IM) and eventually to GC 2. Initially, *Helicobacter pylori (H. pylori)* infection causes inflammation of the gastric mucosa and destruction of related hydrochloric acid secreting glands, leading to AG 3. AG is a chronic inflamed, hypochloremic state, which may cause GC. Although it is known that *H. pylori* infection contributes to this cascade, only approximately 1-3% of infected individuals subsequently develop GC 4, 5. Some host-related factors mentioned in the current research, including age, smoking status, genetic susceptibility and environmental factors, such as consumption of a high-salt diet and smoked foods containing nitrates, as well as microbial infections, have been shown to contribute to gastric carcinogenesis 6. However, the relationship between gastric microbial components (such as fungi) other than *H. pylori* and GC has not been fully explored.

Over the past decade, due to the difficulty in culturing the commensal microorganisms that reside in the stomach, compared with intestinal microbiome, gastric microbiome studies are few, with only recent increases in studied on this topic 7. In recent years, combined with advances in PCR techniques and metagenomics, the robust microbiome of the stomach has attracted extensive attention 8. Most of the research efforts on the microbiome have focused on characterizing bacteria in healthy and diseased states, while the relatively low abundance of nonbacterial components has been neglected because of various technical challenges ranging from sample preparation to inadequate reference databases. Studies have provided evidence that bacteria, mainly the phyla *Proteobacteria, Firmicutes, Actinobacteria* and* Fusobacteria* 9, 10, can be regularly detected in gastric biopsies with gastric microbial imbalance associated with GC. Although *H. pylori* is still the main risk factor for histological changes, the chance of evolving GC after infection is not high, indicating that the presence of other components plays a key role in the development of GC.

With the advancement of high-throughput sequencing technology, sequencing methods provide access to the gastric mycobiome. Genomic equivalence estimates that the fungal composition of the mammalian microbiota comprises less than 1% of all commensal microbial species, but fungi are significantly larger than bacteria in cell size and possess specialized metabolic gene clusters in response to specific ecological needs. Emerging research has revealed that fungi play a stable role in the development and maintenance of the host immune system and can be altered in various diseases 11, 12. The latest Nature journal reports that fungi, like bacteria, can also be transferred from the intestine to the pancreas, and related changes in the fungal microbiome promote pancreatic oncogenesis 13. With the discovery of the role for gut microbiota dysbiosis in colorectal, oral, and pancreatic carcinogenesis, it is necessary to conduct further studies regarding the role of mycobiome as a potential prognostic tool for early diagnosis of cancer 14. Additionally, growing attention towards the characterization of mycobiome may contribute to improving the efficiency of therapeutic methods used to modulate the composition and activity of intestinal microbiota 14. Thus, the dynamic exploration of the changes in the composition of gastric fungi in the progression from health to GC not only provides direction for future high-throughput fungal sequencing research on tumors but is also essential for further investigating the mechanisms of gastric carcinogenesis other than *H. pylori*.

In this study, we characterized fungal compositional and ecological changes by analyzing metagenomic sequences in cancer lesions and adjacent noncancerous tissues of 45 patients with GC. *C. albicans* was also discovered as a fungal indicator for GC. For the first time, we used ITS sequencing to demonstrate the importance of fungi in the pathogenesis of GC, providing a theoretical scientific basis for the development of potential prevention and treatment strategies.

## Results

### Gastric fungal imbalance is associated with GC

**Figure 1:**
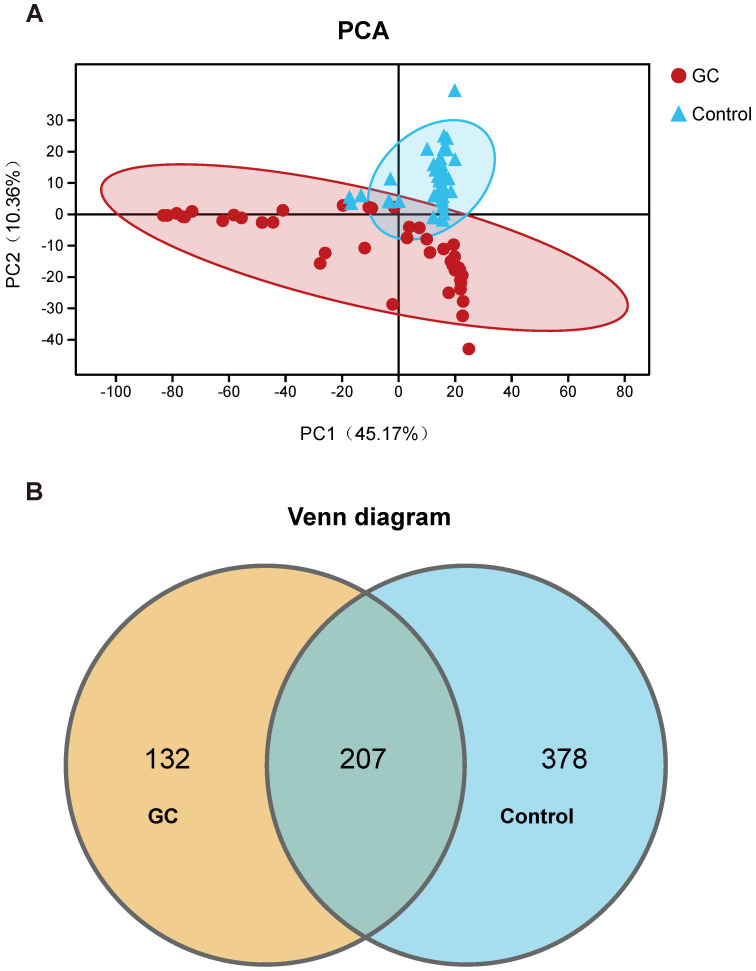
** Classification and distribution of fungi in the stomachs of gastric cancer (GC) patients.** (A) Through the principal component analysis (PCA) dynamic display, GC (n=45) and control (n=45) samples showed clustering distributions. PC1 and PC2 represent the first two main components, and they reflect the contribution to the sample difference, expressed as a percentage. (B) Based on the OTU abundance, Venn diagram analysis was performed. Unique OTUs between the GC (orange) and control (blue) groups was found as well as common OTUs (lightcyan) between the two groups.

We evaluated 90 samples from 45 pairs of patients and divided them into a GC group and a control group (adjacent noncancerous tissue) for comparison. We also analyzed the clinical characteristics closely related to GC and found no significant differences. The detailed characteristics of the patients are shown in Table S1. We first assessed and compared the fungal composition in the specimens. The PCA showed that the GC and control groups aggregated separately, revealing that the gastric mucosal fungal community discriminated GC and the control into two significantly distinct groups. The GC group exhibited more unique fungal profiles than the control group (Figure 1A, Table S2). To clarify the OTU crossover between different groups, we used a Venn diagram to indicate the differences among the groups according to OTU abundance. We found that both groups shared a total OTU abundance of 207. Simultaneously, the GC group showed a lower OTU abundance than the control group (Figure 1B). Meanwhile, we also obtained 10 healthy samples. We didn't find significant difference between healthy individuals and the adjacent noncancerous tissues (Figure S1). Besides, when we added the 10 healthy samples to the 45 adjacent non-cancerous tissues, the results of 55 non-cancerous specimens compared to 45 GC samples are basically the same as the comparing results between 45 pairs (Figure S2). Taking into account the rigorous comparision of the experiment and the statistical difference, we finally show the results of 45 pairs of cancer and adjacent noncancerous samples for the further analysis. Based on these OTU clustering results, it is suggested that alterations in stomach fungal composition may be associated with gastric carcinogenesis.

### Taxonomic coverage and alterations of fungi in GC

**Figure 2:**
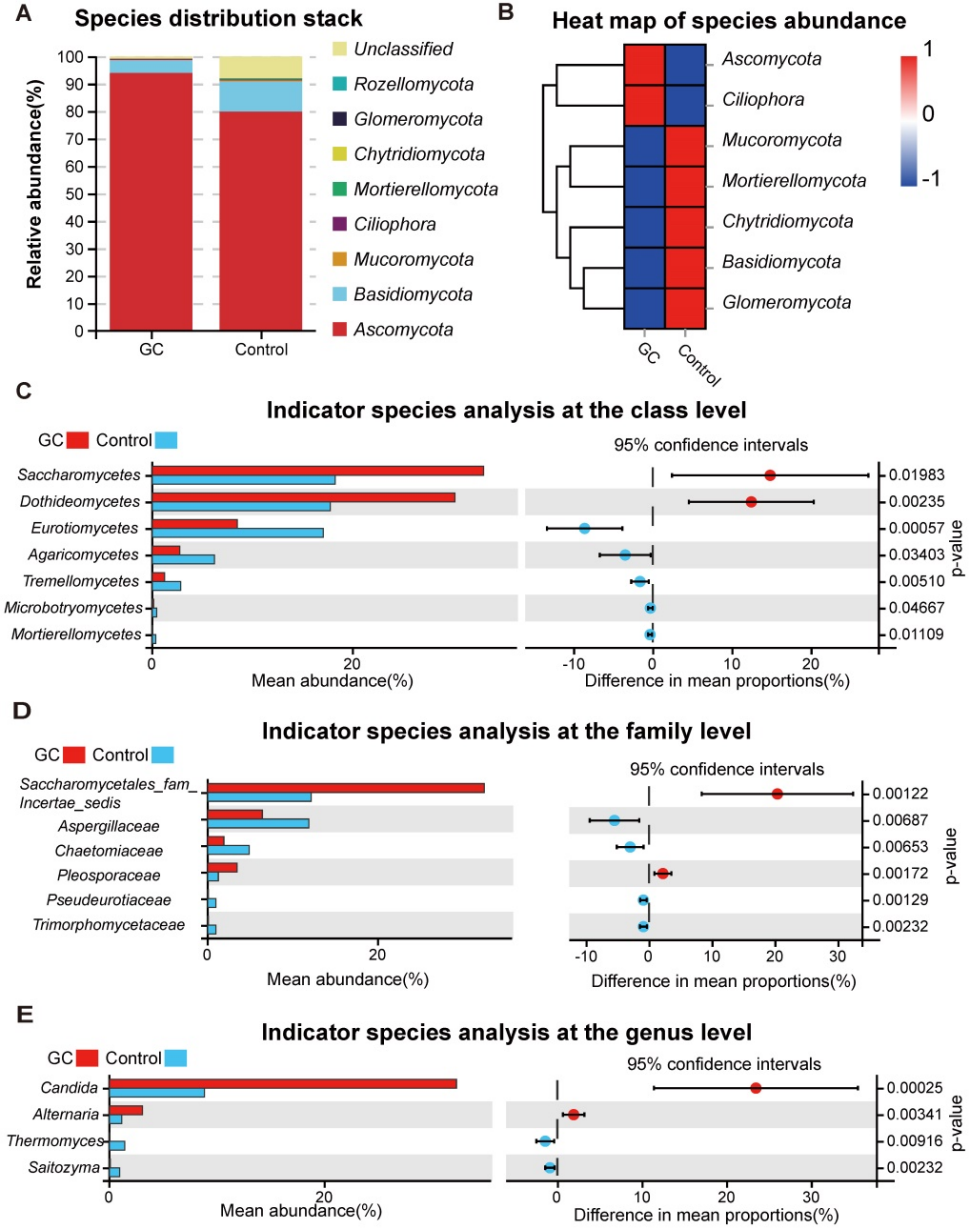
** Changes in the fungal composition in the stomachs of gastric cancer (GC) patients.** (A) Relative abundance of dominant gastric fungal phyla in the GC and control groups. The dominant phyla were Ascomycota and Basidiomycota in both groups. (B) The corresponding heatmap also shows changes in the fungal phyla in the GC and control groups. Differences in fungal composition and abundance between GC (n=45) and the control (n=45) were detected using Welch's t test. The variation in the relative abundance of species represented in different groups was demonstrated graphically. Differences in OTUs appear in the left rows, and the corresponding P values are shown in the right rows. (C) Differentially abundant fungal classes between the GC and control groups. OTUs and taxa differences are shown with p-values less than 0.05. Differentially abundant fungal families (D) or genera (E) between the GC and control groups. OTUs and taxa differences are shown with p-values less than 0.01.

For the distribution of fungal taxa, in both the GC and control groups, the phylum *Ascomycota* was the dominant mycoflora, and *Basidiomycota* was considered to be the second most abundant phylum (Figure 2A). The corresponding species abundance heat map is shown in Figure 2B. We further analyzed the differences at the lower taxonomic level of class, finding a significant depletion of *Eurotiomycetes*, *Agaricomycetes*, *Tremellomycetes*, *Microbotryomycetes* and *Mortierellomycetes* and enrichment of *Saccharomycetes* and *Dothideomycetes* in the GC group compared with the control group (Figure 2C). At the family level, we found 17 fungi with significant differences (Table S3), so we only showed data with a P value less than 0.01. *Pseudeurotiaceae, Trimorphomycetaceae, Chaetomiaceae* and* Aspergillaceae* were significantly decreased in the GC group, while *Saccharomycetales_fam_Incertae_sedis* and *Pleosporaceae* were increased, compared to the control (Figure 2D). Furthermore, at the genus level, there were 15 different fungi between the two groups (Table S4); 2 fungal genera were enriched in the GC group, including *Candida* (*p* = 0.000246) and *Alternaria* (*p* = 0.00341), while *Saitozyma* (*p* = 0.002324) and *Thermomyces* (*p* = 0.009158) were decreased, compared to the control (Figure 2E).

### *Candida albicans* as a fungal indicator species for GC

To better identify fungal taxa with value as potential GC indicators, we evaluated fungal alterations at the species level. We initially used Welch's t test and found that there were 13 species with significant differences in the mean abundance when comparing the two groups (Table S5). Then, the Wilcoxon rank sum test was applied to determine whether the median species abundance was statistically significant, and we confirmed that 59 species had significant differences between the two groups (Table S6). The species with higher contents and greater than two-fold changes in abundance were selected for the next analysis.

**Figure 3:**
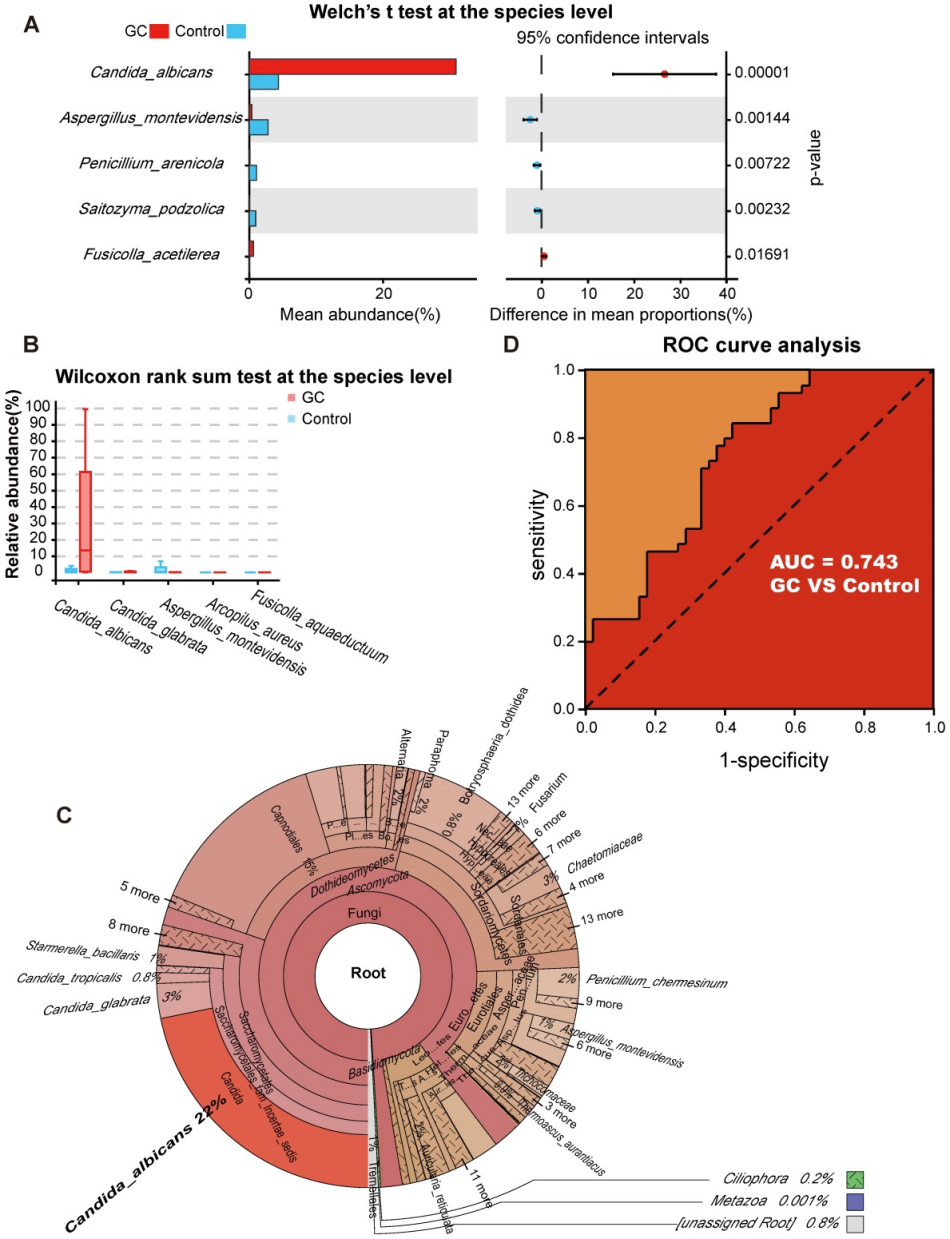
***Candida albicans* as an indicator fungus for GC.** Differences in fungal species abundance between the GC (n=45) and control (n=45) groups were detected using Welch's t test (A) or Wilcoxon rank sum test (B), and *Candida albicans* was significantly elevated in the GC group (p<0.0001). (C) Species annotation was performed based on the sequence information of the OTUs, a Krona pie chart was established at the species level, and the absolute abundance of *C. albicans* accounted for 22%. (D) The markers achieved an area under the receiver operating characteristic curve (AUC) of 0.743 for the classification of the GC group from the control group.

**Figure 4:**
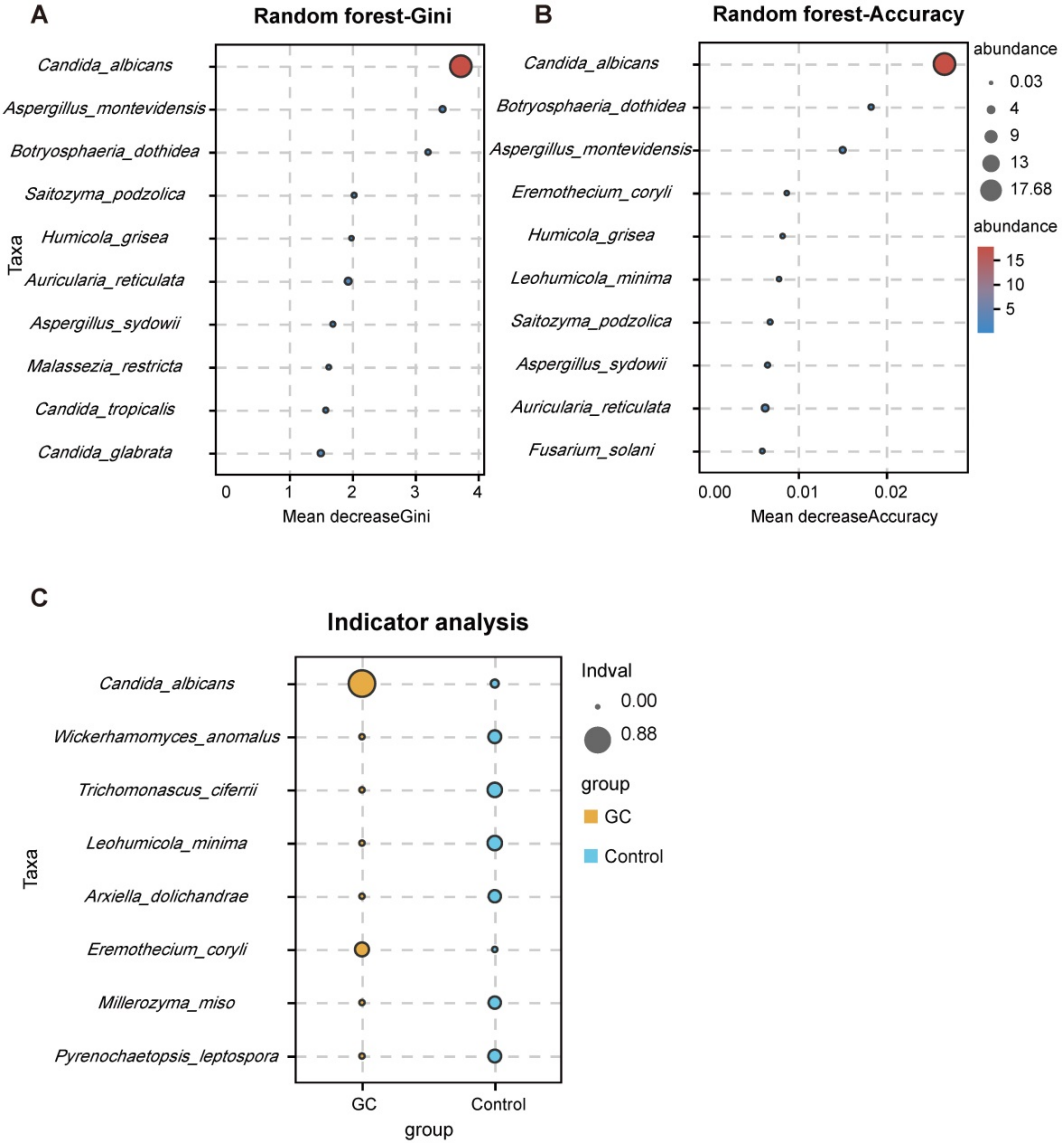
***Candida albicans* has a strong indication ability.** Using the random forest algorithm to calculate the contribution of *C. albicans* to the grouping difference at the species level, it is found that the Gini index (A) and average accuracy (B) values were both largest for *C. albicans*. (C) The indicator analysis considers the frequency and abundance of *C. albicans* between groups.

With the Welch's t test, *C. albicans* (*p* = 0.000015) and *Fusicolla acetilerea* (*p* = 0.01691) were increased, while *Aspergillus montevidensis* (*p* = 0.001437), *Saitozyma podzolica* (*p* = 0.002324) and *Penicillium arenicola* (*p* = 0.00722) were obviously decreased in the GC group (Figure 3A). With the Wilcoxon rank sum test, the abundance of *C. albicans* (*p* = 0.000072), *Arcopilus aureus* (*p* = 0.040759) and *Fusicolla aquaeductuum* (*p* = 0.026626) was higher in the GC group, while *Candida glabrata* (*p* = 0.014443) and *Aspergillus montevidensis* (*p* = 0.000586) were less abundant, compared to the control (Figure 3B). These results demonstrated that *C. albicans* was significantly elevated in the GC group (*p* < 0.0001). Next, we dynamically displayed the composition of species at different classification levels through the species composition pie chart and found that the abundance of *C. albicans* at the species level accounted for 22% (Figure 3C, Table S7). We evaluated the accuracy based on the ROC curve and observed an AUC value of 0.743 (Figure 3D). Random forest analysis was used to screen potential indicator species, and the values of the Gini index (Figure 4A) and the mean decrease in accuracy (Figure 4B) were the largest for *C. albicans*. Combined with the indicator analysis, we comprehensively considered the strong indicator ability of *C. albicans* among the groups (Figure 4C). These results all indicated that *C. albicans* had an obvious effect in distinguishing GC and non-GC tissues and can be used as a biomarker with a certain degree of accuracy.

### Altered fungal microbiota diversity in GC

**Figure 5:**
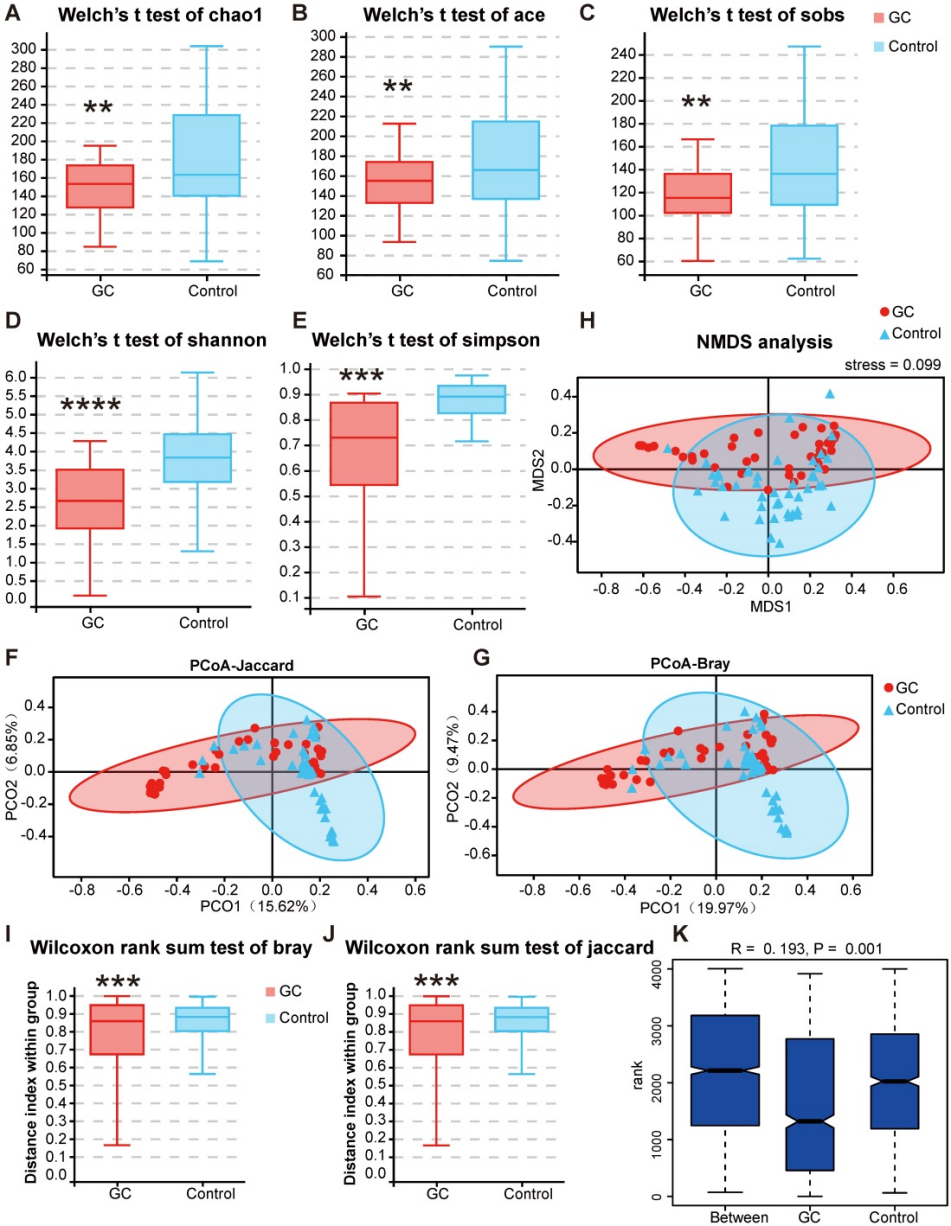
** Changes in fungal microbiome diversity in GC.** Hypothesis tests of the alpha diversity index through Welch's t test, Chao1 (A), ACE (B), Sobs (C), Shannon (D) and Simpson (E) diversity indexes between the GC (n=45) and control (n=45) groups confirmed that there were significant differences in species diversity between groups. Principal coordinate analysis (PCoA) of Jaccard distances (F) or Bray-Curtis distances (G) showed the stratification of GC (n=45) from control (n=45) samples by their fungal compositional profiles. (H) Nonmetric multidimensional scaling (NMDS) analysis of the fungal compositional profiles stratified GC (n=45) from control (n=45) samples. A stress value less than 0.1 indicates that the model grouping is reliable. At the genus level, the Wilcoxon rank sum test was used to judge the significant difference between the Bray-Curtis distance (I) and Jaccard distance (J), and the degree of difference in fungal microbiome structure within the groups was compared. (K) Based on the distance index ranking, ANOSIM (analysis of similarities) confirmed that the distance between groups was significantly greater than the distance within groups, indicating that the microbiome structure of different groups was significantly different. **P<0.01, ***P<0.001, ****P<0.0001.

Next, we conducted a diversity analysis to further understand the species richness and microbiome structure among the groups. Alpha diversity indexes (Chao1, ACE, Sobs, Shannon, Simpson and Good's Coverage) were significantly reduced in the GC group compared with those of the control (Figure S3, Table S8). Briefly, we measured fungal alpha diversities and determined whether, through a t test (Figure 5A-E) or rank sum test, five indexes, namely, the Chao1, ACE, Sobs, Shannon and Simpson indexes, were significantly different between the GC and control groups (*p <* 0.05) (Table 1).

We used PCoA to analyze two classic beta diversity indexes, the Jaccard distance index (Figure 5F) and the Bray abundance index (Figure 5G), and confirmed separate clusters for the GC and control groups at the species level. To overcome the shortcomings of linear models (PCA, PCoA) and better reflect the nonlinear structure, we evaluated the accuracy of the model through NMDS stress values. We ensured the reliability of the model, confirming that the stress values of the Jaccard and Bray indexes were less than 0.1 (Figure 5H). The significant difference of the two indexes between groups was shown by the Wilcoxon rank sum test at the genus level (*p* = 0.00051, Figure 5I-J). We then evaluated and verified the fungal composition in our groups. Both Adonis (PERMANOVA) analysis (*p* = 0.001) and the ANOSIM test (Figure 5K) revealed that there were significant differences in fungal structure between the GC and control groups. Combining the two diversity index results, our analysis suggested that with gastric carcinogenesis, the richness of the related fungal composition decreases, and the structure of the fungal community is quite different.

### Ecological guilds of sampled taxa

**Figure 6:**
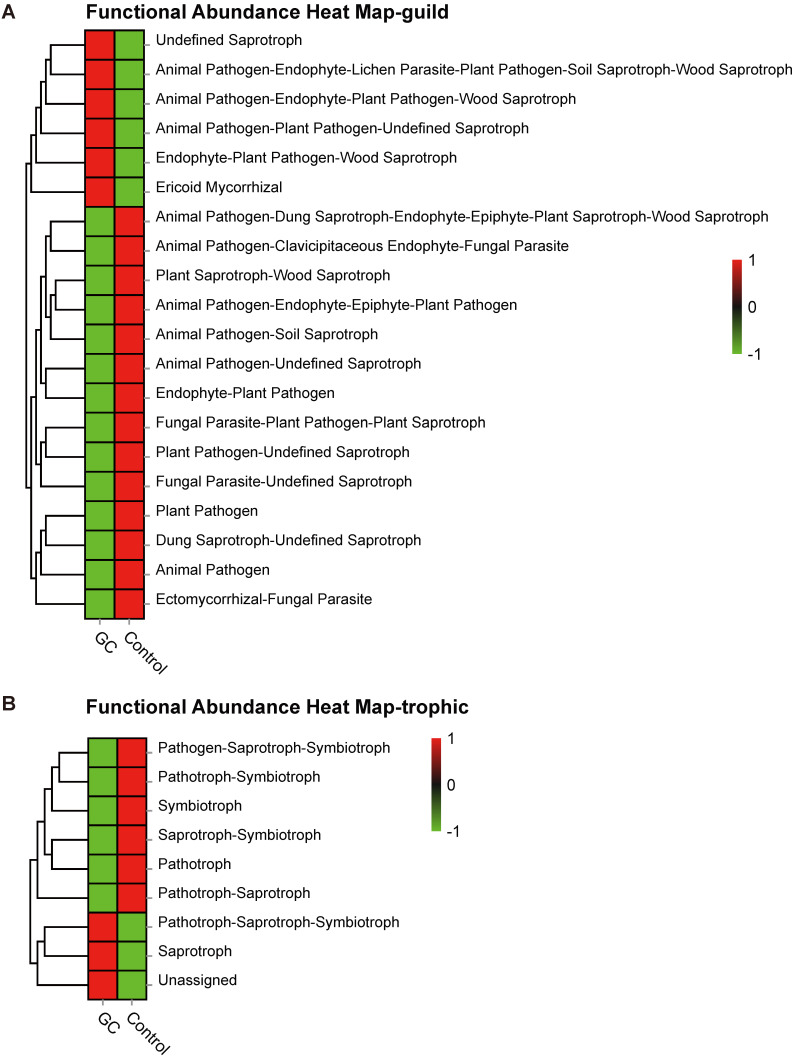
** Saprotrophs are the most common functional category associated with GC.** Based on the OTU abundance, fungal functional annotation was carried out using FUNGuild. Using functional groups (guilds), fungi were divided into categories based on their absorption and utilization of environmental resources. The three major categories and twelve subcategories of fungi distinguished the GC (n=45) and control groups (n=45) at the guild (A) and trophic (B) levels.

Based on the OTU abundance, we used FUNGuild to perform functional classification prediction. The fungal taxa were grouped into 83 ecological guilds (Table S9), and top ten categories are displayed here. The most diverse guild was undefined saprotrophs (Figure S4A). In addition, trophic mode divided fungal taxa into 9 types (Table S10), of which the most diverse type was saprotrophs (Figure S4B). In particular, heatmaps were drawn to describe the functional predictions under the two analytical methods, as shown in Figure 6A and Figure 6B, respectively. Thus, our analyses showed a symbiotic ecological relationship in the stomach, which is important for the homeostasis of gastric fungi, while fungal imbalance ultimately indicates the negative effects of gastric carcinogenesis.

## Discussion

Gastric cancer causes one of the major types of digestive tract tumor worldwide 1. After the continuous development of high-throughput sequencing technology, research on the correlation between gastric microbiome (other than *H. pylori*) and GC has gradually emerged. In this study, we described the fungal spectrum associated with GC, which has not been explained to date; the focus was on gastric fungal imbalance associated with GC. Compared with fecal samples, the colonization performance of tissue samples can better demonstrate the dynamic changes in the surrounding environment for gastric carcinogenesis. Therefore, we analyzed the ITS metagenome sequences of cancer lesions and adjacent noncancerous tissues to investigate the composition and ecological alterations of fungi associated with GC and identify fungal indicators. To ensure that the most effective data were clustered into OTUs, we filtered low-quality reads, and assembled and refiltered the data. After obtaining the OTUs, under the condition that the GC and control groups were effectively grouped, we carried out species identifications and alpha and beta diversity analysis, and compared differences between groups. *C. albicans* was identified for the first time as a key fungus that can be used to distinguish between GC and control groups. We also combined FUNGuild functional annotation to study fungal functions from other ecological perspectives. For the first time, we showed the characteristics of the fungal microbiome in the stomach tissues of GC patients, demonstrating imbalance of the fungi in the GC ecosystem and proving that *C. albicans* can be used as a biomarker with a certain degree of accuracy.

We clarified specific fungal composition changes in GC. Overall, the GC group showed a lower OTU abundance. At the phylum level, *Ascomycota* was the most enriched in the GC group compared with the control group, while *Basidiomycota* was less enriched. We further analyzed the differences at lower taxonomic levels and finally, at the species level, confirmed that *C. albicans, Fusicolla acetilerea, Arcopilus aureus* and* Fusicolla aquaeductuum* were excessively colonized in the GC tissue. At present, *C. albicans* is the most researched of these organisms with regard to its role in various diseases. This species normally exists in the body and does not cause damage. However, when the host's defense capacity is weakened, *C. albicans* could cause disease. Therefore, *C. albicans* is recognized as an opportunistic pathogen. Since immunosuppression caused by cancer chemotherapy promotes *C. albicans* infection, the relationship between *C. albicans* and cancer development or progression has been widely reported. For example, for hematological malignancies or solid tumors, up to 35% of patients with underlying disease have candidiasis, and the most common underlying disease among patients with candidiasis is also solid tumor 15. *C. albicans* can produce carcinogenic nitrosamines, which can cause abnormal proliferative changes in oral epithelial cancer 16. The risk of malignant transformation of oral leukoplakia is higher than that of oral lichenoid lesions, and *C. albicans* strains isolated from patients can produce more carcinogenic acetaldehyde in ethanol 17. The role of *C. albicans* in tumor adhesion and metastasis has been associated with TNF-α and IL-18 18-20. Recently, Bertolini et al. confirmed that *C. albicans* induced mucosal bacterial dysbiosis and promoted invasive infection 21. Kazmierczak-Siedlecka et al. found that *C. albicans* is the major gut microbe causing inflammation and consequently contributing to oral cancer development 14.

Notably, we first confirmed the indicative role of *C. albicans* in GC. In our study, compared with the control, the species richness of *C. albicans* occupied 22% in the GC group. Both the Welch's t test and Wilcoxon rank sum test confirmed that *C. albicans* was significantly more abundant in the GC group than the control group. In addition, the ROC curve showed that the AUC value of *C. albicans* was 0.743. Combined with the results of the Gini index and the mean decrease in accuracy, all results indicated that *C. albicans* could be used as a biomarker with a certain degree of accuracy. Routine detection methods for *C. albicans* include blood culture, microscopic examination, and biochemical identification 22, 23, but clinically these tests delay antifungal treatment. To make up for the shortcomings of time-consuming and low sensitivity of conventional examinations, the current molecular biology techniques for detecting fungi have seen a leap in quality and are gradually being applied in clinical practice, including polimerase chain reaction (PCR) 24, real-time PCR 25, mass spectrometry 26, immunoassay 27, Polymerase spiral reaction (PSR) 28 and 18S rDNA high-throughput screening 29, with the advantages of higher sensitivity, faster processing ( < 1 working day) and prospect for a high degree of laboratory automation, these technologies provide an attractive alternative for the identification and quantitation of *C. albicans* rDNA in pure cultures and blood samples. Aykut et al. stated that identifying the species most associated with cancer may guide future attempts to use targeted antifungal drugs to slow tumor growth and avoid side effects and reported *Malassezia* as a pathogenic fungus associated with pancreatic cancer that promotes pancreatic oncogenesis via activation of MBL 13.Our discovery that *C. albicans* may have contributed to the pathogenesis of GC not only lays a scientific foundation for the exploration of innovative therapies for GC but also provides a new idea for treating specific patients by adjusting their intestinal microbial microbiome as an adjuvant therapy or developing immunotherapies for targeted control of fungal infections, which is worthy of further study. Similar with the gut bacteria, we believe that the composition of gastric mycobiome is associated with the ethnicity or region in a certain degree. Thus, more studies from different countries or regions are required to better describe the fungal microbiome of stomach.

By diversity analysis, compared with the control group, the GC group showed a decrease in species richness, diversity and uniformity. The structure of the species microbiome between the groups also showed a significant change. Due to the current lack of fungal genomic data, we integrated published article data and used FUNGuild to predict fungal functions from other ecological perspectives based on OTU abundance. The guild classification revealed that the most diverse guilds were undefined saprotrophs. Simultaneously, the trophic mode implied that the most diverse fungal type was the saprotrophs. Our analysis clarified the importance of fungal homeostasis in the stomach and suggests that fungal imbalance is associated with the occurrence and development of GC.

## Conclusions

In conclusion, compared with most studies focusing on the bacterial spectrum associated with GC, our study described the gastric fungal imbalance in gastric carcinogenesis for the first time and showed that *C. albicans* can be used as a fungal marker for GC. In addition, *C. albicans* may possibly mediate GC by reducing the diversity and richness of fungi in the stomach, contributing to the pathogenesis of GC. We also revealed the importance of homeostasis for gastric fungi. Additional analysis investigating the potential role of *C. albicans* in gastric carcinogenesis is warranted to delineate its use as a noninvasive biomarker for GC diagnosis.

## Materials and Methods

### Sample collection and PCR amplification

A total of 100 samples were obtained from 45 pairs of patients diagnosed with GC as well as 10 healthy individuals (include 7 men and 3 women, with an average age of 64 years) at the First Affiliated Hospital of China Medical University, Shenyang, China. Surgical biopsies were obtained from sites of cancer lesions and adjacent noncancerous tissues in each patient.

All specimens were stored at -80 °C until DNA extraction. In addition, subjects provided informed consent for obtaining study specimens, and the study was approved by the Clinical Research Ethics Committees of the First Affiliated Hospital of China Medical University.

Microbial DNA was extracted using HiPure DNA Kits (Magen, Guangzhou, China) according to the manufacturer's protocols. The internal transcribed spacer (ITS) of the ITS2 region between the 5.8S and 28S genes of the ribosomal DNA gene was amplified by PCR (94 °C for 2 min, 30 cycles at 98 °C for 10 s, 62 °C for 30 s, and 68 °C for 30 s, and a final extension at 68 °C for 5 min) using the fungal-specific primers ITS3_KYO2: GATGAAGAACGYAGYRAA and ITS4: TCCTCCGCTTATTGATATGC 30. PCRs were performed in triplicate in a 50-μL mixture containing 5 μL of 10× KOD buffer, 5 μL of 2 mM dNTPs, 3 μL of 25 mM MgSO4, 1.5 μL of each primer (10 μM), 1 μL of KOD polymerase, and 100 ng of template DNA. The related PCR reagents used in the experiment were from TOYOBO, Japan.

### Metagenomics sequencing

Amplicons were extracted from 2% agarose gels, purified using the AxyPrep DNA Gel Extraction Kit (Axygen Biosciences, Union City, CA, USA) according to the manufacturer's instructions and quantified using the ABI StepOnePlus Real-Time PCR System (Life Technologies, Foster City, USA). The purified amplicons were pooled in equimolar amounts and paired-end sequenced (PE250) on an Illumina platform according to standard protocols. The raw reads were deposited into the NCBI Sequence Read Archive (SRA) database.

### Quality control and read assembly

Raw data containing adapters or low-quality reads affect subsequent assembly and analyses. Thus, to obtain high-quality clean reads, the raw reads were further filtered according to the following rules using FASTP 31 (version 0.18.0): reads containing more than 10% of unknown nucleotides-(N) and reads with less than 50% of bases with a quality value (Q-value) > 20 were removed. Paired-end clean reads were merged as raw tags using FLASH 32 (version 1.2.11) with a minimum overlap of 10 bp and a mismatch error rate of 2%.

The noisy sequences of raw tags were filtered using the QIIME 33 (version 1.9.1) pipeline based on specific filtering conditions 34 to obtain high-quality clean tags. The filtering conditions were as follows: briefly, raw tags from the first low-quality base site where the number of bases in the continuous low-quality value (the default quality threshold is <= 3) reached the set length (the default length is 3) were broken. Then, tags whose continuous high-quality base length was less than 75% of the tag length were filtered.

### OTU and community composition analyses

The effective tags were clustered into operational taxonomic units (OTUs) with at least 97% similarity using the UPARSE 35 (version 9.2.64) pipeline. The tag sequence with the highest abundance was selected as the representative sequence within each cluster. For the analyses between groups, Venn diagram-based analyses were performed in the R project VennDiagram package 36 (version 1.6.16), and an upset plot was developed in the R project UpSetR package 37 (version 1.3.3) to identify unique and common OTUs.

The representative sequences were classified into organisms by a naive Bayesian model using the RDP classifier 38 (version 2.2) based on the ITS2 39 database (version update_2015), with a confidence threshold value of 0.8. The abundance statistics of each taxa were visualized using Krona 40 (version 2.6). The stacked bar plot of the community composition was visualized in the R project ggplot2 package 41 (version 2.2.1). Circular layout representations of species abundance were graphed using Circos 42 (version 0.69-3). A heatmap of species abundance was plotted using the pheatmap package (version 1.0.12) 43 in the R project.

### Statistical analysis

The random forest package 44 (version 4.6.12), pROC package 45 (version 1.10.0) and labdsv package 46 (version 2.0-1) were used in the R project. A ternary plot of species abundance was plotted using the R ggtern package 47 (version 3.1.0). Chao1, Simpson and all other alpha diversity indexes were calculated in QIIME 33 (version 1.9.1). Comparisons of the alpha indexes between groups were performed with Welch's t-test and Wilcoxon rank test using the R project 48 (version 2.5.3).

The R project 48 (version 2.5.3) was also used to analyze the data based on multivariate statistical techniques, including Jaccard and Bray-Curtis distance matrixes, principal component analysis (PCA), principal coordinate analysis (PCoA) and nonmetric multidimensional scaling (NMDS) of weighted UniFrac distances, and the results were plotted in the R project ggplot2 package 41 (version 2.2.1). Welch's t-test, Wilcoxon rank test, Adonis (also called PERMANOVA) and ANOSIM test were performed using the R project, and the functional groups (guilds) of the fungi were inferred using FUNGuild 49 (version 1.0).

## Supplementary Material

Supplementary figures and tables.

## Figures and Tables

**Table 1:** Difference of alpha diversity index between the GC and control groups

Group	Testing method	Index	P value
GC-VS-Control	T-test	Sobs	0.001267
		Shannon	0.000003
		Simpson	0.000156
		Chao1	0.001763
		Ace	0.006286
	Wilcoxon	Sobs	0.011800
		Shannon	0.000001
		Simpson	0.000001
		Chao1	0.025431
		Ace	0.044121

## Abbreviations

GC: gastric cancer
AUC: area under the receiver operating curve
*C. albicans*: *Candida albicans*
AG: atrophic gastritis
IM: intestinal metaplasia
ITS: internal transcribed spacer
SRA: Sequence Read Archive
OUTs: operational taxonomic units
PCA: principal component analysis
PCoA: principal coordinate analysis
NMDS: nonmetric multidimensional scaling
