# Supplementary material for: Candida albicans disorder is associated with gastric carcinogenesis
Source: Theranostics. 2021 Mar 5;11(10):4945–56. doi: 10.7150/thno.55209 (PMC7978306; doi:10.7150/thno.55209)
Supplement: Supplementary file 1 — Supplementary figures and tables. [file thnov11p4945s1.pdf]

Supplementary Materials

Supplementary Figure 1. Comparison of the distribution and diversity of fungi in the stomach of adjacent non-cancerous samples and healthy samples. (A) Through the principal component analysis (PCA) dynamic display, adjacent noncancerous samples (n=45) and healthy samples (n=10) are relatively close, indicating that the sample composition is similar. Hypothesis tests of the alpha diversity index through Welch's t test, Chao1 (B), ACE (C), Sobs (D), Shannon (E) and Simpson (F) diversity indexes between the adjacent noncancerous samples (n=45) and healthy samples (n=10) confirmed that there were no significant differences in species diversity between groups except the Sobs index. As for two classic beta diversity indexes named the Bray abundance index (G) and Jaccard distance index (H), based on the distance index ranking, ANOSIM (analysis of similarities) confirmed that the distance between groups was no statistically different.

Supplementary Figure 2. *Candida albicans* is an indicator by comparing GC and control samples. (A) Through the principal component analysis (PCA) dynamic display, GC (n=45) and control (contain 45 adjacent noncancerous tissues and 10 healthy samples) (n=55) samples showed clustering distributions. PC1 and PC2 represent the first two main components, and they reflect the contribution to the sample difference, expressed as a percentage. (B) The corresponding heatmap shows relative abundance of dominant gastric fungal phyla in the GC and control groups. (C)Differentially abundant fungal genus between the GC (n=45) and the control

(n=55) groups. OTUs and taxa differences are shown with p-values less than 0.01. Differences in fungal species abundance between the GC (n=45) and control (n=55) groups were detected using Welch's t test (D) or Wilcoxon rank sum test (E), and *Candida albicans* was significantly elevated in the GC group ($p<0.0001$). (F) The markers achieved an area under the receiver operating characteristic curve (AUC) of 0.699 for the classification of the GC group from the control group.

Supplementary Figure 3. Alpha diversity indexes of the GC and control groups. Alpha diversity indexes between the GC (n=45) and control groups (n=45). The commonly used alpha diversity indexes of these six categories were (A) Chao1, (B) ACE, (C) Sobs, (D) Shannon, (E) Simpson, and (F) Good's coverage indexes.

Supplementary Figure 4. Guild abundance histogram of the GC and control groups. Based on the OTU abundance, we used FUNGuild to perform functional prediction. (A) Twelve guild subcategories and (B) three major trophic categories distinguished the GC (n=45) and control groups (n=45).

Supplementary Table 1. Distribution of Candida albicans expression in gastric cancer patients according to clinicopathological characteristics.

Supplementary Table 2. The fungal profiles of GC group and control group.

Supplementary Table 3. Fungus in the tissue samples showing differential abundance between the GC and control group at the family level.

Supplementary Table 4. Fungus in the tissue samples showing differential abundance between the GC and control group at the genus level.

Supplementary Table 5. Fungus in the tissue samples showing differential abundance between the GC and control group at the species level by Welch's t test.

Supplementary Table 6. Fungus in the tissue samples showing differential abundance between the GC and control group at the species level by Wilcoxon rank sum test.

Supplementary Table 7. The composition of species at different classification levels through the species composition pie chart.

Supplementary Table 8. The values of alpha diversity indexes.

Supplementary Table 9. Guild's fungal function classification prediction.

Supplementary Table 10. Trophic's fungal function classification prediction.

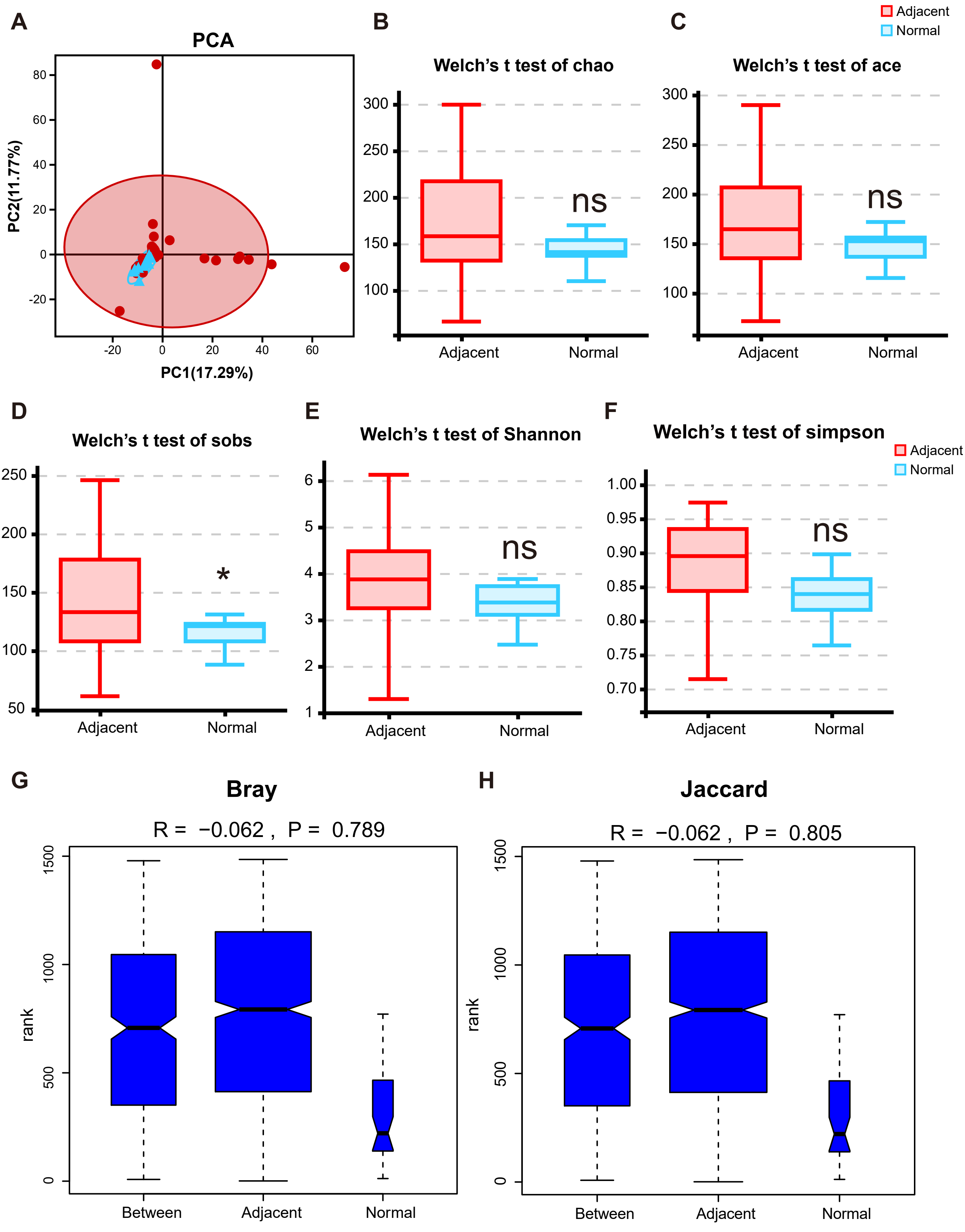

A
PCA
PC2(11.77%)
PC1(17.29%)
B
Welch's t test of chao
C
Welch's t test of ace
Adjacent
Normal
ns
D
Welch's t test of sobs
*
E
Welch's t test of Shannon
F
Welch's t test of simpson
G
Bray
R = −0.062 , P = 0.789
H
Jaccard
R = −0.062 , P = 0.805
rank
Between
Adjacent
Normal

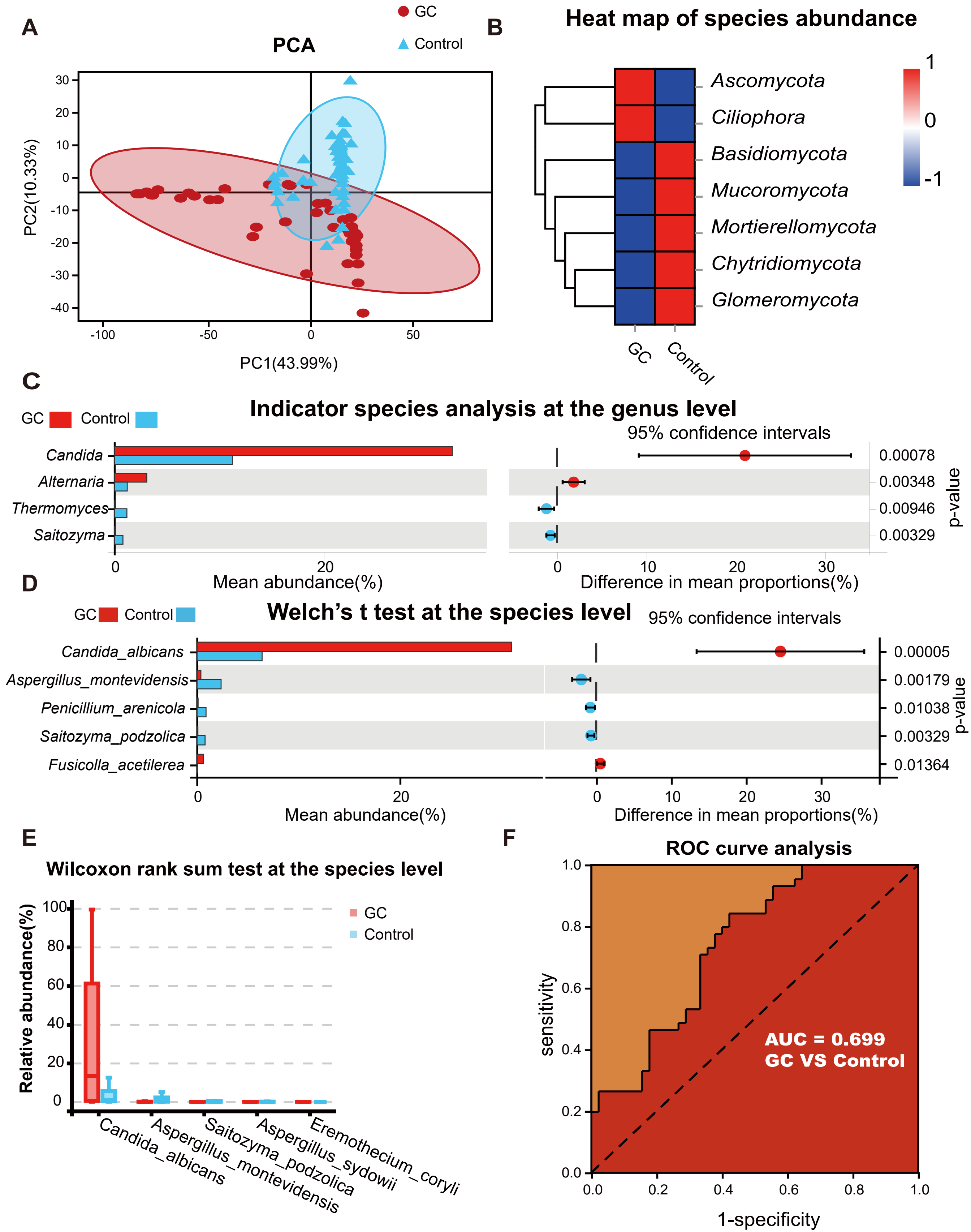

A
PCA
GC
Control
PC2(10.33%)
PC1(43.99%)
B
Heat map of species abundance
Ascomycota
Ciliophora
Basidiomycota
Mucoromycota
Mortierellomycota
Chytridiomycota
Glomeromycota
GC
Control
C
Indicator species analysis at the genus level
95% confidence intervals
Candida
Alternaria
Thermomyces
Saitozyma
0.00078
0.00348
0.00946
0.00329
p-value
Mean abundance(%)
Difference in mean proportions(%)
D
Welch's t test at the species level
95% confidence intervals
Candida_albicans
Aspergillus_montevidensis
Penicillium_arenicola
Saitozyma_podzolica
Fusicolla_acetilerea
0.00005
0.00179
0.01038
0.00329
0.01364
p-value
Mean abundance(%)
Difference in mean proportions(%)
E
Wilcoxon rank sum test at the species level
Relative abundance(%)
Candida_albicans
Aspergillus_montevidensis
Saitozyma_podzolica
Aspergillus_sydowii
Eremothecium_coryli
F
ROC curve analysis
sensitivity
1-specificity
AUC = 0.699
GC VS Control

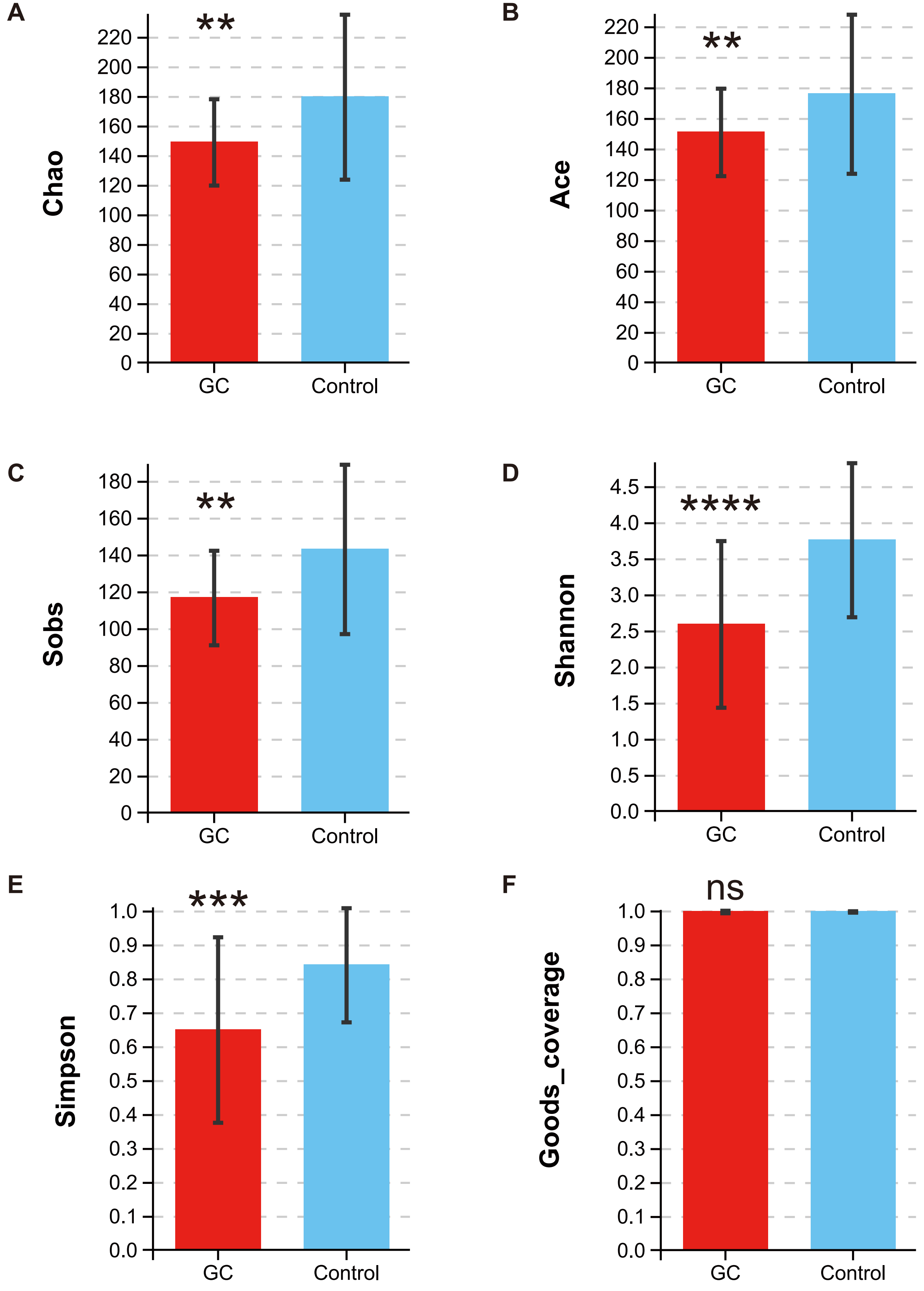

A
B
C
D
E
F
Chao
Ace
Sobs
Shannon
Simpson
Goods_coverage
**
**
**
****
***
ns
GC
Control

A

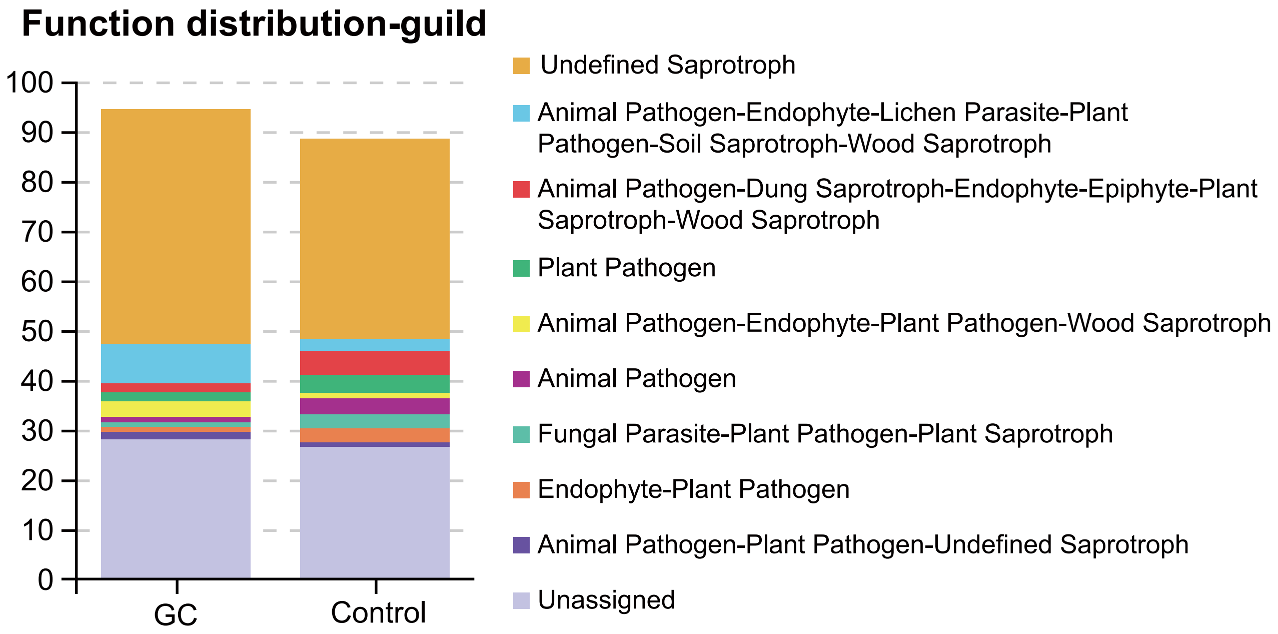
Function distribution-guild
100
90
80
70
60
50
40
30
20
10
0
GC
Control
Undefined Saprotroph
Animal Pathogen-Endophyte-Lichen Parasite-Plant Pathogen-Soil Saprotroph-Wood Saprotroph
Animal Pathogen-Dung Saprotroph-Endophyte-Epiphyte-Plant Saprotroph-Wood Saprotroph
Plant Pathogen
Animal Pathogen-Endophyte-Plant Pathogen-Wood Saprotroph
Animal Pathogen
Fungal Parasite-Plant Pathogen-Plant Saprotroph
Endophyte-Plant Pathogen
Animal Pathogen-Plant Pathogen-Undefined Saprotroph
Unassigned

B

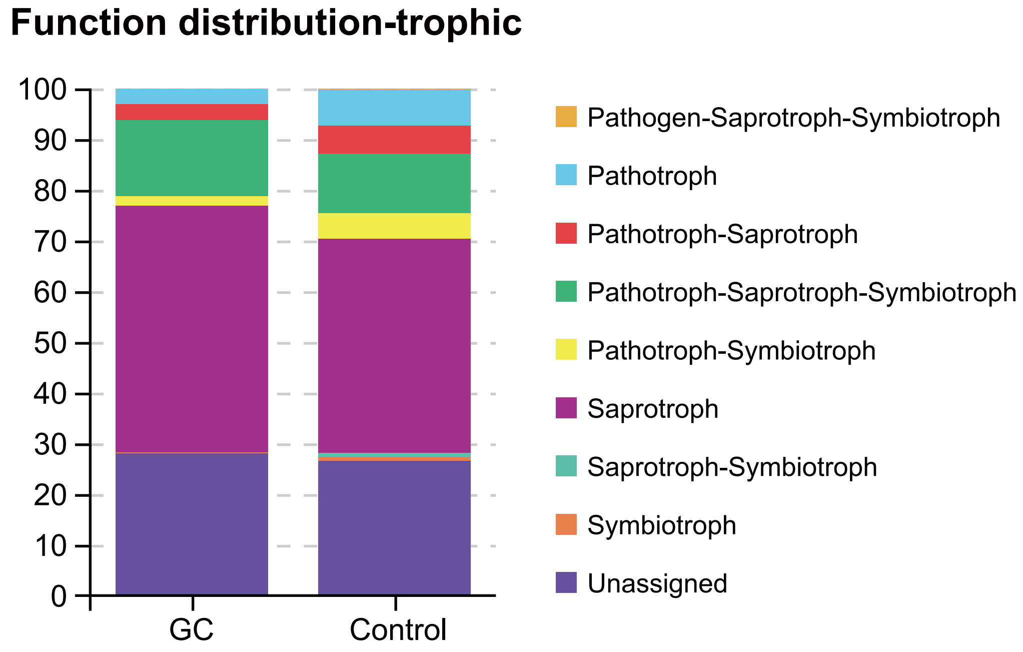
Function distribution-trophic
100
90
80
70
60
50
40
30
20
10
0
GC
Control
Pathogen-Saprotroph-Symbiotroph
Pathotroph
Pathotroph-Saprotroph
Pathotroph-Saprotroph-Symbiotroph
Pathotroph-Symbiotroph
Saprotroph
Saprotroph-Symbiotroph
Symbiotroph
Unassigned

Supplementary Table 1. Distribution of *Candida albicans* expression in gastric cancer patients according to clinicopathological characteristics.

Characteristics		*Candida albicans*		χ2	P value
		Positive	Negative		
**Gender**					
	Male	12	13		
	Female	11	9	0.2179	0.6407
**Age**					
	<65Y	10	5		
	≥65Y	13	17	2.179	0.1399
**UML location typing**					
	L/M	19	16		
	U	4	6	0.1922	0.6611
**Tumor site**					
	Anterior wall	0	2		
	Greater curvature	3	3		
	Lesser curvature	12	13		
	Posterior wall	2	1		
	Cross section	3	1		
	NA	3	2	t=0.06551	0.8218
**Diameter**					
	<6	12	14		
	≥6	11	8	0.6056	0.4364
**General classification**					
	B1/B2	1	5		
	B3/B4	22	17	1.889	0.1693
**Differentiation**					
	Poor	10	8		
	Moderate	12	13		0.6249
**Growth mode**					
	Nested	13	11		
	Diffuse	8	7		
	Clump	2	3		
	No	0	1	t=0.06768	0.649
**Nerve infiltration**					
	Yes	4	2		
	No	12	8		
	NA	7	12	t=0.08944	0.933
**Lymphatic tumor thrombus**					
	Yes	16	17		

No	7	5	0.3416	0.5589
**Vascular Tumor Thrombus**				
Yes	8	2		
No	15	20	2.936	0.0866
**The depth of infiltration**				
T2	1	2		
T3	8	9		
T4	14	11	t=0.0718	0.9462
**Lymph node metastasis**				
N0	10	3		
N1	4	3		
N2	3	9		
N3	6	7	t=0.116	0.9114
**Distant metastasis**				
Yes	16	10		
No	7	12	2.68	0.1016
**pTNM staging**				
Stage 1	0	1		
Stage 2	10	6		
Stage 3	13	15	t=0.05872	0.956
**Survive**				
Alive	16	14		
Death	7	8	0.1779	0.6732

Samples	PC1	PC2	PC3	PC4	PC5	PC6	PC7	PC8
**Ca-1**	-75.4598	-1.03825	2.33487	2.5726	0.852742	-1.19069	0.057113	-1.46329
**Ca-2**	-62.0078	-2.26946	1.486623	2.808057	1.196922	-0.15053	-0.3519	-0.78209
**Ca-3**	-1.89785	-28.9299	8.76995	-14.1535	-3.00855	3.136618	-5.29512	-2.87178
**Ca-4**	-11.7745	-10.9582	2.558914	-6.95152	-2.29933	-1.13891	-3.40445	-1.16405
**Ca-5**	-78.3147	0.111832	1.527674	2.901634	0.048672	1.867885	0.291177	-1.88499
**Ca-6**	-25.7757	-12.5307	2.597278	-8.82461	5.316217	6.351905	-1.99745	-4.27381
**Ca-7**	-82.9947	-0.52765	2.553553	3.381457	1.16099	-1.22032	0.313997	-1.65603
**Ca-8**	-81.5036	-0.53944	2.472261	3.249564	1.090464	-1.2287	0.169744	-1.58006
**Ca-9**	-76.0624	-0.96129	2.255584	2.46264	1.007096	-1.02112	-0.01855	-1.22052
**Ca-10**	-72.8639	0.731173	1.016422	4.123896	0.583242	0.449427	0.311983	-1.35332
**Ca-11**	-10.3315	2.084184	-1.8625	-1.558	-1.90338	-3.00244	3.112462	0.965057
**Ca-12**	25.07152	-43.1383	13.44644	-20.3394	-3.32921	2.260437	-6.81231	-7.02301
**Ca-13**	-44.1804	-2.75498	0.768062	-1.21906	-1.39296	1.62824	-0.95478	-0.40286
**Ca-14**	-27.5296	-15.8572	4.931017	-6.74307	-0.91685	1.3322	-2.79174	-1.72772
**Ca-15**	-55.3601	-1.34201	0.656523	-0.8053	2.239201	-0.72997	-1.22076	1.452346
**Ca-16**	-58.0667	-0.43306	0.853132	1.645679	-0.34651	0.609303	-0.0558	-0.1406
**Ca-17**	-40.7294	1.077375	-1.08655	-1.08079	1.822726	-0.65686	-0.72552	3.252185
**Ca-18**	-19.4371	2.656385	-4.20889	-3.34617	-2.81291	5.617112	-1.66902	4.898232
**Ca-19**	-48.0805	-2.8102	1.104669	-0.7548	-1.05062	1.062577	-0.78335	-0.86497
**Ca-20**	-9.25184	1.768144	0.05491	-3.26279	6.542131	-0.32522	-1.66288	0.305206
**Ca-21**	22.86104	-32.5768	10.10369	-17.0878	-3.71401	0.22069	2.183407	-6.37025
**Ca-22**	17.95117	-25.2259	5.382302	-17.8039	1.823111	2.676573	-6.14148	4.584692
**Ca-23**	22.73914	-19.6578	-7.20126	53.03886	13.68014	5.713307	3.164785	-0.80891
**Ca-24**	21.87959	-17.8536	-5.10873	37.36839	9.64664	4.063019	2.113737	0.296886
**Ca-25**	23.04587	-27.9728	0.929403	22.4982	6.328608	3.890679	0.275312	-2.84371
**Ca-26**	22.12696	-24.0701	1.664217	10.65963	2.926288	2.325039	-1.48054	-1.31372
**Ca-27**	22.07367	-21.1883	7.943242	-12.6832	-2.81702	-2.32697	26.79316	-7.67488
**Ca-28**	20.14495	-18.0116	3.038249	-10.0318	-3.09364	-1.13122	-3.53639	-0.21532
**Ca-29**	22.26732	-22.0628	-3.06162	34.35534	9.035539	4.246853	1.153626	-1.63203
**Ca-30**	21.50124	-17.2505	15.17668	-8.41943	-1.52975	1.622976	-5.08512	-2.68495
**Ca-31**	11.32945	-12.3645	0.672398	-13.4909	1.823414	1.784456	-5.17888	9.483203
**Ca-32**	-1.33537	2.060264	-0.20499	-4.85566	-3.84456	-4.83795	21.55	-2.00117
**Ca-33**	20.06831	-13.7064	-0.98297	6.245258	0.977364	0.23075	-1.40354	1.925822
**Ca-34**	19.98264	-13.8552	-0.31576	1.733093	-0.33366	0.183709	-2.0401	3.4901
**Ca-35**	18.73521	-15.1712	-0.3291	5.738486	1.057901	0.716442	-1.57166	1.162276
**Ca-36**	19.33235	-16.3753	-0.58115	9.665751	2.021678	0.832361	-1.0989	-0.5018
**Ca-37**	16.13974	-11.2506	-1.17901	6.022045	0.880581	-0.06979	-1.29028	-0.12045
**Ca-38**	10.15096	-8.11913	-2.3175	9.797041	1.824535	-0.07873	0.09011	0.295333
**Ca-39**	19.60506	-14.4662	3.082223	-9.65969	-2.07919	-1.08309	-4.16695	-0.7846
**Ca-40**	19.65876	-9.8879	6.344391	-3.20295	-1.42714	0.05069	-3.89317	-0.61403
**Ca-41**	-81.8331	-0.54306	2.490973	3.302256	1.110499	-1.23217	0.198055	-1.53901
**Ca-42**	7.55712	-4.50407	-0.98965	-5.6341	-2.74661	-1.82904	-2.38014	1.368369
**Ca-43**	3.99973	-4.27153	-1.07739	-7.06858	-2.91555	-0.4214	-2.84845	6.761857
**Ca-44**	3.202655	-7.72483	0.17032	-8.07132	0.065437	-0.86382	-2.73125	1.106137
**Ca-45**	19.62121	-15.0833	2.528596	-11.013	-3.24283	-1.35493	-5.05814	-1.1729
**N-1**	13.25853	13.88353	-9.57167	-2.09168	3.208553	-4.05069	-0.80219	10.83808
**N-2**	12.6122	15.54867	-10.7151	0.053802	-4.4261	-8.14481	-2.75933	-3.92402
**N-3**	16.18779	20.49186	-17.53	-26.9536	81.88844	13.74204	2.508033	-4.89261
**N-4**	16.25086	24.70428	27.93441	4.936858	0.8957	2.556404	-5.01175	1.643198
**N-5**	17.29897	20.3172	24.93741	2.549554	0.368032	1.882473	-5.32985	1.503393
**N-6**	20.09427	39.18039	62.53679	15.09621	5.869336	9.385629	-8.48947	-1.25892
**N-7**	10.33843	20.46547	-23.0848	-5.22438	-23.685	72.83793	3.065003	-7.97415
**N-8**	15.03936	16.14185	-11.8394	-0.17875	-5.37639	-10.002	-6.856	-21.2192
**N-9**	14.31021	15.11959	-10.9181	2.219495	-3.81186	-7.54111	-3.75761	2.625729
**N-10**	15.10926	14.14157	-10.3903	1.319467	-3.94637	-7.43035	-3.3592	1.974209
**N-11**	14.91385	6.861856	-4.46955	-4.53344	-4.58744	-5.62055	15.40144	2.12205
**N-12**	15.60106	4.064828	-2.59653	-3.43042	-3.94787	-4.94843	20.99266	-0.23698

**N-13**	14.71252	10.44585	-8.75176	-0.18776	-3.88475	-6.8742	-4.1217	-8.39385
**N-14**	15.54415	9.220652	-12.157	2.105182	-8.83605	21.95852	-0.57973	2.612317
**N-15**	14.96775	17.48632	-13.4192	1.581021	-5.66667	-11.3405	-8.33921	-25.227
**N-16**	14.1398	11.76223	-7.96717	-0.27037	-3.03349	-4.92835	-1.32084	2.623954
**N-17**	17.36227	23.96918	24.66504	1.327053	-0.16261	-0.54306	18.6908	-1.62997
**N-18**	12.72893	5.569722	-3.98456	-3.51836	-3.18265	-4.32949	-1.5907	6.517041
**N-19**	15.35336	7.886885	-7.78846	-2.79816	-4.6045	-7.34099	-5.53093	-12.7162
**N-20**	15.72867	10.98951	-9.38753	1.640444	-3.49453	-6.67028	-3.62654	1.548657
**N-21**	15.22649	13.23199	-10.4777	5.084233	-2.60184	-6.2785	-2.54087	2.815062
**N-22**	16.23511	-0.11141	-4.81641	-0.6541	-1.15802	-1.83973	-1.04411	9.036278
**N-23**	12.4898	-1.33181	-3.61996	7.887527	0.237656	-2.09338	8.531671	0.098291
**N-24**	15.35453	15.47774	-12.2068	0.404128	-5.49519	-10.4716	-7.55893	-19.4049
**N-25**	17.89667	11.07751	15.08218	-4.06644	-3.32715	-3.43457	32.71687	-4.45485
**N-26**	20.19173	17.17837	41.32007	0.856382	1.461563	5.186388	-7.73579	-1.3626
**N-27**	15.03022	14.80813	-10.9242	2.573383	-3.65854	-7.47839	-3.16343	-0.0847
**N-28**	18.75849	6.909974	20.94525	-0.91663	-0.19809	1.952899	-4.86928	-0.23136
**N-29**	15.86634	-2.113	-1.74582	-1.44073	-2.59572	-3.05716	14.87666	3.004814
**N-30**	15.93989	2.354716	-6.5248	-2.82108	12.10861	0.254212	-0.65894	1.141942
**N-31**	15.50123	7.626713	-6.17848	-2.46959	-3.23322	-4.23196	-1.34828	5.527409
**N-32**	15.14616	8.297431	-6.59884	-2.68486	-3.51173	-2.43588	-1.37574	5.962277
**N-33**	15.39996	7.893261	-6.40414	-1.63408	-2.99342	-3.87918	-1.10358	6.055687
**N-34**	15.86742	5.345956	-5.29506	-3.24209	-3.1492	-3.69868	-1.72686	5.139874
**N-35**	14.79275	10.33887	-7.81226	-2.31294	-3.48622	-3.29366	-2.0404	11.70548
**N-36**	15.52031	4.216128	-4.87659	-3.67503	-3.27871	-2.92847	-1.64294	3.784991
**N-37**	16.00425	8.220954	-8.12007	2.415645	-2.62807	-5.35818	-3.1049	0.908849
**N-38**	-13.0685	5.687536	-6.10221	11.04342	0.795224	-0.97365	0.09938	1.905739
**N-39**	-2.65333	10.97516	-6.80906	0.598765	-2.42142	-4.70467	-0.59691	7.219908
**N-40**	16.07153	12.64812	-2.25466	-2.88168	2.074123	-3.70376	-6.14856	4.467116
**N-41**	-17.1296	5.170273	-2.37625	-2.26753	-1.55762	-2.51391	13.44585	0.504399
**N-42**	-3.21951	4.002115	-3.53271	-3.29977	-2.69497	-1.66135	-2.26474	9.548821
**N-43**	0.377721	3.813326	-6.86099	-5.36691	-6.78521	11.57342	-1.74795	9.736951
**N-44**	-16.527	3.170176	-2.8042	-1.23206	-1.87419	-1.9661	-0.63316	2.302641
**N-45**	-2.8799	3.655142	-4.91632	0.951234	0.129775	-2.54169	-1.67796	5.475307

PC9	PC10	PC11	PC12	PC13	PC14	PC15	PC16	PC17
1.191356	-0.48346	0.507249	-0.47339	0.192452	-0.42522	-0.19846	0.020831	-0.1175
0.696252	-0.05936	0.18978	-0.45487	-0.24947	-0.7659	0.322965	-0.25664	0.210183
-0.11066	1.475025	-1.68233	0.987826	1.050348	1.370799	2.383185	-1.70648	-0.46359
3.598941	-1.26043	2.421535	2.929979	5.534364	-3.13912	2.912593	5.690036	4.806831
1.347432	-0.50671	0.568305	-0.58214	0.126342	-0.26122	-0.3321	0.054199	-0.1221
2.301367	-0.66576	0.713473	0.665017	1.578186	0.680208	0.106605	-0.39925	0.161659
1.241309	-0.4424	0.494337	-0.61309	0.101595	-0.33285	-0.30929	0.017001	-0.10721
1.232745	-0.47628	0.526731	-0.54652	0.159275	-0.43645	-0.29371	0.129876	-0.02096
0.726138	-0.29421	0.222366	-0.51844	0.067673	-0.24342	-0.16321	-0.05193	-0.14451
1.261368	-0.51976	0.503192	-0.46818	0.005799	-0.62854	-0.18319	-0.00707	-0.08014
2.638411	0.109063	1.287983	-0.32845	-2.25781	-2.60809	-0.58316	0.603392	0.242138
3.765461	-0.17332	0.441774	2.212156	3.393505	2.50949	1.838139	-1.60454	-0.99125
0.373248	-0.38036	0.576349	-0.25173	0.697688	-0.37142	1.29102	-0.30575	-0.13293
0.123263	0.453072	-0.25308	-0.04704	0.964277	1.423401	1.015287	-0.62131	-0.43179
-1.9476	2.231024	-2.3431	-0.84848	-0.39465	1.143216	0.622708	-0.34165	-0.17118
0.12306	-0.17817	0.36371	-0.32021	-0.38637	-1.09332	-0.60114	0.178438	0.289532
-3.84403	0.157931	1.113755	-2.47287	-1.14598	1.18005	-3.38993	-0.14068	-0.05885
-6.15304	4.084287	-4.72027	-0.98034	-1.5618	1.589965	1.36788	-0.29342	-0.45566
0.752606	-0.13191	0.088152	0.030098	0.724225	-0.1963	0.117453	0.038232	-0.181
1.821536	-0.0423	0.00693	0.515089	0.738978	-0.07503	-0.018	-0.03349	0.743117
3.836175	0.512304	-0.17257	2.006928	4.060072	2.191695	1.929712	-0.82277	-2.6632
-10.9186	5.382113	-6.62069	-0.51146	-1.17117	4.229107	3.107835	-1.37128	-0.80412
-1.91979	0.775351	-3.70485	0.687676	-1.01724	-0.23333	3.562294	-0.10551	0.558095
-2.09597	0.287464	-1.10417	-0.24304	-1.61644	-0.10955	0.497305	0.048625	0.803535
0.42586	0.091354	-0.88062	0.741893	0.116306	0.465791	1.260734	-0.32215	0.505376
0.609047	0.232581	0.152918	0.252628	-1.17268	-0.62325	-0.34301	0.311069	0.947702
1.934902	1.750167	-0.16888	0.025387	-0.22853	2.38624	1.603337	-0.61903	-0.15321
1.794405	-1.84207	5.262348	-1.36955	1.08508	2.580679	-5.24153	-0.66722	0.174833
-0.37777	0.036256	-1.77036	0.824641	-0.13245	-0.24377	2.717804	0.057732	0.629713
4.566377	-4.3171	-2.70516	-0.33281	0.182635	1.367945	0.107998	-0.12765	0.143202
-15.6599	7.603572	-10.2698	-0.41215	-3.12833	3.078325	5.249002	-1.47432	-0.88094
0.546407	1.558375	-0.87567	0.068184	0.764803	0.791766	1.149504	0.026723	-0.20776
-1.38035	-2.77895	7.169106	-3.75168	-0.29712	1.896825	-7.41947	0.220755	0.642145
-1.80732	-3.71626	12.64227	-8.21667	-2.07119	4.907749	-12.612	-0.82282	0.090629
-2.05228	0.04736	-0.01093	0.142219	-0.15388	0.017055	1.110997	-0.18599	0.010339
0.792738	-0.65737	1.231393	0.051681	1.130629	0.43998	-1.95094	-0.26066	0.38962
0.731289	-0.91549	0.378207	1.220938	1.233428	-0.83074	0.589458	-0.10698	-0.02244
0.566328	-1.12984	1.087914	0.774896	-0.44502	-3.07321	-0.99934	0.717713	1.029478
11.08509	3.222247	2.898507	-3.54421	-13.4823	-3.73106	1.141541	0.105194	-0.17864
6.013245	2.225514	-0.6693	-0.18058	-1.83301	0.041684	1.1185	-0.85275	-0.31169
1.192465	-0.5212	0.665389	-0.7086	0.107545	-0.27906	-0.40809	0.013691	-0.10405
0.877518	-2.11006	3.021347	1.439987	1.652238	-4.66239	-3.83943	4.061754	3.044852
-8.66975	3.606798	-5.11567	0.051275	-1.48612	0.380853	2.129785	-0.19634	-0.5877
-1.46471	-0.56546	-0.68414	1.148203	1.000051	-0.23683	1.550307	-0.73835	-0.52588
3.140953	-2.57263	3.249373	4.598623	5.931796	-7.2858	0.431315	10.44887	8.075234
-1.84468	-11.5151	23.42181	-14.0382	4.484864	7.023886	23.79376	-0.90858	-1.36605
-0.11746	-0.01403	-0.23678	0.933955	3.818746	1.68143	-1.74732	-1.31026	-0.52351
0.403331	-0.23873	-1.02365	1.070624	1.139966	0.922478	-2.00248	-0.84409	5.193851
-1.36961	-0.02082	0.599701	0.900499	1.113002	-1.52003	-1.12743	0.397996	0.164771
-1.32395	0.055809	0.123295	1.441261	2.518163	-0.73078	-0.45633	-0.1835	-0.3951
-3.44152	0.957257	-0.23501	0.451964	1.727296	1.288443	-0.34336	-0.19743	-0.04136
4.432982	-1.98347	2.035499	0.176707	0.988137	1.185732	-0.25918	1.050265	-0.35591
-10.1603	-2.80176	0.659676	-1.5932	-5.57373	-1.26194	1.248424	0.113325	0.133123
10.51112	8.384536	-3.9359	-0.3536	9.340172	7.487932	-1.81273	-0.88166	-0.40566
8.80696	6.864879	-3.22192	0.367902	9.451463	6.017069	-1.83859	-0.65126	-0.30918
-3.53089	1.328784	-2.11119	0.298145	0.115993	1.094726	1.465863	-1.46821	-1.0554
0.807365	-0.29083	2.149523	-0.15187	-2.7865	-5.38767	-3.11436	2.501411	2.811228

-3.24476	-1.11807	0.632795	0.653045	-1.58357	-0.32802	-0.06258	0.19679	0.145914
-0.15161	2.340036	-2.15101	0.640448	-0.65869	-2.39349	1.883064	-2.59954	1.218278
-10.3258	-1.31503	-0.28066	-2.33611	-5.30384	0.354595	0.99405	-0.04778	0.105453
1.186738	-0.82859	1.652012	0.698542	3.172399	-1.23551	-0.32572	0.08626	-0.29643
-0.407	-2.14904	-2.93853	-0.98087	-1.03955	0.721637	0.562936	0.264147	0.177271
8.997121	-36.3152	-23.4473	-7.33778	-3.98075	5.025972	-1.24285	-0.26759	-0.04099
-4.86912	-1.56879	0.745053	-0.17358	-1.72745	-1.66329	0.151371	0.604906	0.179321
8.660177	6.805204	-3.16407	-0.14955	7.833108	5.810674	-0.90197	-0.35886	-0.12006
7.894208	5.878692	-2.95947	0.356731	8.242375	5.260453	-1.31534	-0.60395	-0.27537
-7.49389	-3.32028	12.03282	-9.3722	-2.728	5.348695	-17.3657	-0.18483	0.608576
0.037615	0.244621	-0.9009	1.008066	0.478779	-1.11656	1.790011	-0.37318	-0.40157
-5.14645	1.20101	-1.17825	-1.98723	-1.92369	2.413097	0.793038	-0.16251	0.089927
-0.63418	2.179518	-1.13356	-0.33183	-0.45981	0.965044	2.157668	0.263688	-0.0439
1.275427	0.68847	2.093816	-1.02775	-1.23707	0.669719	-0.1183	-0.41896	-0.35
4.901048	3.666225	-2.2222	0.624578	6.268388	4.835736	-1.68704	-1.30954	-0.4109
0.043514	-1.90919	1.883083	0.698069	1.404575	-0.82894	0.223695	0.428988	0.377816
-5.46247	1.129761	1.701779	-3.44799	-1.97588	2.751549	-3.67012	-0.46596	-0.59668
1.084056	-2.43504	1.795104	3.387779	2.266421	-9.20261	-2.1689	6.906507	-31.1245
1.22555	-2.51123	2.439227	3.673417	2.641962	-11.5693	0.258319	5.333069	4.660033
-1.95094	-0.33144	-0.59133	2.266609	0.99	-6.20284	0.902417	3.582648	2.058401
-0.55741	-1.00882	-1.25582	2.078866	0.413749	-7.08985	0.696476	4.495141	2.768017
0.453142	-1.69723	1.1821	3.095884	1.245218	-9.7892	-0.03766	5.365723	3.710979
-8.50022	2.627985	-3.70664	1.13957	-3.95343	-7.64102	1.449143	5.14349	2.399034
1.72831	-3.53435	3.062696	4.431816	2.61378	-16.2512	-0.98497	-29.4666	0.026427
6.249532	4.071875	-1.42885	-0.0487	5.422691	3.293838	0.009704	0.127981	0.165883
2.338836	0.567185	-0.5384	0.186968	0.602394	-1.3667	0.462458	0.356007	0.108089
0.861828	-7.80626	9.781468	34.35166	-14.2026	14.42101	0.821925	-1.02344	-0.30819
23.25388	14.06333	-1.28153	-10.0082	-25.7526	-1.56391	3.241259	0.368077	-0.45767
-0.25693	1.821157	-0.3251	-0.82738	1.135473	0.863038	-2.17879	-0.83861	0.112726
-10.2402	3.38619	-3.29471	-2.10066	-1.80064	1.541718	0.299314	-0.62997	-0.63228
-13.6578	4.642688	-4.66211	-3.05923	-3.75897	3.058677	-2.16043	-0.08148	-0.32379
-0.80963	-1.02944	0.275661	0.506477	-0.02364	-1.23569	-0.50789	-0.20588	-0.509
-2.56149	3.736545	-4.07507	0.687579	2.67368	1.554518	1.875844	-0.41082	-0.45156

PC18	PC19	PC20	PC21	PC22	PC23	PC24	PC25	PC26
0.258277	-0.18395	0.189253	-0.11033	0.092634	-0.19367	0.094493	-0.14157	0.205836
0.124581	0.532195	-0.68297	3.008729	-0.64049	0.240763	-0.30294	-0.66868	0.24617
1.171924	0.885768	-0.06905	5.688176	0.022388	-1.0236	-1.64001	-0.49316	0.696871
-7.82049	6.093807	-4.8962	2.710172	7.68813	3.934212	4.081084	1.626243	0.898188
0.119254	-0.21573	0.348174	-0.37751	0.131019	-0.1326	0.076978	-0.09963	0.367816
0.968924	-0.87425	0.833686	-0.75556	0.093243	-1.35001	-0.66346	1.396236	0.257666
0.15759	-0.12473	0.268482	-0.08936	0.07918	-0.09998	0.069578	-0.12974	0.378418
0.018426	-0.05215	0.153434	-0.10813	0.137305	-0.04663	0.109542	-0.09538	0.375988
0.070912	-0.39805	0.180865	-0.11939	0.14979	-0.20759	0.056332	-0.03597	0.381952
0.096925	-0.09927	0.191905	0.352418	-0.06882	-0.04137	0.093746	-0.24692	0.14402
0.737095	-1.92233	-0.47192	-0.34057	-1.82483	1.208105	0.914478	-0.58524	-1.50995
2.910365	-1.5472	2.316216	-0.6187	0.291099	-1.3763	-2.06671	-1.56574	-1.70975
0.416475	0.175914	0.285912	1.103183	-0.11593	-0.43442	0.209435	0.198499	-0.02016
0.59796	-0.67738	0.375979	-0.24742	0.73135	-1.08769	-0.48793	0.470105	0.645234
0.012215	0.650935	-0.08633	-0.36217	0.597035	-0.67078	-0.15685	0.243419	1.299151
1.822186	-0.31532	-1.02964	0.860244	-0.52001	1.213879	0.381571	0.253577	-0.58577
-0.6508	0.86693	0.500708	-0.5675	0.13188	0.273489	-0.23289	-0.17241	0.530041
-0.30744	0.272974	-0.78229	-1.34928	0.770412	-0.76329	-0.32938	0.907795	1.792416
0.126324	-0.15776	0.430039	-0.45212	0.356953	-0.50244	0.302046	0.280828	0.23064
-0.01453	-1.13816	-0.26687	0.715634	0.682096	-1.31689	0.206885	0.839554	-0.63578
0.960453	0.246678	3.134797	0.121038	0.087748	0.637806	-1.81008	-0.77426	-1.72334
-0.06616	0.952016	0.245872	-1.12443	2.5288	-1.05948	-1.19455	1.166677	3.163577
-2.73813	0.640073	0.703222	-0.77555	1.024845	0.338675	-0.67837	-0.68372	1.07449
-0.17538	0.038127	-0.40708	-0.53156	0.12801	0.622492	0.379652	0.222237	1.402652
1.147336	-0.8014	-0.34059	-0.46691	0.163737	0.110817	-0.35359	0.311063	1.184008
1.030471	-0.94996	-1.17674	-0.92514	-1.2606	0.008845	-0.49918	0.09179	1.367381
-0.7765	1.405791	2.739236	0.834624	-0.98534	-0.16504	1.34689	0.923021	1.816712
0.307905	-2.45848	1.013898	1.478878	0.997714	-1.51851	1.528626	2.269357	0.183527
-0.99051	0.112674	0.049534	-0.73555	0.457726	0.001552	-0.45597	-0.24943	0.890047
1.80806	-0.42748	-0.35519	-0.24223	0.599045	-1.56397	-1.36134	0.654168	0.797581
0.08951	1.05103	-1.03263	-0.37118	1.205293	-1.55659	1.853146	-4.39171	1.244063
-1.68681	1.76135	2.668406	-0.0319	-1.59574	0.720749	0.668547	0.845175	-0.80623
-0.26615	0.558368	0.397621	0.7705	1.479177	-0.88628	0.392242	0.751104	-1.53542
0.28111	2.07712	2.106387	3.7445	4.060051	-0.11191	1.512816	0.494449	-2.4489
1.344618	-0.89749	-0.27135	-0.53495	0.195304	-1.46023	0.487707	1.225362	-0.86083
1.103193	-1.21785	-0.13552	-0.54078	-0.89193	-0.66145	0.174768	0.593622	0.245366
-0.06845	-1.63491	0.201832	-1.25275	-0.93296	0.698337	-0.57875	-0.52963	-3.23485
3.623737	-1.85331	-1.8871	-0.31536	-2.60062	-1.84824	3.50752	0.69513	-1.42757
2.659561	-5.68324	-1.26835	-1.73549	-6.4179	9.135075	13.3063	2.997298	6.64545
3.580955	-1.60668	1.418496	0.68937	-2.37765	6.120886	-1.07084	-4.99692	-4.48371
0.160879	-0.09782	0.27094	-0.04154	0.129926	-0.10573	0.119499	-0.12972	0.325669
-2.82786	1.352804	-2.08124	-3.78912	-17.2485	-4.84479	-8.28464	-5.42872	2.08915
1.644654	0.566777	-1.83968	-2.62028	1.767715	2.920248	-1.75763	1.632045	-4.1872
-0.41582	-4.95221	-0.10644	-0.76961	1.535716	-6.0642	0.162841	4.207229	-8.7809
-15.3487	8.148939	-9.50294	-3.00237	1.836851	2.876137	-0.34009	-2.49753	0.510392
1.469051	1.058855	-0.91271	-0.90939	-0.94195	-0.20059	-0.88047	-0.63325	1.124882
-7.10992	-18.3968	-6.52756	3.048983	2.362418	0.867524	-2.51967	-1.26534	3.292961
0.49526	-0.50492	0.160695	0.464337	-0.47303	0.471665	0.384555	0.088512	-0.03369
2.334395	-0.81578	-1.99059	-1.28051	-0.8497	7.094153	-1.94498	2.471106	-7.76405
1.301165	-0.28495	-0.39527	-1.26262	-0.23951	-8.71738	12.4814	-14.2753	-1.84904
-0.85733	0.135363	-0.11799	0.23038	1.633547	0.434467	-2.19927	1.548115	2.800753
-0.92305	-1.71244	1.800043	-6.93704	1.576346	-0.42066	0.775161	0.342896	-0.38459
1.054004	3.341562	1.516116	-0.48753	-0.10963	1.022034	0.733677	-0.41272	-1.27172
2.044992	4.248454	0.392102	-0.68592	0.076452	-2.05724	1.26106	1.297081	2.520412
1.76075	0.527162	0.549466	-0.76493	-0.24521	2.073473	0.110054	-0.47571	1.421528
-8.15773	-13.8968	-3.24365	2.715446	2.243969	-1.54556	-0.44579	-0.25868	0.385494
18.58216	1.314456	-17.9444	-4.5804	6.209803	-1.7403	-3.20436	-2.05036	1.261828

-0.39107	-0.75041	0.022896	-0.09242	-0.30563	-0.60286	-0.53723	0.213849	-0.93814
3.266938	4.54573	-4.95201	23.85773	-3.83317	1.425725	-1.44449	-1.95606	-0.65904
0.357477	2.286242	0.500063	0.178641	0.594096	0.287107	-0.27814	-0.32199	0.883279
-0.43943	-4.30184	-1.15961	-0.2071	-1.78188	1.111333	0.184544	-0.7984	-1.06775
-2.45435	1.771267	1.877927	1.035861	-0.43436	0.25145	-0.29077	0.962717	1.476147
-0.31604	1.75696	-0.30975	0.038748	0.50812	0.707211	0.055907	-0.62183	0.546463
-1.07371	-3.62582	-0.89955	0.392992	-1.09257	-0.80413	-1.33928	0.326548	-0.75232
1.820972	3.59495	0.193667	-0.98375	-0.01006	0.274345	-0.17277	1.095348	0.179791
1.0826	2.003476	0.004824	-0.69284	-0.5385	0.175479	0.560172	1.225243	1.696353
-2.33827	2.011148	0.953205	0.271625	-0.09664	1.516678	-0.93677	-0.79361	0.364464
-1.74768	-1.21076	2.263135	-0.42704	-0.5486	-0.11226	-0.01673	-2.4727	-4.0772
1.51157	5.511563	1.593856	-0.4388	0.526166	-0.17827	0.641829	0.431109	0.832789
-2.88396	3.526835	3.370127	0.736822	-1.08072	-0.45493	0.660987	1.392096	-0.49575
-0.59196	-0.56116	0.133659	1.475375	1.105526	0.688555	-1.03895	0.773603	1.167669
0.165307	-2.82898	-0.80231	0.036291	-0.00353	0.521363	1.128942	-0.328	0.904619
2.935456	-1.83643	-1.5392	-1.44477	-4.1605	-1.59362	-3.41881	8.330893	4.003146
-4.2692	1.875787	3.203492	1.032989	1.143239	-0.47281	0.764109	-1.31519	-0.35646
-1.69637	3.428835	-1.76154	0.502005	0.917285	0.541304	-0.48762	-0.12262	1.492741
5.542849	-3.51811	13.68392	1.143194	5.813787	3.113747	-4.67807	-5.31318	5.813211
0.752314	-0.23174	2.21855	-1.08552	1.647364	0.435225	2.748513	2.197059	-5.04578
0.276829	1.172706	3.294593	0.971216	-3.35149	-5.48981	4.241724	7.901102	-3.71032
4.841481	-2.42717	10.38635	1.222197	4.267819	-1.55431	-1.94113	-0.50051	3.55536
0.622312	0.97544	-0.30471	2.095493	-2.7731	-1.75517	2.658056	5.16025	0.682108
-4.0895	5.11344	-1.0658	-3.34516	1.313113	0.147422	-0.2414	0.675566	1.507681
0.243385	3.543217	-0.08457	-0.00038	0.321793	-3.12026	0.861458	2.049295	1.588733
-0.65301	0.433863	0.783855	-0.51705	-0.17202	-5.00639	0.108331	1.724129	-4.47376
0.202321	2.162167	0.291946	-0.05901	0.385963	0.103277	-0.42815	-0.11679	0.752163
-5.86959	2.058977	0.416584	-0.89168	4.075031	-4.24759	-6.32807	-2.02821	-2.29339
-0.58727	1.667269	2.684879	-1.67988	-2.72447	4.130153	-0.96433	-2.31979	0.041091
-0.47948	1.603135	-0.08983	-2.45834	-0.25209	1.475663	0.513149	-0.96373	2.025597
-1.73979	1.395008	-0.96002	-3.82735	-1.46654	0.967738	-1.66633	0.365014	4.368975
-0.73017	-3.88349	-0.89325	-0.09063	-0.06507	-0.83396	0.686678	0.507637	-1.47596
1.537094	-0.37949	1.323617	-3.07301	-1.84278	11.10262	-1.92827	-3.11508	-7.48138

PC27	PC28	PC29	PC30	PC31	PC32	PC33	PC34	PC35
-0.43153	0.070738	-0.36409	-0.0758	0.091028	0.497568	-0.55913	-0.07889	0.328189
-0.09361	-0.04829	-0.39607	-0.19949	0.453466	0.224444	-1.06938	-0.15028	0.726962
-0.25415	1.31069	0.1781	-0.63103	0.975346	-0.45924	-0.54302	1.748108	0.61583
0.352768	-1.23918	-0.28485	0.856064	-3.23951	2.756484	10.55249	2.516747	-6.97078
-0.51825	0.118399	-0.4083	-0.04642	0.029894	0.452958	-0.57352	-0.10578	0.320425
-0.2417	-1.51798	0.366397	-1.37322	0.066101	0.536832	-0.19326	0.884188	0.557492
-0.51077	0.099212	-0.43091	-0.06212	0.084055	0.494693	-0.63031	-0.11202	0.335923
-0.49382	-0.02319	-0.39202	-0.0579	0.037685	0.501817	-0.47842	-0.1207	0.246687
-0.34435	0.064271	-0.34224	-0.06302	-0.11998	0.360823	-0.60334	-0.08623	0.434301
-0.37476	0.013739	-0.39238	-0.06126	0.165737	0.572557	-0.59842	-0.07063	0.424476
0.736357	-1.07599	0.372423	0.500754	-2.30028	1.493447	-1.59157	-0.72307	-1.0552
-3.24784	-0.45356	1.483993	3.093853	-1.19295	2.929912	0.401394	-3.77394	-0.34614
-0.42422	0.486855	-0.10987	0.027004	-0.41082	-0.4279	0.099431	0.301356	0.193264
-0.40263	1.042601	0.331495	-0.25519	-0.02835	-0.84067	-0.09117	0.589404	0.371209
-0.14919	0.451284	-0.16724	-0.12382	0.241006	-0.15119	-0.8046	0.024228	0.242202
-0.13952	0.686428	0.515049	-1.71601	-0.90343	0.021363	-0.07965	-1.10577	-0.90875
-0.22834	0.004582	-0.17448	0.291359	0.506214	0.348576	0.542715	-0.6935	0.255951
0.904766	0.413034	0.260947	0.461257	-0.32361	-0.04744	-1.05417	-0.75087	-0.77053
-0.09827	0.326921	-0.09685	-0.10278	-0.19645	0.539035	0.015501	0.191114	-0.30817
0.928754	1.033321	0.579673	-0.21993	-3.154	-2.6797	2.61144	0.337738	2.526127
-1.84999	0.30676	0.001411	-0.99845	0.75553	-1.50085	0.608258	3.302023	1.12633
-0.70316	2.099005	0.039985	0.113952	1.506712	-0.11487	-1.14732	2.244362	-0.19654
-0.11023	0.26747	-1.59554	-0.72478	0.707852	1.554693	-0.1095	0.856128	0.962564
0.051644	0.179119	-0.86066	-0.65624	-0.141	0.167011	0.493538	-0.59064	0.174038
-0.42063	0.441248	-0.82525	-0.90479	-0.83551	-0.78159	0.329488	-0.00166	0.027638
-0.16206	0.266294	-0.17799	-0.58183	-1.1722	-1.71722	0.463129	-0.94304	-0.31493
0.165978	1.110451	0.40971	-2.48771	0.961217	-1.51264	0.868262	-0.37725	-0.08076
0.933055	2.245701	1.274711	0.816917	-2.52728	-4.99644	3.45355	-0.8255	2.151379
-0.11133	0.483696	-0.7879	-0.81864	0.051564	0.3632	0.01995	0.453598	0.545631
-0.72434	2.18959	-0.28396	1.339738	-1.02638	-0.69625	-0.60685	0.875164	-0.72227
-1.01937	-1.07391	0.312433	1.271199	0.954124	2.19824	-1.07497	-2.50616	-0.94564
-1.1667	-2.04426	-0.2009	-1.1865	0.287219	-2.39407	2.027089	-0.71204	0.040169
0.441249	-0.09212	-1.36168	-0.64043	0.730159	-1.00568	-1.9434	-0.18248	0.640539
-2.41936	0.552344	-0.17879	-0.68882	1.732206	2.675205	-2.89426	2.430163	1.303172
1.052104	0.506425	-0.166	0.022457	0.46981	-0.74661	0.769766	-1.59317	-0.19125
0.104845	0.436767	0.60433	0.385579	-0.44532	-0.97875	2.023022	-2.13014	-0.19581
0.500725	-1.82564	2.567484	-0.05575	-1.63664	-3.70386	-0.77288	6.23106	0.840182
-3.01111	-1.05297	1.297363	-2.27728	-1.15839	-2.6525	0.952222	-2.14917	-5.37982
5.557637	2.418904	0.269396	1.133112	3.35212	1.339857	-1.75377	0.913775	0.335042
-3.90258	-4.09381	-1.74863	6.977447	-1.51121	3.342743	-0.94109	-4.73773	-0.58732
-0.51647	0.093943	-0.41088	-0.08762	0.069464	0.530841	-0.62866	-0.06252	0.308119
2.914693	5.603656	-1.85147	-0.01321	-0.85055	3.064019	3.552172	3.380782	-0.66682
0.564091	5.389599	4.539181	-7.33972	2.437114	4.269707	1.445654	-2.168	4.851575
11.92828	-2.58391	-11.6501	-0.8382	4.181677	-1.19187	-1.21458	-1.39036	-3.69689
1.238117	-1.6382	0.600637	-0.38036	2.307976	-2.7579	-7.9592	-3.45451	3.551996
-0.64749	1.013405	-0.30606	0.035219	0.018687	-0.34986	-0.27309	-0.4807	0.004149
-6.28086	1.204596	-0.25338	-1.37988	4.689517	-0.86243	2.411132	0.415114	0.113416
-0.23854	0.132175	0.361904	0.209099	-0.04786	0.376274	-0.11985	0.005423	-0.08445
1.80442	6.980068	4.478478	-7.42209	-2.33564	0.065323	-0.32103	-3.97963	-4.36626
-0.79013	-3.01123	1.439516	-5.24345	-0.74224	-1.17284	1.08335	0.847509	0.569782
0.01705	1.970007	-2.57606	4.789195	0.881622	0.026002	-0.59785	1.465849	0.440807
-1.15096	0.898616	-0.35553	0.104259	-0.38857	0.608368	-0.45665	0.559925	-0.09093
2.047093	0.118554	-0.02146	1.378699	-3.65845	-0.91648	0.380293	-0.29723	1.983927
1.507791	1.145136	0.075301	-0.11511	-0.4654	0.642116	-1.56032	-1.02525	0.184515
-1.45465	-0.73126	-1.76623	-0.12279	2.290018	0.897524	-0.53071	0.025727	-1.21798
4.459844	1.335643	2.455155	2.424179	-9.07169	1.990016	-2.99837	0.607767	0.064213
-0.19451	0.490466	-1.11655	2.483132	0.096155	0.201161	-0.86854	1.739165	-0.02154

1.131025	-0.311	0.549003	0.521039	-1.12077	-0.10115	-0.32535	-0.18489	1.101702
2.584036	-1.17261	0.164408	-0.95785	0.878479	-1.09397	-0.3591	-0.37915	0.904149
-0.97741	0.377793	-0.14038	-0.53743	1.841123	0.108783	0.060897	0.074775	-0.61262
-2.3858	-0.67194	-0.20409	-0.49436	4.494676	2.038022	0.045137	-0.58619	0.112894
0.10783	0.969939	-1.64821	2.091428	1.441227	-1.23569	0.473822	-1.44557	0.822752
0.119409	-0.12657	0.061154	-0.51516	0.295275	0.361781	0.027264	0.122144	-0.21929
-3.65735	-2.49742	-0.19594	-2.35667	6.729025	1.465327	-0.82511	1.248394	-5.06289
0.216456	0.611886	-0.01639	-0.7262	0.148694	0.631247	-0.83794	1.424026	-0.26416
0.555009	0.394625	-0.739	-0.35947	0.158047	1.725185	-0.55469	-0.81361	-0.18605
-0.1066	-1.03622	-0.29214	1.020397	-0.14101	-0.79166	2.507119	-2.98646	-0.19661
0.143227	-1.7311	1.087684	4.549507	-0.88515	4.467674	-0.14285	-6.01648	0.323246
2.142758	0.85267	-0.04292	1.042554	-3.53051	0.06426	-0.34504	-0.29399	0.660657
-1.6731	0.744431	-0.42804	-2.18337	3.392283	-1.04258	1.032533	-1.16637	1.525344
-0.24994	1.194728	-1.64916	3.210416	0.603702	0.785337	-0.20465	2.014022	0.316901
0.606504	0.268306	-1.76878	0.177136	-2.2554	-3.40799	0.004143	-0.66947	3.960671
1.162862	-16.0778	4.864262	-5.21139	-2.18379	2.232162	-0.29286	-0.06534	1.897651
1.694216	-0.75261	2.144404	-0.93583	-2.06563	7.495747	-5.35421	7.050691	-4.014
0.166348	0.092715	-0.32729	0.080327	-0.13217	-0.23311	-0.0525	-0.35659	-0.22618
3.946417	-0.58122	0.297306	-0.98547	0.586365	-1.2847	-0.54405	-0.57272	-2.25985
0.260939	-1.45511	-2.74909	1.564902	2.456494	6.022139	6.739012	3.229864	8.763892
-10.3988	2.653185	-1.63766	1.965144	-2.52233	-2.67702	-4.24724	0.300079	-1.88464
0.789206	2.027913	0.969406	-1.85131	0.172823	-0.25575	-1.66264	-0.91218	0.857344
-4.83569	0.773574	-2.21736	1.953098	-1.89483	1.047456	0.290619	0.943033	-0.03229
-0.72074	0.44832	-0.47124	0.021584	-0.06939	0.145802	-0.25935	0.117544	-0.16109
2.008472	1.758175	1.312808	-0.10336	-1.42551	2.590516	0.168474	-0.60427	-2.06549
4.886031	0.256066	13.971	9.035089	7.738425	-2.89755	1.269134	1.393092	-1.49732
-0.35977	0.236857	-0.19235	-0.01433	0.337236	0.126256	-0.27979	0.051024	-0.2517
-2.0354	-1.28584	-0.31026	-2.56483	-0.89421	-1.38043	1.551169	0.244818	-0.03652
-0.5668	-2.78395	-1.81908	1.00013	1.047629	-1.86654	2.179565	-1.85728	-1.05087
1.250788	-0.87986	-0.90311	0.631136	0.970562	-5.33029	0.597736	1.229789	-0.59534
0.832782	-2.77765	0.170302	0.927881	-0.29113	-2.44392	2.748765	-2.60756	-1.9087
1.332627	0.356583	0.265141	0.432022	-3.45464	-1.73613	0.594298	-0.84335	3.019704
-1.35344	-3.37308	-0.8652	0.810429	-1.67315	-4.91317	-1.46326	7.350618	-0.42072

PC36	PC37	PC38	PC39	PC40	PC41	PC42	PC43	PC44
0.05322	-0.15666	-0.16587	-0.0188	0.145415	0.022595	0.09109	-0.05722	-0.07165
-0.1792	-0.06581	-0.48702	0.02887	-0.02969	0.180395	0.300963	0.07438	0.122794
-0.43027	0.205805	-1.68903	-0.1029	-0.4673	0.599102	0.268613	1.337414	-0.21857
-2.89105	-1.48943	1.875787	-1.23354	0.286959	-1.88788	-0.96717	-1.04542	1.066128
0.122037	-0.18114	-0.16545	0.016965	0.282182	0.048124	0.187244	-0.15825	-0.06318
0.01759	-0.30346	-0.40284	-0.75072	0.025372	0.584747	0.065136	0.356686	0.273048
0.139209	-0.21278	-0.16505	0.038895	0.282878	0.110282	0.185226	-0.14897	-0.03591
0.061487	-0.18836	-0.1894	0.00988	0.271487	0.057049	0.224314	-0.14533	-0.01865
0.172648	-0.22867	-0.1017	-0.01024	0.237358	0.015033	0.015475	-0.12178	-0.12405
-0.04805	-0.09943	-0.17292	-0.07533	0.100768	-0.01441	0.133805	-0.06976	-0.01643
2.211969	0.073162	-0.53717	-0.1582	-1.84935	-0.24406	-0.95426	0.992572	1.658726
-1.13529	1.471194	-0.83235	-0.99609	-0.01752	-0.05378	1.42378	-1.47661	-0.02945
-0.60769	0.183153	-0.28245	-0.95298	-0.36206	-0.51293	-0.93757	0.745306	-1.04751
0.742448	-0.08014	-0.3506	-0.36613	0.13455	0.292604	-0.14435	-0.00188	-0.2854
0.479213	-0.31745	0.099948	-0.00131	0.349833	0.373264	0.324517	-0.32912	-0.17195
1.431454	-0.33839	-0.18102	0.10032	1.040935	0.388006	0.368328	-0.24643	0.68003
-1.31008	0.032102	-1.00275	1.260813	-0.37869	-0.33023	0.055245	0.313294	-0.58344
1.416513	-0.49517	0.727271	-0.55107	-0.98362	-0.17021	0.575862	1.34105	-0.72134
0.139396	-0.32374	-0.02436	-0.49011	-0.61238	-0.60984	0.242887	0.422869	0.055183
-0.28939	10.28902	10.102	4.054182	-3.60056	3.92816	3.254856	0.958506	1.634493
-0.752	-0.27989	-1.79147	1.206092	0.09667	0.914631	-0.26464	1.471068	0.03221
0.738475	-0.58814	0.057715	0.695534	1.006591	-0.04495	-0.07277	2.337659	1.449685
-2.66092	0.067235	0.77969	-0.77331	0.682826	0.442271	0.461764	0.437443	-0.47425
0.161966	-0.19221	0.273549	-0.33998	0.972835	0.437006	0.207241	-0.14416	-0.09903
0.761154	-0.23479	-0.29727	-0.32054	0.686817	0.880251	0.21132	-0.392	0.575194
1.486065	0.277065	-1.77313	1.180301	0.234001	0.520043	0.004468	-0.16213	0.233209
2.514875	-0.21927	0.216973	-0.63926	1.956196	0.69393	0.586877	-0.06646	-0.2112
2.494036	1.028453	0.996103	-2.47282	2.432809	-1.72294	-3.15853	-0.97035	-4.5081
-0.78304	0.04037	-0.00216	-0.33495	0.216708	0.338809	0.230889	0.269574	-0.14566
1.204206	-0.85098	-1.53292	0.634371	-2.1643	0.817939	0.914388	0.843525	0.685222
-0.36185	-0.5041	2.327484	-0.20396	2.223045	-0.2853	1.270209	0.618295	0.746267
-1.67658	-0.09627	0.332807	-1.84249	1.425084	-1.42369	0.567335	1.906888	-0.26899
1.302745	-1.26927	2.146131	-1.54631	0.258333	-0.51919	1.045689	0.050504	1.03502
1.330589	-4.45858	5.018006	-1.57162	0.524222	0.167944	0.254172	1.690081	2.593245
2.18698	0.208084	0.539061	-1.53679	-0.19146	-1.43226	-1.31899	0.461736	-0.92326
0.905284	1.649474	-2.88398	0.353537	-1.06087	-1.08567	-1.39716	1.885198	0.145239
-1.50446	0.449682	-2.01091	2.688864	-1.97599	-5.01676	2.279711	0.983319	-1.38567
5.608965	-1.45586	-2.84017	5.879827	-5.16202	1.274584	-2.63733	-4.0225	3.460351
-2.91143	0.003069	0.739001	0.119419	-0.25592	0.111765	0.81668	0.008148	-0.87309
-0.0188	-0.01815	1.721789	0.279153	1.781137	0.94497	-0.04413	-3.58257	1.992946
0.140916	-0.27743	-0.09586	0.021447	0.261897	0.106907	0.187178	-0.12939	0.008884
-0.96477	-1.8258	2.367325	-1.48696	0.765068	0.093934	-0.22929	-0.94945	0.764077
-4.79474	-2.52991	1.795021	4.092431	-2.4814	-0.67046	-5.61218	-3.5951	0.345741
-2.2608	0.149459	0.169767	1.136632	1.873611	0.09675	-0.1689	-1.06274	1.165152
1.447281	0.8308	-0.92282	0.940032	-0.01032	0.826731	0.163284	-0.16	-0.32811
0.236717	0.092192	-0.67523	0.782806	0.541224	0.519321	0.468049	-0.51479	-0.42427
-1.84806	0.664522	-0.61563	-1.0327	3.205097	1.254862	1.125398	-2.11443	2.295748
0.071946	-0.76489	-0.971	-0.54919	0.041901	-0.4264	-0.46543	-0.25248	-0.3262
2.200657	1.876158	-1.59127	-4.15099	3.417857	1.840832	3.871676	2.187541	-0.53256
-1.3145	-0.9965	-0.09284	-0.06985	1.411881	0.446616	0.655276	0.836993	-0.49805
-0.57975	0.490488	-0.63469	1.335974	-0.78005	-0.16834	-1.89298	-0.94489	-1.81022
0.537612	-0.41686	1.056535	-0.16213	0.344897	-0.05381	-0.56625	-0.28042	0.427312
-0.92165	0.263467	-0.97648	-0.18948	0.68141	-0.79801	-1.33391	0.994246	2.678753
1.063807	-0.59927	-0.68126	-0.267	-0.31198	1.244076	0.369831	-0.97774	-0.16188
0.569297	-0.104	0.071114	0.138919	0.530987	0.820676	-0.02932	-0.25887	-0.07087
0.062345	-5.21879	-0.48822	0.866359	-2.92452	2.463363	1.56727	1.819593	-0.52294
-1.83603	0.564993	0.046178	-0.81816	0.481907	0.075637	0.128421	0.365075	-0.90733

-0.60268	-0.16844	-0.11141	-0.11977	-0.13272	-0.22745	-0.42123	0.754314	0.589912
-0.95807	0.359638	-1.05171	-0.19743	-0.86663	0.111984	0.117525	-0.34983	-0.58559
0.134873	0.808055	0.020035	-0.38248	0.344732	0.135442	0.568337	-0.04859	-0.05457
0.427062	4.253764	-1.51448	-1.44063	-2.4731	-5.60504	-2.92075	6.910602	5.074309
2.723623	-2.10532	1.333873	1.735169	-1.737	-2.9174	2.043583	-0.80731	3.574498
-0.09677	0.060805	0.069483	-0.15738	0.610273	0.250027	-0.21632	-0.03639	-0.51246
3.188754	-0.60857	3.17294	1.300277	-3.89528	-0.11304	1.04056	-0.43882	-7.92421
0.122875	-1.08529	0.851127	1.592735	-1.18058	-0.30529	0.946336	0.19979	-1.93543
-0.10632	0.573353	-0.33169	0.124677	-0.12944	-1.23922	-0.1416	1.793073	1.032963
-3.4971	-0.28654	-2.53358	3.151909	-0.73266	0.985403	1.512412	-0.03916	-1.29426
-0.52326	0.435074	2.610006	-2.93968	-2.86244	-2.48417	-0.82849	1.203276	-4.41985
-0.54772	-1.63156	-0.76671	0.452392	0.725195	1.268601	0.526309	-0.58616	1.269487
-1.74047	-0.93597	1.438491	0.54991	-0.14743	0.90198	-0.94176	-1.08592	0.139684
-0.6674	0.203464	-0.39586	0.57782	-1.10686	-0.62654	-1.67142	0.421613	0.069344
0.707676	1.399126	0.097721	-0.58357	3.467978	0.113946	-2.87239	-3.7544	-0.72456
-1.3859	-1.14586	1.650863	-1.72166	1.262945	0.577231	-0.32791	-0.27934	1.009724
1.050825	8.670124	-3.28656	-0.3864	1.815629	0.192123	-2.82166	-4.14334	-0.18027
-0.08797	-0.23701	0.169954	0.085096	0.235936	0.376943	0.181144	-0.35145	0.007121
-0.19674	-0.94129	1.022207	0.995505	0.719392	4.65118	-4.99658	3.502916	-0.57155
4.432444	0.228667	-5.5267	-1.10428	-1.95621	4.659972	3.338205	0.286252	-1.03043
-8.46147	0.232619	-1.34599	-2.17727	-0.88032	3.283666	-0.93767	0.668511	-0.37692
-1.5556	0.275384	-0.63286	-0.6773	-0.59714	-6.65683	7.05286	-5.5768	0.985765
7.087135	-0.8566	2.743344	2.965509	6.188382	-1.70024	-0.82676	1.048631	-0.63514
0.21218	-0.43164	0.283682	0.284323	0.588974	0.186999	0.406014	-0.34542	-0.02309
-0.60052	-1.59189	0.380144	0.67631	-1.77556	-1.06271	1.539581	1.760282	-0.43702
-0.16569	-0.68296	0.209312	0.374488	2.877536	1.611797	-0.08525	-0.70949	1.513073
0.039113	-0.27097	-0.12008	0.128853	0.04288	0.28811	0.205354	-0.0478	-0.01937
0.422137	-0.04272	-0.9346	-0.47653	0.23594	-0.19278	-0.39652	0.360516	-0.02692
-0.9184	0.965616	-2.39374	2.023128	0.585569	1.485273	-0.29355	1.325905	-0.90927
2.16949	0.316815	0.563216	-10.5662	-6.71739	1.592642	-1.74372	-3.51255	1.675444
-2.20925	2.188151	-3.49708	2.30913	0.574948	-0.05254	3.098068	0.976505	-1.1428
0.229071	1.420479	-0.26891	0.645115	0.734262	-5.06893	-3.9522	-0.14614	-2.21628
-0.55868	-2.89683	2.263602	1.112523	0.587441	-0.88326	0.910197	-0.28105	-0.18678

PC45	PC46	PC47	PC48	PC49	PC50	PC51	PC52	PC53
-0.11265	0.060016	0.027811	0.091882	-0.40551	-0.03788	-0.21467	0.264387	0.202858
-0.17425	-0.02612	-0.03869	-0.06984	-0.24801	-0.06468	-0.18829	0.252661	0.208321
1.116604	-0.11386	-0.27954	0.034203	0.270921	1.377686	-0.96578	-0.25821	-1.03287
1.152087	0.205437	0.268389	-2.0948	-0.79193	0.201173	0.184199	-0.63937	0.035009
-0.13917	0.049596	-0.00055	0.044281	-0.44593	-0.09778	-0.24056	0.336048	0.204096
0.772897	0.331782	-0.23182	0.12084	0.083429	1.104696	-0.51223	0.216597	0.221644
-0.1488	0.067991	9.21E-05	0.025599	-0.50488	-0.03823	-0.22645	0.324734	0.208178
-0.2319	0.103812	0.004665	-0.06223	-0.50563	-0.14915	-0.23279	0.303429	0.203396
-0.14427	0.01179	0.127607	-0.07452	-0.32914	0.045293	-0.15947	0.282236	0.089489
-0.18489	0.096702	-0.05869	0.01378	-0.39013	0.057873	-0.3291	0.340381	0.252521
1.580734	-0.31552	-1.22087	0.523519	-0.43826	-2.46857	1.37468	-7.67419	3.051769
-2.98464	2.377979	-2.07953	-0.80597	-1.34013	0.402176	0.765905	2.706573	-0.62007
-0.39343	-0.02973	0.831857	0.977323	1.357581	-1.13228	0.427046	0.997066	-0.50016
0.424734	-0.07472	0.334874	0.100317	-0.27416	-0.00692	0.216948	0.095728	-0.0753
-0.28716	0.008785	0.547574	-0.5399	-0.48601	0.097395	0.161501	-0.12828	0.142435
-0.05224	0.299304	0.099797	-0.08675	-0.69021	-0.51481	0.492736	-0.07758	0.571722
0.529594	0.380946	-0.25163	-0.1299	0.84105	2.08883	0.229206	-0.14406	-0.2879
-0.1918	-1.1445	2.159222	-0.46046	-0.96735	-1.16879	0.536999	-1.45936	-3.66963
-0.58259	-0.20622	-0.3199	0.531129	0.420344	-0.64732	-0.28419	0.093265	-0.10421
0.306679	-0.5594	-0.43004	0.187685	0.442858	0.010124	-0.0889	-0.05727	0.107622
1.492919	-0.79269	-3.10584	-0.07908	2.582309	0.322389	-4.4583	-0.74856	-0.63455
0.417946	-2.52397	2.223778	-2.32581	1.534413	2.480921	-0.31485	-1.25815	2.559806
0.374763	0.663488	-0.9976	-0.87576	1.238716	1.218726	-1.05247	0.752135	-0.62522
0.661171	0.007915	0.32025	0.063999	-0.03655	-0.70468	0.868582	-0.15137	0.196745
0.925332	0.58735	-0.65444	-0.17464	0.039593	-0.65059	1.528624	-0.49501	0.067252
0.474835	0.406328	-0.7536	0.445048	-0.37558	-0.86148	0.663592	-1.27734	0.663867
0.739714	-0.869	-0.23825	0.591093	-1.85294	0.160047	2.621931	-0.3699	0.539252
0.583835	-0.95632	3.625559	2.085634	-1.57781	-2.18057	-0.22245	1.675175	0.254619
0.259456	0.364885	-0.58161	-0.18906	0.55969	0.26835	-0.23516	0.150497	-0.19954
3.275443	4.578873	0.853402	1.745619	0.779469	0.449676	0.215646	-2.94851	-1.94255
-1.4885	-0.84936	2.751398	-0.8873	-0.1117	0.628376	0.821231	-0.01226	0.589909
-3.99296	-0.95388	-1.78947	-0.44586	5.477213	-5.46647	-2.56868	-0.19084	-0.08631
-1.27255	-0.22799	0.410214	-0.39391	-0.1204	-2.04722	1.932535	0.07098	-0.24082
-1.85631	-1.07466	0.22831	-1.6994	0.578015	-0.07702	-0.79239	-0.07187	-0.50437
0.72141	-1.27301	2.13251	2.286451	0.381555	-2.2921	0.805954	-0.29936	1.277018
3.130219	-0.99221	1.295192	1.36005	-2.13496	-0.44854	-0.03242	2.427396	1.099398
-7.01739	3.15526	-1.37321	-1.59806	-4.95986	0.157862	0.571021	-1.455	1.061647
-3.50222	-4.01643	2.106159	-0.96557	1.278895	1.323493	-2.11299	0.79917	-1.45458
-0.68971	0.198127	0.400669	-0.2182	0.260132	0.714673	-0.83903	0.486295	-0.41328
-0.2678	0.295534	-1.11521	-0.68721	0.97362	-1.56324	2.71642	0.755749	0.321747
-0.17409	0.041751	0.006339	0.037792	-0.47684	-0.01609	-0.22935	0.342225	0.209604
-0.50844	-0.91142	1.30551	-1.12138	0.036275	-0.0704	-0.06052	0.030553	-0.17557
1.109854	2.074157	-1.55409	1.307215	-0.74732	-2.73854	-0.33755	0.169453	0.497289
-0.09941	0.392537	-0.64118	0.520547	-0.70539	0.172883	-0.36683	0.059214	-0.30192
-0.39974	-0.55284	0.135449	0.725584	0.449064	-0.1881	-0.17199	0.451232	-0.13023
0.212816	0.116651	0.029614	-0.07115	-0.06938	-0.07342	-0.08181	-0.18247	-0.09143
-2.49796	0.312037	1.065318	4.126756	-0.15885	0.909546	-0.37467	-1.38033	-0.31583
-0.18732	0.201486	-0.45733	0.129536	-0.05928	-0.45205	-0.04122	0.027653	-0.21575
-0.79859	0.579205	-0.05956	-1.584	0.493178	1.606615	-0.44096	0.06913	-0.27401
1.615562	0.952521	-0.69147	0.832824	-0.10083	-0.38781	0.05181	0.087239	-0.26563
-1.05866	-2.87006	-0.59347	-0.01851	-0.24169	-0.95003	0.186654	-0.08874	0.552733
0.277287	-0.02565	-0.29712	0.15467	-0.03058	0.600094	-0.04414	0.048015	0.341682
-0.07129	-1.17909	0.107626	0.338883	0.083653	-0.43726	-0.85736	0.536748	-0.98678
0.327537	0.481129	-0.71178	-2.15284	-1.14019	0.529332	-0.26169	0.353489	1.814541
0.843707	-0.14123	-0.17419	-0.25755	0.551732	-1.48476	-0.34775	-1.78319	-1.68295
2.252993	-1.21511	-1.92595	-2.1256	-0.45626	-1.34339	0.299042	2.390601	-0.40402
0.001944	-0.11075	0.110377	-0.13026	0.227704	0.366499	-0.61273	0.48992	0.044353

0.140327	-0.06115	-0.20767	0.796772	0.374356	0.459443	-0.03957	0.20527	-0.2661
-0.09221	0.091817	0.332076	0.110061	-0.02506	-0.09878	0.068701	0.005217	0.226502
0.522084	0.532742	0.611242	-0.82815	-1.43808	-0.44872	0.654609	-1.22845	-2.17691
2.162044	-0.9963	-0.73768	-2.0782	-1.58139	-0.16817	0.80245	2.374712	0.902196
1.244507	8.565232	4.840049	-0.39317	1.247777	-0.13187	-1.7086	1.371617	0.632124
-0.57618	-1.58237	-0.6334	-0.14569	-0.33491	-0.09095	0.108132	0.273884	0.176798
1.84861	1.182066	-0.96331	-2.8641	1.921794	-0.70301	0.943581	1.293532	2.349319
0.485996	0.032225	0.504086	0.591548	-1.91454	-1.23808	1.320366	0.148948	-4.49056
-0.7184	-0.46458	0.003226	0.135336	0.402088	-0.31008	-0.03023	0.525133	-1.65007
1.6322	0.977518	-1.46595	0.406914	-0.16589	2.250905	-0.4644	-0.687	0.270222
-0.89455	-1.22418	2.216887	1.783862	-2.10701	2.878902	-5.17608	-2.74615	-0.65523
-0.83433	-0.31666	-0.46632	1.543419	0.219926	0.782949	-0.12245	0.444666	2.080712
-1.68205	-3.30763	-0.69097	-0.56559	-3.03895	3.199407	2.719209	-0.52691	-0.61653
-0.24587	-2.259	-2.03732	0.705492	0.050322	-0.97547	-0.05938	-0.16388	-0.16798
0.431293	0.787356	0.428878	-5.7725	-0.45513	1.011482	-2.52012	-0.7677	2.908662
0.962515	0.110503	1.006616	0.254856	-0.18141	0.93989	-0.21209	-0.00245	-0.26439
0.115779	1.196935	1.53147	1.397009	0.584647	-0.53929	-0.50857	0.629605	-0.27137
-0.04578	-0.05607	0.436964	-0.07375	-0.26107	-0.12083	0.301376	0.064202	0.045364
-2.87136	2.298218	1.046241	-0.28062	-0.50852	0.144051	0.33448	0.185678	-0.04348
-1.36156	-0.4885	2.070805	-0.69733	0.810132	-0.18651	0.968752	0.064931	0.185438
0.905745	0.940838	2.351773	-0.83613	-0.088	-0.49775	0.727249	-0.08957	0.532716
3.31695	-3.82342	-0.80218	-0.11775	0.579459	-0.15989	-0.35032	-0.09789	-0.05955
0.49597	1.805406	-5.9735	2.300513	-1.84495	0.956034	-1.81617	0.636266	-0.93075
0.086745	-0.09755	0.069589	-0.04742	-0.1171	-0.14652	0.033004	0.007743	-0.06302
-3.57853	0.099684	-0.68029	6.432584	3.204736	2.172272	1.672166	1.148747	3.715089
1.347579	-0.07036	-0.8462	-0.62774	0.701066	-0.12264	0.198646	-0.2552	-0.27938
0.061177	-0.03757	-0.00822	0.000567	-0.04934	-0.03049	0.015048	-6.84E-05	-0.06128
0.174693	-0.28734	0.420871	-0.21463	-0.20448	0.2091	-0.27146	0.550023	0.089198
3.693753	-0.5999	-1.75821	1.072336	0.133063	1.303551	0.647593	0.561589	0.190718
-0.46915	1.824147	-4.49796	1.156936	-0.22291	1.198177	-0.04665	0.670419	-0.43809
-1.79592	0.370846	0.20341	-1.01216	-0.05608	-3.11051	-0.32901	0.154326	-0.24565
-0.85551	1.216209	-0.27708	-2.05753	6.460168	3.980674	4.568697	-1.33319	-2.60295
2.322084	-1.75684	3.089167	2.847528	0.137548	1.058195	0.198526	1.347195	0.623151

PC54	PC55	PC56	PC57	PC58	PC59	PC60	PC61	PC62
-0.08021	-0.03706	-0.06536	-0.09311	-0.01626	-0.04568	0.03827	0.196925	0.095721
0.017891	-0.04222	-0.2218	0.113721	-0.09362	-0.19401	0.155321	0.078506	-0.21218
-0.12505	0.443695	0.514322	1.498459	-1.51742	-0.2871	0.772259	-2.03156	0.015324
0.231661	0.164708	-0.23874	0.319228	0.055399	-0.34639	0.054004	0.296255	0.249029
-0.14579	-0.09029	-0.14489	-0.12451	0.045085	-0.08913	-0.09308	0.091626	0.022302
0.16486	-0.22197	0.349301	-0.21907	0.126082	0.502574	-0.10725	-0.1903	-0.51105
-0.11316	-0.08811	-0.15432	-0.06741	0.066731	-0.16659	-0.13731	0.070679	-0.03975
-0.1901	-0.14478	-0.18021	0.02832	0.087157	-0.1679	-0.09607	0.075156	0.034622
-0.12926	-0.02395	-0.18109	0.036563	-0.05779	-0.09743	-0.18467	0.06518	-0.03277
-0.18979	-0.05092	-0.16277	-0.03758	-0.03777	-0.08633	-0.14249	0.124762	-0.08123
-1.28216	-1.19357	3.254792	0.762897	-1.80036	1.695861	-0.20669	0.529402	0.844364
-2.3977	-1.91093	3.06736	2.102523	1.390934	0.974414	1.007141	0.305218	0.225582
0.33723	0.352298	0.514112	-2.46062	-0.47688	3.586253	1.747438	1.162486	2.898951
-0.23649	0.75933	0.302107	0.89019	-0.49237	-0.14923	-0.25946	0.267746	-0.46026
0.035121	-0.01224	-0.27079	-0.1556	0.158968	-0.46356	0.145758	0.168947	0.179323
0.235004	-0.24818	-0.13455	0.030154	0.424942	-0.4641	-0.17759	-0.23175	-0.06955
-0.45764	0.043098	0.751901	-2.69539	-0.54349	-1.67836	0.772922	-0.07052	-1.4817
-0.06311	2.40998	-0.20511	1.709319	-1.08571	-1.47757	-1.88207	-1.90249	0.601638
-0.13312	0.430471	-0.02455	0.511144	0.163732	-0.62082	-0.91671	0.470734	-2.03237
0.459416	-0.21248	-0.0047	-0.17384	0.30036	-0.10827	0.149341	-0.19065	-0.01527
1.548326	2.030289	0.774277	-0.59051	-2.54164	-2.54794	-0.74595	1.674246	2.121685
1.258407	-0.53626	0.928311	-0.84215	2.4463	1.677305	-2.02026	1.413322	-1.32121
-1.5123	0.338214	0.278116	0.153155	-0.47014	1.185982	-0.36696	-0.04164	0.157479
-0.22909	0.651384	0.460839	-1.06416	0.44194	-0.09436	0.342704	-0.73882	0.086708
-0.13643	0.302907	0.486604	0.835395	0.4479	-0.47415	0.081258	0.211831	-0.69317
1.166015	0.180366	-0.03651	-0.72973	-0.51628	-0.45774	0.198122	-0.40811	0.404046
-0.76505	1.101796	1.470994	-1.04775	1.972384	-0.2217	0.819808	-2.2502	-1.90718
-1.00107	2.282938	0.313206	-1.23027	-0.60093	1.344288	-1.43986	-0.57202	0.328937
-0.28955	0.312556	0.383807	-0.36513	-0.37024	0.397735	0.223314	-0.04514	0.294153
-3.51889	-0.74148	-5.96767	-0.93542	1.898526	0.860475	-0.38665	0.807414	-0.3464
1.226184	-0.00832	-0.87889	-1.10949	-1.62977	0.110588	1.301165	0.14878	0.121513
-0.64923	-2.28857	-0.74785	-0.91423	1.872697	-0.3495	-0.74143	-0.51121	-0.72944
0.412596	2.037884	-0.47519	4.332421	0.436701	-2.26808	0.618676	3.657489	-0.86429
0.757351	-1.95683	-1.24333	0.479673	-0.10596	1.954136	-0.08594	-2.08482	1.506168
1.745879	0.258103	-0.85317	-1.23655	0.755088	-1.73226	2.901603	1.25936	1.131742
2.87131	-6.63	-1.1913	0.779094	-2.20518	-1.26608	-1.70186	-0.0964	-0.67958
2.887134	0.885027	-1.41847	-1.10348	0.280364	0.395542	-0.38159	-0.46717	-0.37034
-0.65879	0.566735	0.183898	0.041278	0.623376	0.51942	0.291108	-0.36151	0.110972
0.038372	-0.27274	0.059725	0.338719	0.248224	-0.18216	-0.09113	0.477803	0.2759
1.102997	1.576107	-1.73517	-2.34727	-1.0699	-0.52859	-2.34586	-0.24844	0.047101
-0.11487	-0.1023	-0.15834	-0.07081	0.047571	-0.13732	-0.11624	0.038859	-0.01517
0.282077	-0.12668	-0.07714	0.016165	0.141554	-0.04354	0.059039	-0.14222	0.037281
0.30006	0.354342	-0.24385	0.57168	0.83948	0.114772	-0.25325	-0.52729	-0.28061
-0.12804	-0.32237	0.238212	-0.50489	0.140594	0.351847	-0.33655	-0.52857	0.100636
-0.00972	-0.26394	0.088582	-0.3827	0.045312	0.256768	-0.08758	-0.37737	-0.03824
0.093978	-0.11851	0.015012	0.135403	0.086814	-0.0596	-0.16813	-0.05119	-0.12548
0.776734	-0.42504	0.124543	0.201261	0.230916	0.309473	0.424231	0.647399	0.14742
-0.21079	0.02296	-0.13023	0.177623	-0.18395	-0.1224	0.033561	0.037343	0.212247
0.531564	0.228647	-0.26527	-0.34827	-0.51552	0.31	0.039607	0.15668	0.04599
-0.27655	0.001773	0.319916	0.223846	0.363765	-0.03037	-0.46345	0.049092	-0.14609
0.07665	-0.27018	1.191314	0.21393	2.319298	-0.4394	-2.23527	0.87828	0.793016
0.417908	-0.09849	0.106165	-0.45674	0.025282	-0.0724	0.097692	0.036107	-0.1985
0.191105	0.557192	-0.08018	-0.11462	-1.15962	2.237323	1.157449	2.037454	-3.01334
0.36657	-0.45253	-0.32053	-0.77203	1.991246	-1.46074	2.378655	0.403288	1.978477
1.826416	-0.20881	-0.03168	-0.05343	0.426015	-1.08879	1.017768	-1.00572	-1.93324
0.258516	0.153766	0.0563	-0.47797	0.228763	-0.24305	-0.10804	-0.13409	-0.01609
0.050348	0.162711	0.308969	0.052219	-0.18409	0.027358	0.033662	-0.0531	0.003415

-0.85937	0.494841	-0.49007	0.364266	0.228202	-0.2245	-0.45311	0.566525	1.561188
-0.17325	-0.26339	0.180495	-0.00212	0.797553	-0.04474	-0.33267	0.565405	-0.27606
0.050599	-0.85052	1.179326	-0.43106	0.825097	-1.50261	-1.5478	-2.47373	1.67362
-2.51434	1.921843	-0.16215	-0.71135	1.077927	-0.74258	-0.04956	-0.33031	0.842235
1.35388	0.428115	2.14123	0.394492	-1.23022	0.005652	0.240546	-0.00984	0.246243
0.346356	0.011327	0.620553	0.112014	-0.06517	-0.11642	0.085425	-0.10735	0.047023
-0.29494	0.157339	-0.54589	0.045733	-1.05809	0.634009	0.155975	0.724465	-0.899
0.199053	-1.55119	1.88302	-1.04104	-0.46216	1.769361	-0.35573	1.693837	0.412185
0.17246	1.075736	0.509208	-0.32933	-1.32026	1.528872	-1.21286	0.87426	-1.95589
-0.9254	0.638214	1.700435	-2.25624	0.280172	-0.85772	-0.12014	0.396418	-0.79153
0.217326	0.026771	-0.21792	0.749661	1.483724	-0.42355	0.620676	0.021704	-0.45205
0.654813	0.066299	-0.69879	0.201429	1.083782	-1.09177	0.802466	0.094175	0.97959
-1.85879	-1.02381	-1.97117	-0.21242	-2.49462	-0.34941	-0.26258	2.058983	1.019033
-0.10796	0.187919	-1.00875	0.246247	-2.24571	0.490585	4.241199	-2.45126	-1.651
-2.24059	0.063696	-1.34986	1.352173	-1.49024	0.700799	-0.52682	-0.83809	-0.29864
0.322502	0.239186	-0.11288	-0.2792	0.039272	-0.03944	-0.05946	-0.04746	0.022666
0.048287	-0.03401	-0.42327	0.210052	-0.014	-0.1972	-0.09888	0.301244	0.104631
-0.0587	-0.14551	-0.07662	-0.00673	0.157904	0.143914	0.014675	-0.1389	-0.10878
-0.2745	-0.30699	0.187312	-0.13617	-0.14459	-0.45904	0.128504	0.166632	-0.09544
-0.10483	0.110447	0.03994	0.019968	0.062775	0.034595	-0.02331	-0.20623	-0.1254
0.762995	0.473708	0.510742	0.246976	0.013485	-0.25058	0.097709	-0.51744	-0.31027
0.103159	0.049462	-0.36101	-0.106	0.059326	0.510706	-0.10422	-0.28676	0.187554
-0.38636	-0.4709	0.1866	0.086831	0.33408	-0.15079	0.026095	0.440042	0.047223
-0.10374	-0.09291	0.010847	-0.1279	-0.02092	-0.15375	-0.01248	-0.00649	-0.02003
-1.36761	1.132803	-0.443	1.079562	-1.6389	-0.02154	-1.82941	-1.55621	0.156097
-0.20071	0.019988	0.186144	0.186101	-0.05353	0.144594	0.065321	-0.04029	-0.22869
0.077746	-0.05898	0.007639	-0.01072	0.019639	-0.01832	0.02655	-0.0235	-0.02917
0.093348	-0.0132	0.334768	0.208857	0.394377	-0.0852	-0.04959	0.165488	-0.13925
4.208891	1.942115	-1.51725	3.492951	1.990885	3.635816	0.020906	-0.8339	1.074959
0.710817	-0.3307	0.281482	-0.51525	-0.15379	0.25956	-0.5328	-0.03806	-0.18041
-2.69929	-0.63804	-0.86128	1.739624	-0.9862	0.208008	0.930044	-0.06046	1.217007
1.132813	-1.45455	0.477201	0.866995	0.410591	-0.9234	0.726182	-0.32631	0.487726
-2.90838	-1.31461	1.103944	0.731938	0.664994	-0.96337	0.806663	-0.35267	0.025364

PC63	PC64	PC65	PC66	PC67	PC68	PC69	PC70	PC71
0.396929	-0.14386	-0.00277	-0.08564	-0.13078	-0.07507	-0.11089	-0.13599	0.424986
0.284665	0.063545	0.1756	-0.02071	-0.13692	-0.04135	-0.21173	-0.52729	0.250521
0.294684	2.340662	-1.30973	-0.39369	2.587361	2.025915	-0.86401	-1.50618	-0.16498
0.102968	-0.0894	-0.15389	-0.06064	0.198359	-0.16985	-0.0636	-0.02708	0.039964
0.340607	-0.22311	0.116032	-0.14533	-0.24993	-0.06548	-0.14399	-0.15698	0.48271
0.626242	0.291015	-0.42227	-0.38814	-0.2091	0.428915	0.466145	-0.59211	0.615006
0.353982	-0.31227	0.124139	-0.15727	-0.2584	-0.08873	-0.09463	-0.16143	0.507238
0.331149	-0.33612	0.028462	-0.19606	-0.33717	-0.10971	-0.14571	-0.20914	0.47144
0.283435	-0.23449	0.224825	-0.12774	-0.25995	-0.10532	-0.05807	-0.10507	0.39601
0.295167	-0.19374	0.087804	-0.1706	-0.2594	-0.00762	-0.105	-0.26808	0.792627
-1.45869	-0.29995	0.222167	0.262591	0.357114	0.799823	-0.06027	-0.22025	0.683506
-0.03263	-0.68832	-0.84676	0.486789	-0.1042	0.477459	-0.32014	1.483988	0.605305
0.261555	3.44301	1.06328	1.248499	1.210106	-1.46766	-1.62379	0.929076	-1.96035
-0.44049	-0.0933	0.22946	-0.11763	0.014038	-0.05368	0.468266	-0.21328	-0.07695
0.105341	-0.47358	0.633623	-0.39785	-0.04411	-0.46723	0.430216	0.495934	0.225455
0.950805	-0.32756	-0.10267	-0.3854	-0.414	-0.77896	-0.26377	0.353218	-0.35896
-4.13831	-1.04478	-3.59736	3.189413	0.457721	-1.07951	0.057124	0.211243	-1.01095
-0.94088	0.560503	-1.43135	-2.52229	2.077734	-0.92314	-0.36567	1.87567	0.227429
-0.56465	0.733083	1.235555	0.131196	-0.35684	3.629073	2.488403	1.207603	-3.41481
-0.02202	0.043138	0.006691	0.007553	0.022246	0.096553	-0.18486	-0.03334	-0.04217
-0.01695	-1.92198	1.057065	-0.06758	-1.19094	-0.8871	-0.37644	0.193279	-0.25587
0.248565	2.390824	-1.14705	-0.59114	-1.84656	-0.87338	0.897849	-0.39352	0.499177
-1.50995	0.569504	0.461811	0.10203	0.092356	0.722346	0.086833	-0.2524	1.247046
0.167735	-0.83907	-0.20136	-0.60126	-0.59032	0.162233	-0.23879	0.269932	-0.30415
0.677693	0.073841	0.212008	-0.29153	0.224204	-0.53884	0.163327	0.487431	-1.2751
1.740492	-0.75071	-0.37506	-0.51488	-0.32399	-0.60032	0.73385	0.440695	-1.41476
0.103978	-1.13863	1.04469	-0.4119	0.596634	-1.72914	-0.80476	-0.65534	-0.75924
-2.20237	-0.12277	0.902899	0.130952	-1.43147	1.277973	0.126979	-0.42479	1.151988
-0.28564	0.252327	0.102238	0.044056	-0.19548	0.08154	0.110469	0.038426	0.310494
-0.15768	-0.62432	0.601153	0.590227	0.381563	-0.04679	-0.40246	0.038529	0.232581
0.014086	-2.39513	3.140403	1.604638	1.817836	0.783258	0.359452	0.665613	0.69729
-0.23327	0.470791	-0.051	-0.01994	1.864854	-0.09054	0.973952	0.064814	0.842158
-0.60874	1.605968	0.962658	1.940717	0.574326	-1.61094	0.051518	-0.91035	0.269607
-0.23676	-1.71216	-0.91278	-0.5443	-0.82133	0.356702	-0.61207	0.115358	-1.0228
2.859383	-0.60416	-3.52648	0.548024	0.240854	1.73677	0.184728	0.326817	0.691153
-0.90025	0.60123	0.699638	-0.08612	0.848218	-0.8615	-0.4164	0.093271	-0.00456
-0.28654	0.28357	-0.07218	0.180521	0.274317	0.236287	-0.27221	0.137852	0.359047
-0.355	0.0715	0.446674	0.548917	0.165855	0.128258	0.126021	-0.22591	0.360348
0.055263	-0.06695	-0.04634	0.100765	0.119547	0.091041	-0.04446	0.197863	0.058439
-0.40634	1.473786	-0.71301	-1.0228	-0.31168	-0.34904	0.288155	-1.09932	-0.13661
0.349836	-0.27752	0.119131	-0.14432	-0.27541	-0.11825	-0.10055	-0.14793	0.513345
-0.09437	-0.09938	0.064938	0.049422	-0.03634	-0.00667	-0.08658	-0.00789	0.021498
-0.11302	0.167969	0.115152	-0.01267	0.023739	-0.19775	-0.25492	-0.28015	-0.04454
-0.25032	-0.06039	0.17145	-0.24984	-0.14109	-0.0607	-0.11477	-0.05482	-0.14868
-0.11951	-0.08688	0.007641	-0.01712	-0.16289	0.153941	-0.05064	0.072242	-0.01283
-0.09657	-0.08766	0.016113	-0.0542	-0.02233	0.043995	-0.01019	-0.0476	0.034706
-0.68273	-0.26884	-0.2221	-0.98312	0.494997	-0.26231	0.131931	-0.3764	-0.26486
0.058332	0.036091	0.001244	-0.08031	-0.06813	0.015311	-0.10161	-0.01211	-0.11135
-0.23126	0.187807	0.017087	0.062228	-0.10512	0.166655	0.186951	-0.03102	0.119667
0.036363	0.599239	-0.77307	-0.44338	-0.47565	-0.18146	-0.07591	-0.2526	-0.13575
0.148605	-1.38772	0.066345	0.280897	1.151402	0.403568	-1.45342	0.40117	-0.5221
0.100581	0.028652	0.205986	0.304211	0.43834	0.087313	0.075293	-0.03635	0.262933
-0.13981	-0.87415	-0.62942	-1.97043	0.106555	-0.56205	-1.2788	1.48199	0.176405
-2.31488	0.570877	0.367229	-2.11803	0.71106	0.046994	0.381047	-0.27469	0.490573
-0.21444	1.579721	0.804587	1.036042	-2.68897	1.234547	-2.44708	2.415895	0.75098
0.441071	0.217128	-0.03751	0.917128	-0.54524	0.342851	-0.29512	-0.03972	0.153903
-0.12837	0.072263	0.051227	0.023129	-0.07073	0.125406	-0.01379	-0.06753	0.07886

-0.12708	0.191801	-0.94071	0.090674	0.0403	-0.04712	2.156617	1.361432	1.281029
-0.14302	-0.67359	0.086505	0.079603	-0.70008	-0.27361	0.232904	0.397298	0.099856
1.315457	1.07857	0.603432	2.961556	-0.80114	-0.20202	0.649391	-0.63619	0.412345
-0.08733	-0.31084	0.651594	-0.32479	-0.10719	0.021752	0.081463	0.149272	-0.38067
-0.05302	0.339802	-0.25635	-0.31944	-0.44191	0.257597	0.245912	0.026881	0.042522
0.041251	0.032322	-0.03296	-0.03307	-0.00761	-0.00887	0.008167	-0.00065	-0.03481
-0.2433	-0.46993	-0.11594	-1.04193	0.421343	0.030742	-0.36816	-0.05129	-0.09021
0.518108	-1.01322	-0.19055	-0.51033	0.086115	-1.08878	2.684461	-0.50748	-0.54134
1.719457	-1.25971	-0.7043	1.107755	0.995023	1.007302	-1.28399	-2.80643	0.179683
1.977595	1.04129	1.311881	-0.93918	1.505198	0.549458	0.274668	0.653158	0.87669
0.444783	0.367101	0.255838	-0.28254	-0.59677	-1.05553	-0.07618	-0.82385	-1.01
-1.38251	-0.13431	0.692802	-0.84693	0.381914	1.163188	-0.45566	-1.68058	-1.11667
0.246994	1.07194	-0.69692	-0.1424	-0.89788	1.335862	-0.24903	0.509866	0.157938
0.020402	0.971272	0.655747	-0.07049	-0.92274	-1.2468	2.31186	-0.57269	0.751988
1.775128	-0.1245	-0.75437	1.361294	1.151027	-0.9181	0.511774	0.847369	-0.87985
-0.16119	0.094713	0.24747	0.223049	0.08763	-0.03293	-0.07109	0.077601	-0.04666
0.083166	0.034146	-0.18497	-0.17474	0.002699	0.010461	0.163957	0.094191	-0.0787
0.020891	0.065037	-0.0258	0.009522	0.06475	0.034257	0.012453	-0.01691	0.009743
0.114061	-0.01668	0.013597	-0.10822	0.083456	-0.04715	0.131351	-0.18914	-0.00644
0.093161	-0.1245	0.271875	0.074871	0.080598	-0.08123	0.010017	-0.00514	0.003244
0.17365	-0.53023	0.067083	-0.12616	-0.2329	-0.13954	0.008839	-0.16609	-0.11671
-0.05278	-0.11813	0.050852	0.0538	0.056411	-4.34E-05	-0.12507	0.059379	0.00411
0.007567	0.709728	-0.14331	0.14314	0.182423	0.125208	-0.19642	-0.04851	0.070064
0.021273	-0.00778	0.048705	-0.03254	-0.02698	-0.01057	-0.03986	-0.02421	0.011133
0.883603	0.064995	-0.10583	0.625023	-0.52207	-1.17369	-0.05064	1.067283	0.075195
0.2499	-0.03696	0.099403	0.069695	0.066127	-0.04446	-0.15521	-0.0785	-0.20063
0.001594	0.00359	0.054561	0.000312	-0.00285	-0.00662	-0.07619	-0.0586	-0.04097
0.170176	-0.18523	0.060088	0.214606	0.063186	0.107652	-0.32431	0.145641	-0.09214
-0.68918	-0.9958	-0.53682	1.141672	0.387408	0.380774	0.832085	0.403272	0.28984
0.27348	-0.24274	0.018433	0.014961	-0.23746	-0.10099	-0.08627	-0.25502	0.05508
-0.07686	-0.24604	-0.65561	-0.6361	-2.74689	-0.12475	-0.84164	-1.50112	-1.131
0.119646	-0.5346	0.842553	-0.93443	-0.71115	0.222209	-0.34122	-0.5975	-0.07101
0.297857	0.009355	0.099182	-0.04436	0.313156	0.111677	-0.2964	-0.02394	-0.08467

PC72	PC73	PC74	PC75	PC76	PC77	PC78	PC79	PC80
0.183734	0.090141	0.065016	0.218206	-0.21799	-0.04178	-0.23054	-0.07519	0.391746
0.208916	-0.20679	-0.10205	0.027858	-0.42002	-0.54562	-0.34958	0.149371	0.757925
2.01045	-1.65157	-0.64508	-0.05717	-1.23569	0.125098	0.177219	0.324397	0.007806
0.143179	0.026757	0.03946	-0.13362	-0.03653	-0.09282	-0.1308	-0.00071	0.026015
0.034866	0.053159	0.026869	0.116059	-0.17243	-0.03642	-0.24471	0.004577	0.346767
0.080122	-0.98192	0.886302	-1.75949	3.961818	-0.725	0.597952	1.075499	-0.07384
0.041663	0.050851	0.043421	0.135508	-0.19271	-0.05916	-0.25911	0.003324	0.368223
0.095497	0.090514	0.047025	0.11967	-0.2069	-0.0638	-0.30034	-0.00824	0.370704
0.06415	0.047516	0.11074	0.149775	-0.1285	-0.08033	-0.26237	-0.12474	0.341008
0.20277	0.231487	0.064821	0.267154	-0.45692	-0.22257	-0.79139	0.507616	-3.22315
0.018182	0.186029	0.009377	0.235804	0.126846	0.040031	0.053731	0.08084	0.051001
-1.33788	0.514268	0.13871	0.330937	-0.352	-0.15734	-0.11213	0.182732	0.08506
-0.29296	0.825569	0.429585	-0.33741	0.307737	0.058353	-0.04849	-0.05154	-0.08544
0.024018	-0.63855	0.306381	-0.10045	1.022406	0.253297	-0.18198	-3.40842	-0.36891
0.438861	-0.47648	-0.09163	0.529439	0.216627	1.540242	-0.73972	0.60468	0.967321
-1.06387	-0.72089	-0.48092	-0.73355	-1.10639	0.491588	3.644248	-0.10961	-0.2976
-0.20239	-0.53166	-0.00496	-0.23471	0.015964	0.102613	-0.00074	0.113254	-0.04615
-1.78995	1.037891	0.648644	0.418532	0.504088	-0.39446	-0.06436	0.224097	-0.01773
0.233271	1.673313	-0.7357	0.094297	0.153047	-0.396	0.199535	0.217979	-0.02694
-0.021	0.031602	0.003213	0.007412	-0.02156	-0.00625	-0.00923	-0.01095	-0.0046
-0.02345	0.942306	-0.22038	0.51302	0.034957	0.211618	0.281758	0.284258	-0.02368
-0.57759	0.346351	0.049183	0.430149	-0.69219	-0.02552	-0.07986	0.108119	0.000969
0.363345	0.528013	0.137376	0.578055	0.276868	-0.33716	0.676339	-0.45568	0.039912
-0.10642	0.210253	-0.09477	0.148052	-0.44343	0.191944	-0.19055	0.759451	-0.0836
-0.27474	-1.14603	0.083191	-0.28989	0.556571	3.457614	-0.58456	0.19982	-0.31629
-1.50873	-1.81681	-0.49625	-2.0477	-1.04813	-1.90458	-1.27932	-0.21571	0.083911
1.853959	1.748439	0.687508	0.022263	0.006627	-0.63016	0.127638	-0.06422	0.02833
-0.52115	-0.78335	-0.37263	-0.1543	-0.3649	0.263547	0.037235	0.360827	0.058062
-0.16557	-0.00771	-0.02125	-0.02634	-0.19472	-1.34233	0.266398	-0.1269	0.269666
-0.21824	0.232521	-0.18445	0.294308	-0.07571	-0.1528	0.079132	0.086633	-0.00089
0.252975	-0.62723	-0.00631	-0.43679	0.03273	-0.09844	0.176011	0.095651	-0.17098
-0.5712	-0.98213	-0.1164	-0.0335	-0.0754	0.115715	-0.11226	-0.07877	-0.00735
0.247434	-0.1902	-0.19153	-0.10086	-0.0295	-0.75497	0.064455	0.435035	0.032825
0.35459	0.205826	-0.06751	-0.18346	0.098434	0.121452	-0.12797	-0.21078	-0.01919
0.462091	0.943826	0.083092	1.385703	0.62177	-0.05718	0.267952	-0.29881	0.006639
0.233213	0.94354	0.294126	0.053038	0.088826	-0.00791	0.010833	0.025772	0.033678
0.209153	0.239518	0.12749	0.141363	0.127829	0.228627	0.126331	-0.04654	-0.01057
0.361505	0.052931	0.084971	0.200479	0.133521	0.038667	-0.01377	0.019349	0.007395
-0.02921	-0.07425	0.002573	0.052436	0.070213	0.042278	0.008574	-0.02541	0.003526
0.883912	0.142541	-0.18496	0.240362	0.276642	-0.02841	0.054936	-0.08882	0.035224
0.038255	0.067158	0.036563	0.157633	-0.20424	-0.0994	-0.22396	0.002918	0.374883
-0.02471	0.040066	-0.02746	0.102368	0.059273	0.055218	0.023563	-0.00161	-0.00151
0.059093	0.10284	-0.07137	-0.11498	0.056888	-0.05724	-0.14349	0.041813	0.014827
-0.1303	-0.04697	-0.09815	0.06107	-0.1397	0.061499	-0.04977	-0.0427	0.003562
-0.09674	0.082226	-0.00441	0.150155	0.104756	0.190534	0.108014	-0.00681	-0.04296
-0.00827	-0.01086	-0.01621	-0.09021	-0.01235	0.009952	0.000671	0.064269	0.001304
-0.07143	0.178139	-0.19929	0.155822	0.084009	-0.04776	0.035631	0.051999	-0.0015
0.028194	0.10657	-0.09147	0.156135	-0.33872	0.039855	-0.03094	-0.11581	-0.01421
0.246009	0.005487	0.164546	0.249399	0.113546	-0.04171	-0.40711	0.005042	0.039854
-0.17922	0.156188	0.043192	0.078732	-0.04398	0.030454	-0.09271	-0.02035	0.047807
0.93453	-0.39095	-0.10119	-0.41235	0.21387	-0.24177	0.170311	-0.02909	-0.0419
0.090565	-0.23633	-0.14116	0.116553	-0.44926	0.084692	-0.06891	-0.03142	0.084525
0.888105	-0.35692	-1.73712	0.072834	0.286257	-0.20758	-0.01273	-0.00708	0.006156
-0.04798	0.678577	-2.49721	-0.91131	0.35237	0.101525	-0.07754	-0.14436	-0.09292
0.564894	-0.82787	1.260405	-0.24682	-0.22722	-0.1758	-0.06364	-0.13976	-5.75E-05
-0.09419	-0.30966	-0.24183	0.148287	0.081029	-0.00664	-0.02618	0.00019	-0.00867
0.019576	0.019988	0.00538	0.033579	0.033151	-0.00119	-0.00942	-0.00676	0.009957

2.087732	1.34571	1.934782	-2.90685	-1.29727	0.682691	0.011806	-0.10162	0.072157
-0.49879	0.375831	0.185661	0.062268	0.371568	-0.0059	-0.162	-0.09414	0.004884
-0.4322	0.391594	-1.75602	0.214952	0.57349	0.100803	-0.08978	-0.10228	-0.03642
-0.02345	-1.0699	0.349973	0.491984	0.048182	-0.11582	-0.00412	0.013354	0.013084
-0.08751	-0.14683	0.077473	-0.05692	-0.03499	0.086644	-0.02179	-0.00516	0.002875
0.01552	-0.03612	0.022097	-0.04801	0.022315	0.012574	-0.01107	-0.00741	0.000641
-0.23422	0.038974	0.190464	-0.19137	-0.22783	0.048807	-0.00437	0.058911	0.025667
1.232574	-1.49683	0.6719	1.369008	-0.22769	-0.43084	0.365564	0.187179	-0.09757
-1.71755	1.802808	0.007801	-1.41786	-0.34558	0.748483	-0.21411	-0.08738	0.152452
-0.30654	0.138967	0.227055	0.400181	0.079852	0.111758	0.10146	-0.20488	0.008286
0.15817	-0.4093	-0.06336	-0.36203	-0.05125	0.224583	-0.01718	-0.07995	-0.01308
-1.51523	-0.7584	2.987335	1.002675	-0.29379	-0.16534	0.078465	0.110761	0.004413
-0.92683	-0.58826	-0.20988	-0.17699	-0.01445	0.185911	-0.0577	0.024652	-0.01169
-1.23946	0.705295	0.2117	0.170216	-0.10752	0.305424	0.149621	0.118301	4.38E-05
0.280028	0.130512	0.539756	0.79964	0.223924	-0.37495	0.110676	0.113482	-0.02906
-0.03348	0.052345	-0.22973	0.464945	-0.50313	0.01882	-0.08108	-0.14	0.012887
-0.19496	-0.2193	-0.01439	-0.06873	-0.03741	0.052986	-0.0297	0.002611	-0.0015
-0.01033	-0.07588	0.008859	-0.02944	-0.00705	-0.01034	-0.01768	-0.01456	0.005075
0.037753	0.056876	-0.06289	0.091422	0.053762	-0.01719	-0.01266	0.002435	0.001687
-0.10973	-0.06607	-0.06662	0.002467	-0.11379	0.015321	0.021743	-0.02632	0.009387
0.129938	0.086529	0.018292	0.014	-0.04717	-0.04104	0.050559	0.002204	0.002033
-0.08593	-0.09282	0.0265	-0.03864	-0.00765	0.044909	-0.0054	-0.00937	0.004254
0.0085	0.054987	-0.03079	0.073523	0.200939	-0.01318	-0.11692	-0.03815	0.009386
-0.00465	-0.00786	0.011938	0.001129	0.005118	0.004577	0.001698	-0.00447	0.003628
0.622792	-0.46802	-0.83899	0.553708	0.287233	-0.11598	-0.0041	0.064295	-0.0052
-0.133	-0.08599	0.069991	0.015412	-0.02017	-0.04171	-0.00124	0.052158	0.001072
-0.0685	-0.05373	-0.05677	0.078464	0.042775	-0.01942	-0.00405	-0.00267	0.000411
0.015385	0.009169	0.03693	-0.02907	-0.01	0.025365	0.034656	0.004689	-0.017
-0.35823	-0.14213	-0.45514	0.329983	0.080412	0.081294	0.01701	0.077317	0.002985
0.146313	-0.04638	0.098841	0.06221	-0.2172	-0.04392	0.068808	0.027678	-0.00086
1.242676	0.344155	-0.00754	-0.25716	0.355917	0.02166	0.122838	-0.00625	-0.05229
0.082839	0.166639	-0.09863	0.012411	0.025329	-0.10555	0.054354	0.002849	-0.0127
-0.5935	0.246154	-0.42208	-0.41503	0.061542	-0.08774	-0.20259	-0.02098	0.02409

PC81	PC82	PC83	PC84	PC85	PC86	PC87	PC88	PC89
0.544015	-0.52773	-0.1418	-0.043	-1.33889	0.189235	0.011485	0.018545	0.002474
-0.60822	1.948576	-0.95228	0.728507	-0.08982	0.021028	-0.00076	-0.00019	-0.00112
0.182488	-0.10662	0.20866	-0.14414	0.03777	-0.00487	-0.00029	-0.00033	-5.27E-05
-0.03169	0.03689	0.008733	0.01609	2.63E-05	0.004192	0.002583	0.002483	0.000549
0.371966	-0.32936	-0.12694	-0.00117	0.238771	-0.28624	0.291319	-0.04886	-0.02375
0.111479	0.121353	0.10919	-0.02947	-0.02424	0.00317	-0.00199	-0.00031	-0.00034
0.386568	-0.30799	-0.11583	-0.04465	0.267967	-0.25413	-0.03989	0.049049	0.126393
0.413043	-0.35757	-0.0955	-0.01081	0.132755	-0.24857	-0.16299	-0.21267	-0.03252
0.427853	-0.28808	-0.06573	-0.04254	0.546694	0.90972	0.006614	-0.00528	-0.00243
-0.5797	0.13041	0.071579	-0.01303	-0.0392	0.015304	0.000244	-2.57E-05	-0.0001
-0.03382	0.012305	0.016035	-0.00482	0.012092	6.26E-05	-8.96E-05	0.000372	5.08E-05
-0.02462	0.09732	0.107256	0.019681	-0.00031	0.007583	0.00033	0.001234	0.00017
0.025885	0.057111	0.12293	0.029535	0.029143	-0.01309	-0.00477	-0.00049	0.000469
0.115532	0.518914	0.395664	-0.30839	-0.02478	-0.03908	0.000718	-0.00021	-0.00045
-1.79267	0.062217	1.337278	-0.2546	-0.05913	0.013248	0.002457	-0.00117	-9.00E-05
-0.28531	0.068944	0.177474	0.044552	-0.01788	0.013019	-0.00046	-0.00278	2.23E-05
-0.01546	-0.02802	-0.03187	0.014318	0.005783	0.003126	0.00055	-0.00032	-0.00011
0.000894	0.009159	-0.16351	0.085449	-0.01563	-0.00939	-0.00029	-0.00022	3.27E-05
-0.06232	-0.02419	0.015208	0.016751	-0.00683	-0.00953	-0.00051	-0.00068	-0.0003
0.013811	-0.00209	0.004677	0.005791	0.001807	-0.00039	4.87E-05	-0.00011	4.71E-07
0.109132	0.05564	-0.00215	0.04949	0.005352	-0.0032	-0.00056	-3.87E-05	-0.00014
0.138294	-0.0084	-0.05651	0.02873	0.003562	-0.00665	-0.00018	-0.00085	4.04E-05
-0.2568	-0.44253	0.35965	1.214254	0.012433	-0.00639	-0.00342	0.005237	-0.00051
1.520649	1.159633	0.9741	-0.63658	-0.02852	-0.01004	0.00231	0.001397	-0.0012
0.12664	-0.25224	-0.99342	0.184258	0.019898	0.00363	-0.0028	0.000937	-0.00034
-0.26202	-0.42395	0.320982	0.579521	-0.00421	0.001383	-0.00042	0.00387	-0.00033
-0.22892	-0.18841	-0.0408	0.171102	0.003452	0.002423	0.001022	0.00025	1.59E-05
-0.09003	-0.06867	-0.14531	0.079252	-0.01447	-0.00189	-0.00275	-0.00034	8.22E-05
-1.06316	-0.18361	-0.9087	-1.5601	-0.01104	0.016638	0.003895	-0.01154	0.002127
0.053042	0.040835	0.017607	-0.0226	-0.00607	-0.00105	-0.00035	0.000159	-2.61E-05
0.266278	-0.0426	-0.22693	0.014866	0.001898	-0.01417	-0.00109	-0.00215	-0.00047
0.092456	0.077145	0.038309	-0.08948	-0.00637	0.005644	-0.00023	0.000427	-5.69E-05
0.135364	-0.04092	0.220025	-0.02697	0.005297	-0.00683	0.001443	7.85E-05	9.76E-05
-0.14714	-0.06086	-0.07216	0.034094	-0.00674	0.004961	0.002036	-0.00018	0.000959
-0.23653	-0.11393	-0.08745	0.22029	0.043481	0.00575	-0.00107	0.000617	0.000171
0.007609	0.032731	0.079151	-0.01237	-0.0061	-0.00248	0.000359	-7.27E-05	-3.72E-05
0.057745	0.024764	0.003616	0.009328	0.004993	0.000573	8.42E-05	0.000523	-3.06E-05
0.00932	-0.00249	-0.00999	0.0276	-0.00022	-0.00362	0.000117	-0.00036	0.000128
-0.00139	-0.00554	0.001435	-0.01574	0.002652	-0.00041	-3.36E-05	-3.06E-05	5.96E-06
-0.09044	-0.05759	0.036115	-0.01276	0.001563	0.001917	-0.00011	-3.90E-05	9.54E-05
0.327559	-0.33576	-0.15415	-0.12846	0.270807	-0.26453	-0.09292	0.200102	-0.07153
0.008358	-0.00207	0.005285	-0.00592	0.000932	-0.00019	2.68E-05	9.84E-06	2.30E-05
0.01346	-0.02129	-0.00797	-5.13E-05	0.003275	-0.00256	0.0002	0.000172	1.83E-05
-0.0021	0.010461	-0.00903	-0.01231	0.001473	-0.00165	1.66E-05	-0.00011	2.90E-05
0.048772	-0.03532	0.015352	-0.01133	0.002429	-0.00038	0.000185	0.00073	2.37E-05
0.01693	-0.01842	0.011723	0.015344	0.000683	0.000691	0.000178	-0.00017	5.20E-05
-0.00576	-0.01246	-0.00271	0.001454	0.003056	0.001921	0.000259	0.000246	1.01E-05
0.01736	-0.02706	-0.02657	0.003888	0.003271	-0.00173	0.00028	0.000334	5.92E-05
0.03715	-0.00887	-0.02549	0.003691	0.003662	-0.00117	0.00018	0.000204	4.70E-05
-0.05733	0.003628	0.043198	-0.00134	0.004174	0.001959	0.000384	0.000214	0.000219
-0.05737	-0.00809	-0.01987	0.009808	-0.00263	0.002577	-0.00018	0.00069	-0.0002
-0.01211	0.064089	-0.00546	0.014935	-0.00707	0.011113	-0.01196	0.001194	0.000803
0.002339	-0.03579	-0.0006	0.008183	0.007689	-0.00071	-0.00059	7.69E-05	6.95E-05
0.119219	-0.01704	-0.14013	0.021322	0.005134	0.012364	-0.0004	0.000979	8.26E-05
-0.03479	-0.04243	0.013858	0.050683	-0.00555	-0.00156	-0.0005	-6.94E-05	4.26E-05
0.008037	-0.00658	0.026272	-0.00342	-0.00591	-0.00771	-0.00035	-0.00038	6.41E-05
-0.0117	0.000499	0.004807	0.004585	0.006042	-0.00059	-3.24E-05	-7.94E-05	-2.78E-05

0.022296	0.107068	-0.02021	-0.05634	0.006971	-0.00396	0.000928	0.000938	-6.36E-06
0.060796	-0.23456	0.062498	-0.0637	0.005558	-0.00162	0.000294	0.00023	0.000133
0.011913	0.015224	-0.00701	0.009477	-0.00738	0.004123	-0.00014	-0.00052	-6.35E-07
-0.01649	-0.04913	-0.00255	0.010774	0.006382	-0.00116	0.000115	7.25E-05	5.00E-05
0.012762	0.001839	-0.0121	0.003497	0.002302	0.000456	5.26E-05	-1.00E-05	-5.58E-05
-0.00321	-0.0032	0.000588	0.005109	0.001229	-0.00014	5.19E-05	-6.12E-05	2.38E-05
-0.00334	0.002798	-0.00261	-0.01476	0.002019	0.000154	-0.00013	0.000249	-2.85E-05
0.168071	0.014093	-0.08504	0.063544	0.018707	-0.00813	0.000286	-0.00072	-0.00021
-0.21991	0.008397	0.147942	-0.0843	-0.00864	0.007816	-0.0003	0.000474	-0.00015
-0.03462	0.020479	-0.05754	-0.0223	0.006011	0.00244	-0.0017	-0.00283	-0.0002
0.008576	0.007053	0.02346	-0.03642	0.003856	-0.00404	0.000562	0.000522	-2.06E-05
-0.05258	-0.04337	0.031634	0.015607	-0.00472	-0.00075	0.000295	3.27E-05	-6.52E-05
0.083393	0.067196	0.018244	-0.05764	-0.00258	-0.00052	-0.00053	-0.00025	-0.00011
0.069532	-0.00091	0.024767	0.000556	-0.00231	-0.00144	-8.15E-05	-0.00142	0.000136
0.088743	0.057327	-0.00784	-0.02809	-0.00045	-0.01422	0.000504	-0.00015	-3.27E-05
-0.02612	-0.01813	-0.01239	0.017004	0.002718	-0.0062	0.000469	9.24E-05	0.000175
0.022844	0.016563	-0.01602	-0.01319	0.000199	0.000133	-8.79E-05	-0.00013	-3.82E-05
-0.00566	-0.00591	-0.00125	-8.04E-05	0.001145	-0.00018	4.19E-05	3.19E-05	8.99E-06
0.014975	0.005376	-0.00264	0.003741	0.001851	-0.00188	0.000486	-1.60E-05	-4.95E-05
-0.01061	-0.02939	-0.00741	0.019806	0.002922	-0.00127	0.000188	0.000269	3.76E-05
-0.01701	-0.03212	-0.016	0.014961	0.004185	-0.00046	0.000596	0.000229	1.73E-05
-0.00445	0.000523	-0.00403	0.000722	-0.00026	0.001355	-0.0011	0.000145	8.54E-05
0.017713	0.03855	-0.01131	-0.02768	-0.0012	0.000679	-3.02E-05	-0.00051	-6.94E-05
0.003757	-0.00496	-0.0045	0.001863	0.001267	-0.00045	1.68E-05	3.22E-05	1.18E-05
0.067173	0.02629	-0.02781	0.018186	0.002286	-0.0037	0.000197	-0.0015	0.000211
0.002032	-0.01274	-0.01592	0.007143	0.001698	0.000612	0.000103	3.27E-05	-1.31E-05
-0.00321	-0.00725	-0.00112	0.005781	0.00036	-0.00027	-2.12E-05	-2.98E-05	1.39E-07
0.039199	0.005971	-0.0217	-0.01788	0.000826	-0.00017	-0.00013	0.000121	-2.60E-05
0.010759	0.057139	0.006276	-0.06554	-5.35E-05	0.000536	0.000349	-0.00094	-0.00029
0.064661	-0.00206	-0.02048	0.009204	0.002417	-0.0035	0.000247	-0.00038	-7.67E-06
-0.04843	-0.0854	-0.09344	0.001721	-0.00285	0.003809	0.0006	0.005588	0.001009
0.007203	-0.00742	-0.00161	0.007513	-0.00841	-0.01844	0.000373	0.000567	6.88E-05
-0.04861	-0.01339	0.019808	0.000438	-0.00449	0.001921	0.000141	-0.0002	0.000148

PC90
1.07E-14
6.04E-15
-4.95E-15
-7.51E-15
2.16E-14
-6.51E-15
2.44E-14
2.60E-14
-2.66E-15
3.12E-14
-2.62E-16
-1.22E-14
-9.50E-16
-2.82E-15
1.38E-14
3.24E-15
1.50E-14
9.33E-15
-1.83E-15
-1.75E-14
8.34E-15
-1.14E-14
-1.82E-14
-2.14E-14
-6.12E-15
2.17E-14
-4.87E-15
-1.56E-14
-2.17E-15
2.54E-15
-1.05E-14
2.21E-14
-2.01E-14
4.72E-15
-2.30E-14
-7.73E-15
2.05E-14
2.62E-14
-3.78E-14
1.04E-14
2.51E-14
-2.04E-14
-3.59E-16
2.01E-14
1.50E-14
-9.11E-15
-9.33E-15
-3.30E-15
7.98E-17
1.85E-14
-2.98E-14
5.72E-16
-3.20E-15
3.95E-15
1.50E-14
4.77E-15
-1.99E-14

-1.89E-14
3.51E-15
3.38E-15
1.33E-15
-2.23E-14
-4.35E-14
2.61E-14
-3.11E-15
-9.63E-15
-1.17E-14
1.31E-14
-3.13E-15
1.09E-15
-5.48E-15
7.96E-15
2.61E-14
-8.28E-15
1.22E-15
9.83E-16
-2.61E-14
1.04E-14
-1.94E-15
-1.78E-14
-4.34E-16
-2.70E-14
-1.14E-14
-1.71E-15
-4.46E-15
-6.07E-15
2.82E-14
2.27E-14
2.34E-15
1.72E-14

Supplementary Table 3. Fungus in the tissue samples showing differential abundance between the GC and control group at the family level

Family	cancer	control	diff	conf_min	conf_max	p-value	q-value
Saccharomycetales_fam_Incertae_sedis	32.58898	12.19214	20.39684	8.362741	32.43094	0.001223	0.081323
Pseudeurotiaceae	0.081833	0.965331	-0.883498	-1.402346	-0.36465	0.001289	0.081323
Pleosporaceae	3.458229	1.245167	2.213062	0.860167	3.565957	0.001718	0.081323
Trimorphomycetaceae	0.081256	0.960929	-0.879673	-1.429693	-0.329653	0.002324	0.082515
Chaetomiaceae	1.900364	4.887093	-2.986729	-5.110553	-0.862904	0.006529	0.16264
Aspergillaceae	6.433913	11.91026	-5.476344	-9.403601	-1.549088	0.006872	0.16264
Mortierellaceae	0.025218	0.362407	-0.337189	-0.593524	-0.080854	0.011088	0.224937
Chrysozymaceae	0	0.26282	-0.26282	-0.473882	-0.051758	0.015841	0.263532
Cordycipitaceae	0.016749	0.758787	-0.742038	-1.355817	-0.128258	0.018944	0.263532
Nectriaceae	9.677444	3.555973	6.121471	0.965999	11.27694	0.020948	0.263532
Phaffomycetaceae	0.00124	0.949818	-0.948578	-1.751867	-0.145288	0.021718	0.263532
Lasiosphaeriaceae	0.186153	1.027258	-0.841104	-1.557749	-0.12446	0.02227	0.263532
Clavicipitaceae	0.058104	0.563471	-0.505367	-0.962409	-0.048324	0.030922	0.337769
Morosphaeriaceae	4.44E-05	0.169696	-0.169651	-0.326507	-0.012795	0.034669	0.351643
Sporocadaceae	0.00056	0.806322	-0.805762	-1.573096	-0.038429	0.040012	0.356175
Thermoascaceae	0.587491	1.774704	-1.187213	-2.319003	-0.055423	0.040132	0.356175
Thelephoraceae	0.027749	0.133049	-0.1053	-0.207088	-0.003512	0.042884	0.358211

Supplementary Table 4. Fungus in the tissue samples showing differential abundance between the GC and control group at the genus level

Genus	cancer	control	diff	conf_min	conf_max	p-value	q-value
Candida	32.31241	8.845929	23.46648	11.41324	35.51972	0.000246	0.053792
Saitozyma	0.081256	0.960929	-0.879673	-1.429693	-0.329653	0.002324	0.248931
Alternaria	3.073987	1.147318	1.926669	0.658463	3.194875	0.00341	0.248931
Thermomyces	0	1.427949	-1.427949	-2.483482	-0.372416	0.009158	0.467747
Mortierella	0.025218	0.362407	-0.337189	-0.593524	-0.080854	0.011088	0.467747
Slooffia	0	0.260064	-0.260064	-0.471368	-0.048761	0.017019	0.467747
Aspergillus	2.72042	6.234584	-3.514164	-6.430996	-0.597333	0.018911	0.467747
Wickerhamomyces	4.67E-05	0.948851	-0.948804	-1.752198	-0.145411	0.021704	0.467747
Starmerella	0	2.554493	-2.554493	-4.725891	-0.383095	0.022188	0.467747
Fusarium	7.938322	2.486869	5.451453	0.692908	10.21	0.025626	0.467747
Pseudogymnoascus	0.068787	0.448038	-0.379251	-0.724041	-0.034461	0.031791	0.467747
Acrocalymma	4.44E-05	0.169696	-0.169651	-0.326507	-0.012795	0.034669	0.467747
Trichosporon	0.008071	0.312749	-0.304678	-0.593737	-0.015619	0.039302	0.467747
Pestalotiopsis	0.000456	0.806322	-0.805867	-1.5732	-0.038533	0.039988	0.467747
Thermoascus	0.573911	1.761318	-1.187407	-2.320932	-0.053881	0.040396	0.467747

Supplementary Table 5. Fungus in the tissue samples showing differential abundance between the GC and control group at the species level by Welch's t test

Species	cancer	control	diff	conf_min	conf_max	p-value	q-value
Candida_albicans	31.00224	4.365933	26.63631	15.49378	37.77884	1.50E-05	0.003053
Aspergillus_montevidensis	0.375822	2.819969	-2.444147	-3.895849	-0.992445	0.001437	0.146536
Saitozyma_podzolica	0.081256	0.960929	-0.879673	-1.429693	-0.329653	0.002324	0.158057
Arcopilus_cupreus	0	0.978202	-0.978202	-1.635864	-0.320541	0.004461	0.227524
Aspergillus_sydowii	0.032807	1.076218	-1.043411	-1.765042	-0.32178	0.005579	0.227611
Penicillium_arenicola	0.102258	1.081398	-0.97914	-1.681585	-0.276695	0.00722	0.245493
Fusicolla_acetilerea	0.625127	0.082896	0.542231	0.101547	0.982916	0.01691	0.375449
Slooffia_tsugae	0	0.260064	-0.260064	-0.471368	-0.048761	0.017019	0.375449
Mortierella_elongata	0.005307	0.304602	-0.299296	-0.542558	-0.056033	0.017054	0.375449
Fusarium_solani	0.539087	0.050389	0.488698	0.086235	0.891161	0.018404	0.375449
Wickerhamomyces_anomalus	4.67E-05	0.948851	-0.948804	-1.752198	-0.145411	0.021704	0.377191
Starmerella_bacillaris	0	2.554493	-2.554493	-4.725891	-0.383095	0.022188	0.377191
Metarhizium_carneum	0	0.284871	-0.284871	-0.553249	-0.016493	0.038004	0.454641

Supplementary Table 6. Fungus in the tissue samples showing differential abundance between the GC and control group at the species level by Wilcoxon rank sum test					
**Species**	**cancer**	**control**	**fold(control/cancer)**	**p-value**	**q-value**
Metarhizium_carneum	0	0.284871	Inf	5.73E-05	0.003384
Leohumicola_minima	4.67E-05	0.127556	2733.3333	6.69E-05	0.003384
Candida_albicans	31.00224	4.365933	0.1408264	7.17E-05	0.003384
Ustilaginoidea_virens	0	0.099047	Inf	0.000116	0.003384
Starmerella_bacillaris	0	2.554493	Inf	0.000116	0.003384
Arcopilus_cupreus	0	0.978202	Inf	0.000116	0.003384
Slooffia_tsugae	0	0.260064	Inf	0.000116	0.003384
Eremothecium_coryli	0.065224	6.89E-05	0.0010562	0.00017	0.004109
Pyrenochaetopsis_leptospo	0.001276	0.015884	12.452962	0.000192	0.004109
Wickerhamomyces_anomalus	4.67E-05	0.948851	20332.524	0.000201	0.004109
Thermoascus_crustaceus	2.89E-05	0.309667	10719.231	0.000414	0.00671
Arxiella_dolichandrae	2.22E-05	0.108124	4865.6	0.000414	0.00671
Leptospora_rubella	0	0.022058	Inf	0.000459	0.00671
Trichomonascus_ciferrii	0.001469	0.906513	617.14221	0.000467	0.00671
Aspergillus_sydowii	0.032807	1.076218	32.80485	0.000505	0.00671
Millerozyma_miso	0.00018	0.061676	342.64198	0.000526	0.00671
Aspergillus_montevidensis	0.375822	2.819969	7.503465	0.000586	0.007032
Humicola_grisea	0.02846	0.035291	1.240025	0.000782	0.008861
Botryosphaeria_dothidea	0.610576	1.026767	1.6816374	0.001661	0.016913
Yueomyces_sinensis	0	0.029478	Inf	0.001741	0.016913
Myrmecridium_schulzeri	0	0.062582	Inf	0.001741	0.016913
Aspergillus_terreus	0.095147	0.019336	0.2032184	0.002779	0.02476
Candida_intermedia	0.002236	0.755153	337.79225	0.002792	0.02476
Candida_tropicalis	0.540693	0.722724	1.3366624	0.002984	0.025256
Colletotrichum_gloeosporioides	0.331907	0.177164	0.5337779	0.0033	0.025256
Saitozyma_podzolica	0.081256	0.960929	11.826008	0.003302	0.025256
Mycoleptodiscus_indicus	0	0.040089	Inf	0.003343	0.025256
Corynascella_humicola	0	0.018307	Inf	0.00637	0.046411
Magnaporthe_grisea	0.016007	0.000838	0.0523393	0.007669	0.053949
Gibellulopsis_piscis	0.000684	0.025009	36.538961	0.010509	0.069572
Rhizopus_arrhizus	0.061758	0.300853	4.8715052	0.010572	0.069572
Corticifraga_peltigerae	0	0.029898	Inf	0.012071	0.07462
Chaetosphaeria_vermicularioides	0	0.009456	Inf	0.012071	0.07462
Umbelopsis_dimorpha	0.000813	0.031836	39.142077	0.012813	0.076877
Malassezia_restricta	0.045702	0.391218	8.5601478	0.013454	0.077219
Auricularia_reticulata	1.570738	3.056567	1.9459433	0.013627	0.077219
Candida_glabrata	0.758738	2.857498	3.7661203	0.014443	0.079632
Metarhizium_anisopliae	0.055142	0.079813	1.4474087	0.015334	0.082319
Exophiala_lecanii-corni	0.011204	0.002151	0.1919873	0.01698	0.08882
Exserohilum_rostratum	0	0.005736	Inf	0.0228	0.108156
Boothiomyces_macroporosum	0	0.004922	Inf	0.0228	0.108156
Hygrocybe_acutoconica	0	0.010576	Inf	0.022803	0.108156
Hexagonia_apiaria	0	0.004047	Inf	0.022803	0.108156
Penicillium_arenicola	0.102258	1.081398	10.575213	0.023328	0.108156
Fusicolla_aquaeductuum	0.116338	0.048202	0.4143299	0.026626	0.120704
Zopfiella_marina	0.000158	0.009342	59.211268	0.027323	0.121138
Malbranchea_cinnamomea	0.00786	0.000542	0.068985	0.027909	0.121138
Issatchenkia_orientalis	0.000507	0.011298	22.298246	0.030001	0.127503
Hannaella_coprosmae	0.003942	0.056531	14.33991	0.037839	0.15142

Arcopilus_aureus	0.291596	0.011447	0.0392553	0.040759	0.15142
Oidiodendron_maius	0.01812	0.020607	1.1372333	0.04214	0.15142
Podospora_longicollis	0	0.24096	Inf	0.043111	0.15142
Suillus_bovinus	0	0.094273	Inf	0.043111	0.15142
Phellinus_noxius	0	0.055182	Inf	0.043111	0.15142
Fibrodontia_alba	0	0.049853	Inf	0.043111	0.15142
Coniochaeta_fasciculata	0	0.043856	Inf	0.043111	0.15142
Robbauera_albescens	0	0.011538	Inf	0.043111	0.15142
Metarhizium_marquandii	0	0.005969	Inf	0.043111	0.15142
Sphaerulina_chaenomelis	0.002258	0.09456	41.88189	0.043793	0.15142

Species	Ca-1	Ca-2	Ca-3	Ca-4	Ca-5	Ca-6
Candida_albicans	18954	82715	22364	4647	94277	49122
Penicillium_chermesinum	41	1293	5229	14	115	84
Auricularia_reticulata	11	3	8	18	4	3
Candida_glabrata	16	506	1344	43	3474	6154
Aspergillus_montevidensis	2	2	8	2	15	54
Starmerella_bacillaris	0	0	0	0	0	0
Thermoascus_aurantiacus	4	2	4	5	23	40
Cladosporium_halotolerans	4	672	861	0	29	1
Botryosphaeria_dothidea	0	2	2	3	126	15
Candida_tropicalis	10	4130	6889	253	2	7
Paraboeremia_selaginellae	5	1	453	0	1	1
Penicillium_arenicola	0	0	0	0	0	0
Aspergillus_sydowii	0	0	0	0	2	1
Diutina_catenulata	0	0	0	0	0	0
Saitozyma_podzolica	4	0	0	1	3	6
Arcopilus_cupreus	0	0	0	0	0	0
Dipodascus_australiensis	13	1	0	1042	0	0
Wickerhamomyces_anomalus	0	0	0	0	0	0
Trichomonascus_ciferrii	0	0	0	0	0	0
Candida_intermedia	0	0	0	0	0	0
Nigrospora_oryzae	0	0	0	0	0	0
Fusicolla_acetilerea	0	0	0	0	0	0
Sarocladium_zeae	0	0	0	0	0	1
Chaetomium_angustispirale	4	0	0	0	115	2
Aureobasidium_pullulans	10	1	2	0	0	0
Cordyceps_polyarthra	0	0	0	0	2	0
Staphylotrichum_boninense	0	0	0	0	2	3783
Fusarium_solani	0	3	4	0	0	0
Talaromyces_neofusisporus	19	0	0	0	0	1
Aspergillus_chlamydosporus	1	0	0	0	0	0
Aspergillus_penicillioides	43	2	1471	0	0	1
Colletotrichum_gloeosporioides	0	0	0	3	3	4
Monochaetia_dimorphospora	0	0	0	0	0	0
Oidiodendron_chlamydosporicum	0	0	4	0	0	3
Malassezia_restricta	1	0	0	0	0	8
Rasamsonia_composticola	11	3	1	0	0	12
Hannaella_oryzae	0	0	0	1	2	2
Rhizopus_arrhizus	0	0	0	0	0	0
Sordaria_fimicola	7	0	5	0	0	5
Mortierella_elongata	0	3	1	2	8	1
Thermoascus_crustaceus	0	0	0	0	0	0
Periconia_byssoides	2	0	0	0	1	3
Arcopilus_aureus	0	6	9	2	1	1
Cutaneotrichosporon_dermatis	4	1	0	0	3	0
Metarhizium_carneum	0	0	0	0	0	0
Hannaella_luteola	0	0	0	0	2	1
Clavispora_lusitaniae	0	0	0	0	0	0
Penicillium_brevicompactum	0	0	0	0	0	0
Chrysosporium_pseudomerdarium	0	0	1	0	0	0
Slooffia_tsugae	0	0	0	0	0	0
Podospora_longicollis	0	0	0	0	0	0
Sarocladium_strictum	0	6	4660	0	2	3
Plectosphaerella_cucumerina	0	0	0	0	0	0
Verticillium_leptobactrum	23	1	0	2	0	0
Penicillium_cryptum	0	0	0	0	0	0
Fusicolla_violacea	0	0	0	0	0	0
Papiliotrema_flavescens	0	1033	2	3	0	0

**Acremonium_polychromum**	0	0	0	0	0	0
**Schizopora_flavipora**	0	0	0	0	7	0
**Fusicolla_aquaeductuum**	56	0	0	11	0	0
**Filobasidium_magnum**	10	67	0	0	0	0
**Penicillium_pimiteouiense**	3	0	0	0	0	0
**Zasmidium_musae**	0	0	0	0	0	1
**Coniochaeta_polymorpha**	0	0	0	2	0	0
**Blumeria_graminis**	0	0	0	0	0	1
**Metarhizium_anisopliae**	0	0	0	0	0	0
**Leohumicola_minima**	0	0	0	0	0	0
**Schizothecium_carpinicola**	0	0	0	0	0	0
**Exophiala_mesophila**	0	0	0	0	0	0
**Aspergillus_terreus**	0	0	0	0	0	0
**Rhodotorula_mucilaginosa**	0	0	0	0	1	0
**Apiosordaria_jamaicensis**	4	2	0	0	0	2
**Naganishia_sp**	0	0	0	0	0	0
**Arxiella_dolichandrae**	0	0	0	0	0	0
**Wallemia_canadensis**	3	0	0	0	0	0
**Brycekendrickomyces_acaciae**	0	0	0	0	0	0
**Deconica_phyllogena**	0	0	0	0	0	0
**Candida_apicola**	0	0	0	0	0	0
**Ustilaginoidea_virens**	0	0	0	0	0	0
**Naganishia_albida**	0	0	0	0	0	0
**Sphaerulina_chaenomelis**	1	0	0	0	0	0
**Suillus_bovinus**	0	0	0	0	0	0
**Myrothecium_cinctum**	0	0	0	0	0	0
**Curvularia_verruculosa**	0	0	0	0	0	0
**Setophoma_terrestris**	0	0	0	0	0	5
**Cryptococcus_uniguttulatus**	0	0	0	0	0	0
**Engyodontium_album**	0	0	0	0	0	0
**Acremonium_hyalinulum**	0	0	0	0	0	0
**Microdochium_colombiense**	0	0	0	0	0	0
**Hannaella_zeae**	0	0	0	0	0	0
**Papiliotrema_aurea**	0	0	0	0	0	0
**Apiotrichum_laibachii**	3	5	4	0	0	0
**Gongronella_butleri**	0	0	2383	0	0	0
**Zasmidium_syzygii**	0	0	0	0	0	0
**Rigidoporus_vinctus**	0	0	2	0	0	0
**Eremothecium_coryli**	330	0	452	0	36	0
**Humicola_grisea**	0	0	0	0	0	0
**Myrmecridium_schulzeri**	0	0	0	0	0	0
**Pleurotus_ostreatus**	0	0	0	0	0	0
**Millerozyma_miso**	0	0	0	0	0	0
**Hannaella_coprosmae**	0	3	4	0	0	0
**Sporobolomyces_symmetricus**	0	0	0	0	0	0
**Cutaneotrichosporon_curvatus**	0	0	0	0	0	0
**Periconia_epilithographicola**	16	0	0	0	0	0
**Phellinus_noxius**	0	0	0	0	0	0
**Trichothecium_roseum**	0	0	0	0	0	0
**Exophiala_oligosperma**	0	0	0	0	0	1
**Fibrodontia_alba**	0	0	0	0	0	0
**Russula_cascadensis**	0	0	0	0	0	0
**Kazachstania_humilis**	0	0	0	0	0	0
**Kazachstania_heterogenica**	0	0	0	0	0	0
**Periconia_echinochloae**	0	0	0	0	0	0
**Pseudallescheria_boydii**	0	0	0	0	0	0
**Heterochaete_delicata**	0	0	0	0	0	0
**Coniochaeta_fasciculata**	0	0	0	0	0	0

**Dactylonectria_estremocensis**	0	0	0	0	0	0
**Umbelopsis_isabellina**	0	0	0	0	0	0
**Mycoleptodiscus_indicus**	0	0	0	0	0	0
**Oidiodendron_maius**	3	4	0	0	0	0
**Clitopilus_hobsonii**	0	0	0	0	0	0
**Zasmidium_xenoparkii**	0	0	0	0	0	0
**Exophiala_jeanselmei**	0	0	0	0	0	0
**Malassezia_globosa**	0	0	22	0	0	1
**Pochonia_bulbillosa**	0	0	0	0	0	0
**Umbelopsis_dimorpha**	0	0	0	0	0	0
**Tolypocladium_album**	1	0	4	0	0	0
**Cystobasidium_lysinophilum**	0	0	0	0	0	0
**Agrocybe_pediades**	0	0	0	0	0	2
**Corticifraga_peltigerae**	0	0	0	0	0	0
**Cladosporium_sphaerospermum**	0	0	0	0	0	0
**Yueomyces_sinensis**	0	0	0	0	0	0
**Sarcopodium_circinosetiferum**	0	0	0	0	0	0
**Golubevia_pallescens**	0	0	0	0	0	0
**Gliomastix_tumulicola**	0	0	0	0	0	0
**Penicillium_glabrum**	2	0	0	0	0	0
**Udeniomyces_pyricola**	0	0	0	0	0	0
**Gibellulopsis_piscis**	0	0	0	0	0	0
**Cutaneotrichosporon_debeurmannianum**	0	0	0	0	0	1
**Erythrobasidium_hasegawianum**	0	2	1003	0	0	0
**Leptospora_rubella**	0	0	0	0	0	0
**Kwoniella_bestiolae**	0	0	0	0	0	0
**Mariannaea_samuelsii**	0	0	0	0	227	0
**Kondoa_sorbi**	0	0	0	0	0	0
**Exobasidium_kishianum**	0	0	0	0	0	0
**Mortierella_alpina**	0	0	0	1	0	0
**Corynascella_humicola**	0	0	0	0	0	0
**Aplosporella_javeedii**	0	0	0	0	0	0
**Pyrenochaetopsis_leptospora**	0	0	0	0	0	0
**Magnaporthe_grisea**	0	0	0	0	2	1
**Monascus_purpureus**	0	0	0	0	0	1
**Byssochlamys_laguncularDiae**	0	1	0	0	0	0
**Exophiala_alcalophila**	0	0	0	0	0	0
**Polyschema_sclerotigenum**	0	0	0	0	0	0
**Knufia_epidermidis**	0	0	0	0	0	0
**Verruconis_gallopava**	0	0	0	0	0	0
**Exophiala_lecanii-corni**	0	0	0	0	0	0
**Dekkera_custersiana**	0	1	3	0	0	0
**Symmetrospora_coprosmae**	0	0	0	0	0	0
**Wallemia_tropicalis**	0	0	0	0	0	0
**Apiotrichum_xylopini**	0	0	0	1	1	2
**Mortierella_zonata**	0	0	0	0	0	0
**Issatchenkia_orientalis**	0	0	0	0	0	0
**Robbauera_albescens**	0	0	0	0	0	0
**Byssochlamys_spectabilis**	0	0	0	0	0	0
**Tomentella_papuae**	0	0	0	0	0	0
**Gibberella_tricincta**	0	0	0	0	0	0
**Penicillium_menonorum**	0	0	1	0	0	0
**Hygrocybe_acutoconica**	0	0	0	0	0	0
**Westerdykella_dispersa**	2	0	0	0	0	1
**Penicillium_jiangxiense**	0	0	0	0	0	0
**Zopfiella_marina**	0	0	0	0	0	0
**Chaetosphaeria_vermicularioides**	0	0	0	0	0	0

**Spizellomyces_dolichospermus**	0	0	0	0	0	0
**Wilcoxina_mikolae**	0	0	0	26	0	0
**Kodamaea_ohmeri**	0	0	0	0	0	0
**Eutypella_citricola**	0	0	0	0	0	0
**Orbilia_stipitata**	0	0	0	0	0	0
**Malbranchea_cinnamomea**	0	0	0	0	0	0
**Umbelopsis_changbaiensis**	6	0	0	2	0	0
**Sagenomella_keratitidis**	0	0	0	0	0	0
**Hydnochaete_japonica**	0	0	0	0	0	0
**Cystobasidium_slooffiae**	0	0	0	0	0	0
**Acremonium_fusidioides**	0	0	0	0	0	0
**Acidomelania_panicicola**	0	0	0	0	0	0
**Preussia_globosa**	0	1	0	0	0	0
**Uwebraunia_dekkeri**	0	0	0	0	0	0
**Metarhizium_marquandii**	0	0	0	0	0	0
**Latorua_caligans**	0	0	0	0	0	0
**Exserohilum_rostratum**	0	0	0	0	0	0
**Sympodiomycopsis_paphiopedili**	0	0	0	0	0	0
**Penicillium_sublateritium**	0	0	0	0	0	0
**Colletotrichum_chlorophyti**	1	0	0	0	0	0
**Hannaella_sinensis**	0	0	0	0	0	0
**Boothiomyces_macroporosum**	0	0	0	0	0	0
**Meliniomyces_bicolor**	0	0	0	0	0	0
**Vishniacozyma_victoriae**	0	0	0	0	0	0
**Tomentella_coerulea**	0	0	0	0	0	0
**Dimorphospora_foliicola**	0	0	0	0	0	0
**Hexagonia_apiaria**	0	0	0	0	0	0
**Hortaea_werneckii**	0	0	0	0	0	0
**Glutinoglossum_glutinosum**	0	1	0	0	0	0
**Aspergillus_baarnensis**	0	0	0	1	0	0
**Peniophora_incarnata**	0	0	0	0	0	0
**Wallemia_sebi**	0	0	0	0	0	0
**Hannaella_phetchabunensis**	0	0	0	0	0	0
**Myxocephala_albida**	1	0	0	0	0	0
**Tomentella_stuposa**	0	0	0	0	0	0
**Ophiocordyceps_sinensis**	0	0	0	0	0	0
**Dictyosporium_heptasporum**	0	0	0	0	0	0
**Oberwinklerozyma_silvestris**	0	0	0	0	0	0
**Rhizosphaera_oudemansii**	0	0	0	0	0	0
**Veronaeopsis_simplex**	0	4	3	0	0	0
**Candida_fructus**	0	0	0	0	0	0
**Tausonia_pullulans**	0	0	0	0	0	0
**Simplicillium_aogashimaense**	0	1	1	0	0	0
**Candida_haemulonis**	0	0	0	0	0	0
**Clavaria_citrinorubra**	0	0	0	0	0	0
**Suillus_grevillei**	1	0	0	0	0	0
**Cyberlindnera_fabianii**	0	0	0	0	0	0
**Cladorrhinum_bulbillosum**	0	0	0	0	0	0
**Hannaella_kunmingensis**	0	0	0	0	0	0
**Glomus_indicum**	0	1	0	0	0	0
**Rhodotorula_diobovata**	0	0	0	0	0	0
**Yamadazyma_triangularis**	0	0	0	0	0	0
**Clavatospora_longibrachiata**	0	0	0	0	0	0
**Mortierella_horticola**	0	0	0	0	0	0
**Aspergillus_halophilicus**	0	0	0	0	0	0
**Coniochaeta_cateniformis**	2	0	0	0	0	0
**Myrothecium_inundatum**	0	0	0	0	0	0
**Aphanoascus_terreus**	0	0	0	0	0	0

**Dioszegia_zsoltii_var_zsoltii**	0	0	0	0	0	1
**Schizopora_ovispora**	0	0	0	0	0	0
**Clavaria_falcata**	0	0	0	0	0	0
**Bifiguratus_adelaidae**	0	0	0	0	0	0
**Curvularia_intermedia**	0	0	0	0	0	0
**Earliella_scabrosa**	0	0	0	0	0	0
**Kazachstania_pintolopesii**	0	0	0	0	0	0
**Scytalidium_lignicola**	6	0	0	0	0	0
**Leptodontidium_trabinellum**	0	0	0	0	0	0
**Stachybotrys_chartarum**	0	0	0	0	0	0
**Myrmecridium_thailandicum**	0	0	0	0	0	0
**Mortierella_parvispora**	0	0	0	0	0	0
**Malassezia_arunalokei**	0	0	0	0	0	0
**Oidiodendron_setiferum**	0	0	0	0	0	0
**Buckleyzyma_aurantiaca**	0	0	0	0	0	0
**Torula_hollandica**	0	0	0	0	0	0
**Mortierella_chienii**	0	0	0	0	0	0
**Phialemoniopsis_cornearis**	1	0	5	0	0	0
**Daldinia_starbaeckii**	0	0	0	0	0	0
**Tetracladium_marchalianum**	0	0	0	0	0	0
**Colletotrichum_crassipes**	0	0	0	0	1	0
**Phialocephala_humicola**	0	0	0	0	0	0
**Trichocladium_opacum**	0	0	0	0	0	0
**Piloderma_bicolor**	0	0	0	0	0	0
**Coniochaeta_hoffmannii**	0	0	2	0	0	0
**Jaminaea_angkorensis**	0	0	0	0	0	0
**Debaryomyces_hansenii**	0	1	0	0	0	0
**Talaromyces_proteolyticus**	0	0	0	0	0	0
**Phaeoacremonium_rubrigenum**	0	0	0	0	0	0
**Bulleromyces_albus**	0	0	0	1	0	0
**Talaromyces_aerugineus**	0	0	0	0	0	0
**Acremonium_persicinum**	0	0	0	0	0	0
**Xenoacremonium_recifei**	0	0	0	0	0	0
**Oculimacula_yallundae**	0	0	0	0	0	0
**Marasmiellus_candidus**	0	0	0	0	0	0
**Penicillium_paxilli**	0	4	0	0	0	0
**Sarocladium_kiliense**	0	0	0	0	0	0
**Westerdykella_reniformis**	0	0	0	0	0	0
**Moesziomyces_aphidis**	0	0	0	0	0	0
**Trametes_versicolor**	0	0	0	0	0	0
**Corallomycetella_repens**	0	0	0	0	0	0
**Schizophyllum_commune**	0	0	0	0	0	0
**Poaceascoma_helicoides**	1	0	0	0	0	0
**Wilcoxina_rehmii**	0	0	0	0	0	0
**Cyphellophora_gamsii**	0	0	0	0	0	0
**Thanatephorus_cucumeris**	0	0	0	0	0	0
**Humicola_phialophoroides**	0	0	0	0	0	0
**Fluminicola_thailandensis**	0	0	0	0	0	0
**Preussia_terricola**	0	0	0	0	0	0
**Hypholoma_myosotis**	0	0	0	0	0	0
**Rhodosporidiobolus_odoratus**	0	0	0	0	0	0
**Symmetrospora_vermiculata**	0	0	0	0	0	0
**Sterigmatomyces_halophilus**	0	0	0	0	0	0
**Waitea_circinata**	0	0	0	0	0	0
**Leucocoprinus_cepistipes**	0	0	0	0	0	0
**Leucocoprinus_birnbaumii**	0	0	0	0	0	0
**Myrothecium_gramineum**	0	0	0	0	0	0
**Gymnopilus_punctifolius**	0	0	0	0	0	0

**Schizothecium_inaequale**	0	0	0	0	0	0
**Pyrenula_aspistea**	0	0	0	0	0	0
**Neopestalotiopsis_asiatica**	0	0	0	0	0	0
**Suillus_viscidus**	0	0	0	0	0	0
**Leucogyrophana_mollusca**	0	0	0	0	0	0
**Acremonium_charticola**	0	0	0	0	0	0
**Dentocorticium_sulphurellum**	0	0	0	0	0	0
**Pseudotomentella_tristis**	0	0	0	0	0	0
**Meira_argovae**	0	0	0	0	0	0
**Preussia_flanaganii**	0	0	0	0	0	0
**Micropsalliota_furfuracea**	0	0	0	0	0	0
**Hanseniaspora_opuntiae**	0	0	0	0	0	0
**Pseudozyma_pruni**	0	0	0	0	0	0
**Unclassified**	975	14377	45464	9169	1164	49492

Ca-7	Ca-8	Ca-9	Ca-10	Ca-11	Ca-12	Ca-13	Ca-14	Ca-15
100767	105238	95853	93422	24808	108	63683	2857	71917
51	64	947	0	5	4	2129	228	6855
110	0	0	0	3603	4	5	2	0
10	11	197	1999	0	1	3314	86	107
14	11	11	1	15	3	3	49	2728
0	0	0	0	0	0	0	0	0
5	3	506	39	37	8	1	72	415
0	0	5	171	3770	1	3	2	0
12	7	3	33	0	2	0	5	17
0	6	12	798	5	7	1976	9	2
1	90	0	0	1287	2	1	1	87
3	2	5	0	1	0	0	0	3
2	2	0	0	0	1	0	0	1204
0	0	0	0	0	0	0	0	0
14	1	5	0	0	1	0	1	5
0	0	0	0	0	0	0	0	0
0	0	1	0	4	0	2237	2	0
0	0	0	0	0	0	0	0	0
0	0	0	0	0	0	0	0	0
0	0	2	0	0	0	1	0	0
1	5	0	0	0	0	0	1	1
0	0	0	2	0	11	0	1	1
0	0	0	0	0	0	0	2	0
7	405	0	0	2	3808	0	1	17
3	3	2	0	6	3	801	0	273
0	1	19	0	2	0	0	4	2
0	0	178	1	0	0	1	6	0
0	0	0	3	3	1	0	0	1
0	0	0	0	0	0	0	2	4
0	0	0	0	3	0	1	1	0
4	3	2	0	6	1878	0	0	141
2	2	1	0	0	0	0	0	1806
0	0	0	0	0	0	0	0	0
2	1	0	2	0	0	0	0	0
5	0	19	11	3	0	0	3	1
0	0	1	0	10	2	0	9	328
0	0	0	0	5296	0	0	0	2
2	0	0	0	1	0	0	22	0
0	1	1	0	3	9	0	2	3
0	0	6	1	0	0	0	3	0
0	0	0	0	0	0	0	0	0
0	3	0	0	0	569	0	0	0
0	6	0	0	0	0	1	1	0
3	0	0	0	1	0	0	0	0
0	0	0	0	0	0	0	0	0
0	10	0	0	2171	0	0	7	0
0	0	0	0	1	0	0	0	0
0	0	0	0	0	0	0	0	0
7	0	9	0	1	1	0	1	0
0	0	0	0	0	0	0	0	0
0	0	0	0	0	0	0	0	0
0	0	0	0	0	0	0	1	0
0	0	0	0	0	0	0	0	0
1	0	0	0	0	0	3240	0	0
0	3	0	0	0	0	0	0	0
0	0	0	0	0	0	0	1	0
0	36	0	0	0	0	0	0	0

0	0	0	0	0	0	0	0	0
0	0	0	0	0	0	0	3	0
0	0	324	1	7	0	4394	20	0
0	1	0	0	1	0	0	0	149
18	1	5	1	1	53	2	2	14
0	0	0	0	0	0	0	0	0
0	0	0	0	0	0	0	0	0
0	0	0	0	0	0	0	0	0
0	0	0	0	2300	0	0	0	0
0	0	0	0	0	1	0	0	0
0	0	0	0	0	1	0	0	0
0	0	0	0	0	0	0	0	0
0	0	0	0	0	0	0	0	0
1	0	16	0	0	0	0	2	0
1	0	0	0	2	0	0	2	0
0	1	0	0	0	0	0	0	0
0	0	0	0	0	0	0	0	0
0	0	0	0	188	1	6	0	1
6	0	7	0	0	0	0	1	0
0	0	0	0	0	0	0	0	0
0	0	0	0	0	0	0	0	0
0	0	0	0	0	0	0	0	0
0	0	0	0	0	0	0	0	0
0	0	0	0	0	0	1	3	0
0	0	0	0	0	0	0	0	0
0	0	0	0	0	0	0	0	0
0	0	0	0	0	0	0	0	0
0	0	0	0	0	0	0	0	0
0	0	0	0	0	0	0	0	0
0	0	0	0	0	0	0	0	0
0	0	0	0	0	0	0	0	0
0	0	0	0	0	0	0	1	0
0	0	0	0	8	0	0	0	0
0	0	185	0	0	0	0	0	0
0	0	2	0	0	1	272	0	0
0	0	0	0	0	1	0	0	0
0	0	0	0	0	0	0	0	0
0	0	0	0	0	0	0	0	0
0	157	0	0	0	0	0	0	0
0	0	0	0	545	0	1	0	1
0	0	0	0	0	0	0	0	0
0	0	0	0	0	0	0	0	0
0	0	0	0	0	0	0	0	0
0	0	0	0	0	0	0	0	0
0	0	0	0	0	0	0	0	0
0	0	1	0	1085	0	0	0	0
0	0	0	2	0	0	2	0	0
0	0	0	0	0	0	0	0	0
0	0	0	0	0	0	0	0	0
0	0	0	0	0	0	0	0	0
0	0	0	0	0	0	0	0	0
0	0	0	0	0	0	0	1	0
0	0	96	0	0	0	0	0	0
0	0	7	0	9	0	0	0	0
0	0	0	0	0	0	0	0	0
0	0	0	0	0	1	0	0	0
0	0	0	0	0	0	0	0	0
0	0	0	0	0	0	0	0	0

0	0	0	0	1048	0	0	0	0
6	0	0	0	0	0	0	0	0
0	0	0	0	0	0	0	0	0
5	0	0	0	2	0	0	1	2
0	0	0	0	0	0	0	0	0
0	0	2	0	0	0	0	0	1557
0	0	0	0	0	0	0	0	1
0	0	0	70	0	0	0	1	0
0	0	0	1	0	0	0	0	0
0	0	0	1	0	0	0	0	1
0	0	0	0	757	6	2	0	1
0	0	25	0	0	0	0	0	0
0	0	0	0	0	0	0	0	0
0	0	0	0	0	0	0	0	0
0	0	0	0	0	0	0	0	0
0	0	0	0	0	0	0	0	0
2	0	0	0	0	0	0	0	0
0	0	0	0	0	0	0	0	0
0	0	0	0	0	0	0	0	0
0	0	0	0	2	0	0	0	0
0	0	0	0	0	0	0	2	0
0	4	0	0	0	0	0	0	0
0	0	0	0	0	0	0	1	0
0	0	0	0	0	0	0	0	0
0	0	0	0	0	0	0	0	0
0	0	0	0	0	0	0	0	0
0	0	0	0	21	0	0	0	0
0	0	0	0	0	0	0	0	0
0	0	0	0	764	0	0	0	0
0	0	0	0	0	1	1	0	0
0	0	0	0	0	0	0	0	0
0	0	0	0	0	0	0	0	0
0	0	0	0	0	0	0	0	0
0	0	0	0	0	0	0	2	0
0	0	0	0	1	0	0	2	0
0	0	0	0	0	0	0	1	0
0	1	0	0	0	0	0	0	0
0	0	0	0	1	0	0	0	1
0	0	0	0	0	0	0	0	0
0	0	0	0	0	0	0	0	0
0	0	0	0	0	0	0	0	0
0	0	0	0	0	0	0	13	0
0	0	0	0	0	0	0	0	0
0	0	0	0	0	0	0	0	0
2	1	9	0	1	1	0	0	0
0	0	3	0	0	0	0	0	0
0	0	0	0	0	0	0	1	0
0	0	0	0	0	0	0	0	0
0	0	0	0	0	0	0	0	0
0	0	0	0	0	0	0	1	0
0	0	0	0	0	0	0	0	0
4	1	1	0	0	2	0	0	4
0	0	0	0	0	0	0	0	0
0	1	0	0	5	0	0	2	0
0	0	0	0	0	0	0	1	0
0	0	0	0	0	0	0	0	0
0	0	0	0	0	0	0	0	0

0	0	0	0	0	0	0	0	0
0	0	0	0	0	0	0	0	0
0	0	0	0	0	0	0	0	0
0	0	0	0	0	0	0	0	0
0	0	0	0	1	0	0	0	0
0	0	0	1	0	0	0	0	0
0	0	2	1	0	2	0	0	0
0	0	0	0	0	0	0	0	0
0	0	0	0	0	0	0	0	0
0	0	0	0	0	0	0	0	0
0	0	0	0	0	0	0	0	0
0	0	0	0	0	0	0	0	0
1	0	0	0	0	0	0	0	0
0	0	0	0	0	0	0	0	0
0	0	0	0	0	0	0	0	0
13	0	0	0	0	0	0	0	0
0	0	0	0	0	0	0	0	0
0	0	0	0	0	0	0	0	0
0	0	0	0	0	0	0	0	0
0	0	0	0	0	0	0	0	0
0	0	0	0	0	0	0	0	0
0	0	0	0	0	0	0	0	0
0	0	0	0	0	0	0	0	0
0	0	0	0	0	0	0	0	0
0	0	1	0	3	0	0	3	0
0	0	0	0	0	0	0	0	0
0	0	0	0	0	0	0	0	0
0	0	0	0	0	0	0	0	0
0	0	0	0	0	0	0	0	0
0	0	0	0	0	0	0	0	0
0	0	0	0	0	0	0	0	0
0	0	0	0	0	0	0	0	158
0	107	0	0	30	0	0	0	0
0	0	0	0	1	0	0	0	0
0	0	0	0	0	0	0	0	0
0	0	0	0	0	0	0	0	0
3	2	1	0	0	0	0	0	2
0	0	0	0	0	0	0	0	0
0	0	0	0	0	0	0	0	0
0	0	0	0	0	0	0	0	0
0	0	0	0	0	0	0	0	0
0	46	0	0	0	0	0	0	0
0	0	65	0	0	0	0	0	0
0	0	0	0	0	0	0	0	0
0	0	0	0	0	0	0	0	0
0	0	0	0	1	1	1	0	0
0	3	0	0	0	0	0	1	0
0	0	0	0	0	0	0	4	0
0	0	0	0	0	0	0	0	0
0	0	0	0	0	0	0	0	0
0	0	11	0	0	0	0	0	0
0	0	0	0	0	0	0	0	0
0	0	0	0	0	0	0	0	0
0	1	0	0	0	0	0	0	0
0	0	0	0	0	0	0	0	0
0	0	0	0	0	0	0	0	0
0	0	0	0	1	1	0	0	0
0	0	0	0	0	0	0	0	0

6	0	0	0	1	0	0	2	0
0	0	0	0	0	0	0	0	0
0	0	0	0	0	0	0	0	0
0	0	0	0	0	0	0	0	0
0	0	0	0	0	0	0	0	0
0	0	0	0	0	0	0	0	0
0	0	0	0	0	0	0	0	0
0	0	0	0	0	0	0	0	0
0	2	0	0	0	0	0	0	0
0	0	0	0	0	0	0	0	0
0	0	0	0	0	0	0	0	0
0	0	0	0	0	0	0	0	0
0	1	0	0	0	0	0	0	2
0	0	0	0	0	0	0	0	0
0	0	0	0	0	0	0	0	0
0	0	0	0	0	0	0	0	0
0	0	0	0	0	0	0	0	0
0	0	0	0	0	2	0	0	3
0	0	0	0	0	0	0	0	0
0	0	0	0	0	0	0	0	0
0	0	0	0	0	0	0	0	0
0	0	0	0	0	0	0	0	0
0	0	0	0	0	0	0	0	0
0	0	0	0	0	0	0	0	0
0	0	0	0	0	0	0	0	0
0	0	0	0	0	0	0	0	1
0	0	8	0	0	0	0	0	0
0	0	0	0	0	0	0	0	0
0	0	0	0	0	0	0	0	2
0	2	0	0	0	0	0	0	0
0	0	1	0	0	0	0	0	0
0	0	0	0	1	0	0	0	0
0	0	0	0	0	0	0	0	0
0	0	0	0	8	0	0	0	0
0	0	0	0	0	0	0	0	0
0	0	0	0	10	0	0	0	0
0	0	0	0	0	0	0	0	0
0	0	0	0	0	0	0	0	0
0	0	0	0	0	0	0	0	0
0	0	0	0	0	0	0	0	0
0	0	0	0	0	0	0	0	0
0	0	0	0	3	0	0	0	0
0	0	0	0	0	0	0	0	0
0	1	0	0	0	0	1	0	0
0	0	0	0	0	0	0	0	0
0	0	0	0	0	0	0	0	0
0	0	1	0	0	0	0	0	0
0	0	7	0	0	0	0	0	0
0	0	0	0	0	0	0	0	0
1	0	0	0	0	0	0	0	0
0	0	0	0	0	0	0	0	0
0	0	0	0	0	0	0	0	0
0	0	0	0	0	0	0	0	0
0	0	0	0	0	0	0	0	0
0	0	0	0	0	0	0	0	0
0	0	0	0	0	0	0	0	0
0	0	0	0	0	0	0	0	0
0	0	0	0	0	0	0	0	0

0	0	0	0	0	0	0	0	0
0	0	0	0	0	0	0	0	0
0	0	0	0	0	0	0	0	0
0	0	0	0	0	0	0	0	0
0	0	0	0	0	0	0	0	0
0	0	0	0	0	0	0	0	0
1	0	0	0	0	0	0	0	0
0	0	0	0	0	0	1	0	0
0	0	0	0	0	0	0	0	0
0	0	0	0	0	0	0	0	0
0	0	0	0	0	0	0	0	0
0	2	0	0	0	0	0	0	0
0	0	0	0	0	0	0	0	0
335	1290	5021	8328	45015	87159	22100	2598	11884

Ca-16	Ca-17	Ca-18	Ca-19	Ca-20	Ca-21	Ca-22	Ca-23	Ca-24
27900	47323	31210	63285	481	641	2851	46	50
679	4727	11934	1563	20	74	16267	117	1383
13	16	11	11	2	8684	1	9	16
764	351	6952	2615	0	124	103	0	0
3	7	2947	1	51	8	64	5	11
0	0	0	0	0	0	0	0	0
12	9	17	20	51	11	87	4	4
0	0	2	4	13	17	1	4	26
29	3917	754	1	3	4	4	321	1385
510	4	2	11	3	916	16	25	0
2	1	0	51	0	0	0	20	2
6	4	5	0	0	0	0	5	0
6	1	36	0	0	0	3	39	46
1	127	0	0	3	0	0	0	0
2	3	2260	1	16	1	16	0	0
0	0	0	0	0	0	0	0	0
1	5	23	5	25	7	0	0	26
0	0	0	0	0	0	0	0	0
0	0	0	0	1	0	3	0	0
0	0	0	2	1	0	1	0	0
1	0	0	0	4	0	0	0	0
0	0	0	0	4	1	0	3533	5922
1244	4	1	0	0	0	0	0	0
7	10	628	4	0	8	0	47	7
13	12	12	0	0	1	0	68	0
0	0	0	4	0	64	1	0	0
2	0	6	0	3	0	4	1	8
0	0	0	0	0	4473	0	1524	1931
0	1	0	0	0	0	1	6	2
0	0	0	0	2	0	0	0	0
8	6	1387	0	5	4	6	2	1
4	5	9	596	6	1	4	47	647
0	0	0	0	0	0	0	0	0
4	0	0	0	0	0	2	0	0
7	181	6	2	0	523	1	1	1
11	3632	28	7	1	0	642	1	0
0	1072	3	1	0	5	10	0	0
0	0	0	0	0	0	2	0	0
5	0	5	1	5	0	4	2	16
0	0	0	0	0	2	0	1	0
0	0	0	0	0	0	1	0	0
0	0	0	0	0	0	3413	0	2
0	0	0	0	0	9	4	0	0
0	0	0	0	1	2	0	1	1
0	0	0	0	0	0	0	0	0
4	4	4	0	0	4	3	0	0
0	0	4	0	0	0	0	0	13
0	0	0	0	3	0	0	0	0
0	0	0	2	0	2	0	0	2
0	0	0	0	0	0	0	0	0
0	0	0	0	0	0	0	0	0
3	3	3140	0	0	8	1	0	0
1	0	0	0	0	0	0	1799	16
0	0	0	0	0	1	1	0	0
15	16	17	0	0	0	0	0	0
0	0	0	0	1	0	0	0	0
0	1	0	0	11	5	0	1	0

0	0	0	0	0	0	1	0	0
0	0	0	0	0	0	5677	0	0
0	0	0	0	0	0	0	0	0
0	0	0	0	3	0	0	8	1
5	0	0	2	1	4	2	0	33
0	0	0	0	0	0	0	0	4
1	0	0	0	0	0	0	0	0
0	0	0	0	0	0	0	0	0
0	0	0	0	0	5	0	0	0
0	0	0	0	0	0	0	0	0
0	0	0	0	0	0	0	0	0
0	0	0	0	0	0	0	0	0
0	0	0	0	0	0	0	0	0
0	0	0	0	1	0	2103	0	0
3	3	4111	0	1	1	1	2	0
0	0	0	0	0	0	0	0	0
0	0	0	0	0	0	0	0	0
28	0	0	0	0	0	0	0	1
0	0	0	1665	0	0	0	0	0
0	0	0	0	0	0	0	0	0
0	0	0	0	0	0	0	0	0
0	0	0	0	0	0	0	0	0
0	0	0	0	0	0	0	1	0
0	1	0	7	0	0	0	0	0
0	0	0	0	0	0	0	0	0
0	0	0	0	0	0	0	0	0
0	0	0	0	0	1	0	0	0
0	0	9	4	0	0	0	0	0
0	0	0	0	0	0	0	0	1
0	0	0	0	0	0	0	0	3
0	0	0	0	0	0	0	0	0
0	0	0	0	0	0	0	0	0
0	0	0	0	0	0	0	0	0
0	0	0	0	0	0	0	0	2
0	0	0	0	0	7	0	0	0
0	0	0	0	0	0	0	1	0
0	0	1	0	0	0	0	0	0
1	0	0	0	0	0	0	0	0
0	0	0	0	0	1	1	7	0
2	4	4	0	0	0	0	0	0
0	0	0	0	0	0	0	0	0
0	0	0	0	0	0	0	0	0
0	0	0	0	0	0	0	0	0
0	0	0	0	0	1	0	1	68
0	0	0	0	0	0	0	0	0
137	0	0	679	0	1	0	0	0
0	0	0	0	0	0	0	0	0
0	0	0	0	0	0	0	0	0
0	0	0	0	0	0	0	0	0
0	0	6	3	0	0	0	0	0
0	0	0	0	0	0	0	0	0
1	122	0	0	0	0	0	0	0
0	0	0	0	0	0	0	0	0
0	0	0	2012	0	0	0	0	0
0	0	0	0	0	0	0	0	0
0	0	0	0	0	0	0	2	0
0	0	0	0	0	0	0	0	0
0	0	0	0	0	0	0	0	0

1	0	0	0	0	0	0	0	0
0	0	0	1	0	0	0	0	0
0	0	0	0	0	0	0	0	0
0	0	0	0	11	1	9	1	0
0	0	0	0	0	0	0	0	0
0	0	0	0	0	0	0	0	0
0	0	0	0	0	0	0	0	0
0	0	0	0	0	0	1	0	0
0	0	0	0	0	0	0	3	0
0	1	0	0	0	0	1	0	1
0	0	0	0	0	1	0	2	2
0	0	0	0	0	0	2	0	0
2	3	1106	0	0	0	0	0	0
0	0	0	0	0	0	0	0	0
0	0	0	0	0	0	0	0	0
0	0	0	0	0	0	0	0	0
1	0	2	0	0	0	0	0	0
0	0	0	0	0	0	0	0	0
0	0	0	0	0	0	0	0	1
0	0	0	0	0	1	0	0	0
0	1	0	0	0	0	0	0	0
0	4	0	0	0	0	0	0	0
0	0	1	2	0	0	0	0	0
0	0	0	0	0	1	0	0	0
0	0	0	0	0	0	0	0	0
0	0	0	0	0	0	0	0	0
0	0	0	0	0	0	0	7	0
0	0	0	0	0	0	0	0	1
0	0	0	0	0	2	0	0	0
0	0	0	0	0	0	0	0	0
0	0	0	0	0	0	0	0	0
0	0	0	0	0	0	0	0	0
0	0	0	0	0	0	0	0	0
0	0	0	7	0	0	3	0	0
0	0	0	0	0	0	0	0	0
0	0	0	0	0	0	0	0	0
0	0	0	0	0	0	0	0	0
0	0	0	0	0	1	0	0	0
0	0	0	0	0	0	0	0	0
0	0	0	0	0	0	0	0	0
0	0	3	0	0	0	0	0	45
4	0	0	0	5	3	0	0	0
0	0	0	0	0	0	0	0	0
0	0	0	0	0	0	0	0	0
0	0	0	0	0	0	2	0	0
0	0	0	0	6	0	1	0	0
0	0	0	0	0	0	0	0	0
0	0	0	0	0	0	0	0	0
0	0	0	0	0	0	0	0	60
0	0	0	0	4	0	2	0	0
0	0	0	0	0	0	0	0	56
0	0	2	0	3	6	1	2	2
0	0	0	0	0	0	0	0	0
0	1	0	0	2	1	6	0	1
0	1	0	0	0	0	0	0	0
0	0	0	0	0	0	0	0	0
0	0	0	0	0	0	0	0	0

0	0	0	0	0	0	0	0	0
0	0	0	0	0	0	0	0	0
0	0	0	0	4	0	0	0	0
0	0	0	0	0	0	0	0	0
0	0	0	0	0	397	0	0	0
0	0	0	0	6	0	0	0	0
2	1	0	2	0	0	0	0	7
0	0	0	0	0	0	0	1	0
0	0	0	0	0	0	0	0	0
0	0	0	0	1	0	0	0	0
0	0	0	0	0	0	0	0	0
0	0	0	0	0	0	0	0	0
0	0	0	0	0	0	0	0	0
0	0	0	0	0	0	0	0	0
0	0	0	0	0	0	0	0	0
0	0	0	0	0	0	0	0	0
0	0	0	0	0	0	0	0	0
0	0	0	0	0	0	0	0	0
0	0	0	0	0	0	0	6	0
0	0	0	0	0	0	0	0	0
0	0	0	0	0	0	0	1	0
0	0	0	0	0	0	0	0	0
0	0	0	0	0	0	0	0	0
0	0	0	0	0	0	1	0	0
0	0	0	0	1	0	0	0	0
0	0	0	0	0	0	0	0	0
0	0	0	0	0	0	0	0	0
0	0	0	0	0	0	0	0	0
0	0	0	0	0	0	0	0	0
0	0	0	0	0	0	3	0	0
0	0	0	0	0	0	0	0	0
0	0	0	0	0	0	0	0	0
0	0	0	0	0	0	0	0	0
1	0	0	1	0	0	0	0	0
0	0	0	0	2	0	0	0	0
0	0	1	0	0	0	0	0	112
5	0	0	0	0	0	0	0	0
0	0	0	0	0	0	0	0	0
0	0	0	0	0	0	0	0	0
0	0	0	0	0	6	0	0	0
0	0	0	0	2	0	0	0	0
0	0	0	0	0	0	0	20	0
0	0	0	1	0	2	0	0	0
0	0	0	0	0	0	0	0	0
0	0	0	0	0	0	0	0	0
0	0	0	0	0	0	0	0	0
0	0	0	0	0	0	0	0	0
0	0	0	0	0	0	0	0	0
0	0	0	0	0	0	0	0	0
0	0	0	0	0	0	0	0	0
0	0	0	0	0	0	0	0	0
0	0	0	0	0	0	4	0	0
0	0	0	0	0	0	0	0	0
0	4	0	0	0	0	0	0	0
0	0	0	0	0	0	0	0	0
0	0	0	0	0	0	0	0	0
0	0	0	0	0	0	0	0	0
2	5	6	0	0	0	0	0	0

0	0	0	0	0	0	0	0	0
0	0	0	0	0	0	0	0	0
0	0	0	0	0	0	0	0	0
0	0	0	0	1	0	0	0	0
0	0	0	0	1	0	0	0	0
0	0	0	0	0	0	0	0	0
0	0	0	1	0	0	0	0	0
0	0	0	0	0	0	1	0	0
0	0	0	0	0	0	0	0	0
0	0	0	0	0	0	0	43	0
0	0	0	0	0	0	0	0	0
0	0	0	0	0	0	0	0	34
0	0	0	0	0	0	3	0	0
0	0	0	0	0	0	0	0	0
0	0	0	0	0	0	0	0	0
0	0	0	0	0	0	0	28	0
0	0	0	0	0	0	0	0	0
0	0	0	0	0	0	0	0	0
0	0	0	0	0	0	0	0	0
0	0	0	0	0	0	0	0	0
1	0	1	0	0	0	0	0	0
0	0	0	0	0	0	0	0	0
0	0	0	0	0	1	0	0	0
0	0	0	0	0	0	0	0	0
0	0	0	0	0	0	0	0	0
0	0	0	0	0	0	0	0	0
0	0	1	0	0	0	0	0	0
0	0	0	0	0	0	0	2	0
0	0	0	0	0	0	0	0	0
0	0	0	0	0	0	0	0	0
0	0	0	0	0	0	0	0	0
0	0	0	0	0	0	0	0	0
0	0	0	0	0	0	0	5	0
0	0	0	0	0	0	0	0	0
0	0	0	0	0	0	0	0	0
0	0	0	0	0	0	0	0	0
0	2	0	0	0	0	0	0	0
0	0	0	0	0	0	1	0	0
0	0	0	0	0	0	0	0	0
0	0	0	0	0	0	0	0	0
0	0	0	0	0	0	0	0	0
0	0	0	0	0	0	0	0	0
0	0	0	0	0	0	0	0	0
0	0	0	0	0	0	0	0	0
0	0	0	0	0	0	0	0	0
0	0	0	0	0	0	2	0	0
0	0	0	1	0	0	0	0	0
0	0	0	0	0	0	0	0	0
0	0	0	0	0	0	0	0	0
0	0	0	0	0	0	0	0	0
0	0	0	0	0	0	0	0	0
0	0	0	0	0	0	0	0	0
0	0	0	0	0	0	0	0	0
0	0	0	0	0	0	0	0	0
0	0	0	0	0	0	0	0	0
0	0	0	0	0	0	0	0	0
0	0	0	0	0	0	0	0	0
0	0	0	0	0	0	0	0	0

0	0	0	0	0	0	0	0	0
0	0	0	0	0	0	0	0	0
0	0	0	0	0	0	0	0	0
0	0	0	3	0	0	0	0	0
0	0	0	0	0	0	0	0	0
0	0	0	0	0	0	0	0	0
0	0	0	0	0	0	0	0	0
0	0	0	1	0	0	0	0	0
0	0	0	0	0	0	2	0	0
0	0	0	0	0	0	0	0	0
0	0	0	0	0	0	0	0	0
0	0	0	0	0	0	0	0	0
0	0	0	0	0	0	0	0	0
5958	21437	20954	24747	1050	89408	45362	89026	83896

Ca-25	Ca-26	Ca-27	Ca-28	Ca-29	Ca-30	Ca-31	Ca-32	Ca-33
518	219	93	359	409	18	3828	19058	11
219	1637	6	50	4	8	13697	865	21
629	3	32368	58	35	1	7	25221	1
3	9	0	2	3	4	52	21	9
16	18	7	2514	8	65	56	5	19
0	0	0	0	0	0	0	0	0
801	1159	48	6593	30	7	94	1848	880
33	2084	3	4	1	74	43	0	24
252	2538	10	3298	15	16	28	10	2450
5	16	0	6	0	4	887	7	64
276	1872	0	526	0	53	5	2	115
1	0	0	0	0	0	0	3	0
0	0	0	0	0	1	5	0	4
0	0	0	0	0	331	0	0	0
1	3	0	1	0	0	1	0	1
0	0	0	0	0	0	0	0	0
11	8	0	0	967	23	0	1	7
0	0	0	0	0	0	0	0	0
0	0	0	0	0	0	0	0	0
0	0	0	0	0	1	0	0	0
1	0	3	127	1	1	0	0	0
3369	2048	3402	1002	3764	0	3	0	23
0	0	0	0	0	0	0	0	0
2	7	0	0	0	0	8	5109	1023
23	1314	0	0	0	0	1	9	11
0	0	4	3	2	7	0	1	1
2	1	2	0	0	0	0	0	1
292	3870	8	13	2706	0	0	0	29
19	15	0	2	0	0	3456	1	21
1	0	0	0	2	0	1	0	0
8	3	2	8	1	4	8	2	3
4272	21	13	386	11	8	7	2	3139
0	0	0	0	0	0	0	0	0
2	0	0	0	0	0	0	0	20
0	0	1	56	2	1	16	11	2
0	0	0	0	10	1	28	13	0
21	623	0	0	0	0	668	8	4
0	0	0	0	0	0	4	1	2
5	0	0	4	0	0	0	1	0
0	0	0	1	0	0	0	95	1
0	0	0	0	0	0	0	0	0
0	0	0	0	0	1	12	0	0
0	0	0	0	5	0	0	0	0
0	4	2	0	0	0	4	5	0
0	0	0	0	0	0	0	0	0
0	0	2	3	1	70	0	0	0
0	2	0	0	0	2	0	0	0
0	0	0	0	0	0	0	0	0
0	2	1	0	0	1	0	0	10
0	0	0	0	0	0	0	0	0
0	0	0	0	0	0	0	0	0
0	2	0	0	0	3	0	2	0
178	2	3	4	2602	0	4	0	1
0	0	0	0	0	0	0	0	0
0	0	0	0	0	0	0	0	6
0	0	0	0	0	0	0	0	0
0	0	1	0	0	2	25	0	2

0	0	0	0	0	0	0	0	0
0	0	0	0	0	0	0	0	0
2	3	0	0	0	0	0	0	1
0	0	2407	4	3	49	0	0	0
2	1	0	0	3	2	5	2920	14
0	0	0	0	0	0	0	0	0
0	0	0	0	0	0	0	0	0
0	0	1	5	4	12	0	0	0
0	0	0	0	0	0	0	0	0
0	0	0	0	0	0	0	0	0
0	0	0	0	0	0	0	0	0
0	0	0	0	0	0	0	0	0
0	0	0	0	0	0	0	0	0
0	0	0	0	0	0	0	0	0
1	0	0	0	0	1	0	0	0
0	0	0	1	1	0	0	0	0
0	0	0	0	0	0	0	0	0
0	0	1	4	1	7	0	0	1
0	0	0	0	0	0	0	2990	0
0	0	1	6	5	0	0	0	0
0	0	0	0	0	0	0	0	0
0	0	0	0	0	0	0	0	0
0	1	0	0	0	0	0	0	1
0	2	4	1	0	0	0	2	0
0	0	0	0	0	0	0	0	0
0	0	0	0	0	1	0	0	0
1	8	0	0	0	0	0	0	2
0	0	0	0	0	0	0	6	0
0	0	0	0	0	0	0	0	2
0	0	0	0	0	0	0	0	1
10	0	1	1285	94	0	0	0	0
0	0	0	0	0	0	0	0	0
0	0	0	0	0	0	0	0	0
4	0	0	0	0	0	0	0	0
0	0	0	0	0	0	0	0	0
0	6	0	0	0	0	0	0	0
0	0	0	0	0	0	0	0	0
0	0	0	0	0	0	0	0	0
1	3	0	36	0	0	0	0	0
0	2	0	0	0	0	0	0	0
0	0	0	0	0	0	0	0	0
9	15	0	0	0	0	0	0	2757
0	0	0	0	0	0	4	0	0
0	0	0	0	0	0	0	0	0
7	0	0	0	0	15	0	0	0
0	0	0	0	0	0	0	2	0
0	0	0	0	0	0	0	0	0
0	0	0	0	0	0	0	0	0
0	0	11	20	24	40	0	0	0
0	0	0	0	0	1	0	5	0
0	0	0	0	0	0	0	0	0
0	0	0	0	0	0	0	0	0
0	1	0	0	0	0	0	0	0
0	0	0	0	0	0	0	6	0
0	0	0	0	0	0	0	0	0
0	0	0	0	0	0	0	0	0
0	0	1	4	2	0	0	0	0
0	0	0	0	0	0	0	0	0

1	0	0	0	0	0	0	0	0
0	0	0	0	0	0	0	1965	0
0	0	0	0	0	0	0	0	0
0	0	0	0	0	0	1	0	0
0	0	0	0	0	0	0	0	0
0	0	0	0	0	0	5	0	0
0	0	0	0	0	0	764	0	0
0	0	0	0	0	0	0	1	0
2	3	0	0	0	0	0	0	0
0	0	0	0	0	0	0	9	0
0	1	0	0	0	0	1	0	0
0	0	0	0	0	0	0	0	0
0	0	0	0	0	3	0	0	0
0	0	0	0	0	0	0	0	0
0	6	0	0	0	0	0	0	0
0	0	0	0	0	0	0	0	0
2	0	0	0	0	0	0	2	0
0	0	1	1210	0	0	0	0	0
0	0	0	0	0	0	0	0	0
0	0	0	0	0	0	0	0	0
0	0	0	0	0	0	0	1	0
0	0	0	0	0	0	3	0	0
0	0	0	0	1	1	0	2	0
0	2	0	0	0	0	0	0	0
0	0	0	0	0	0	0	0	0
0	0	0	0	0	0	0	0	0
4	4	0	0	0	0	0	0	5
0	0	0	0	0	0	0	2	0
0	0	0	0	0	0	0	0	0
0	0	0	0	0	0	0	0	0
0	0	0	0	0	0	0	0	0
0	0	0	0	0	0	0	0	0
0	0	0	0	0	0	0	0	0
2	2	0	0	0	0	0	0	661
0	0	0	3	1	0	5	0	0
0	0	0	15	0	12	0	0	0
0	0	0	0	0	0	0	0	0
0	0	2	0	1	0	0	0	1
0	2	0	0	0	0	1	0	0
0	0	0	0	0	0	0	0	0
40	63	0	0	0	7	0	0	23
0	0	0	0	0	0	0	0	0
0	0	0	0	0	0	0	0	0
0	2	0	0	0	0	0	0	0
0	0	0	0	0	0	0	0	0
0	0	0	0	0	0	0	0	0
0	0	0	0	0	0	0	0	0
0	0	0	0	0	0	0	0	0
0	0	0	0	0	0	0	0	0
0	0	0	0	0	0	0	0	0
0	0	0	0	0	0	0	0	0
1	0	0	0	2	0	3	1	0
0	0	0	0	0	0	0	0	0
0	0	0	8	0	0	0	0	0
0	0	0	0	0	0	0	0	0
0	0	0	0	0	0	0	0	0
0	0	0	0	0	0	0	0	0

0	0	0	0	0	0	0	0	0
0	0	0	0	0	0	0	0	0
0	0	1	0	0	0	0	0	0
0	0	0	0	0	0	0	0	0
0	0	0	0	0	0	0	0	0
1	3	4	6	4	0	0	0	1
0	0	0	0	0	1	0	0	0
0	0	0	0	0	0	0	0	0
0	0	0	0	0	0	0	0	0
0	224	1	0	0	0	0	0	0
0	0	0	0	0	0	0	0	0
0	0	0	0	0	0	0	0	0
0	0	0	0	0	0	0	0	0
0	0	0	0	0	0	0	0	0
0	0	0	0	0	0	0	0	0
0	0	2	0	0	0	19	21	0
0	0	0	0	0	0	0	0	0
0	0	0	0	0	0	0	0	0
0	0	0	0	0	0	0	0	0
0	0	0	0	0	0	0	0	0
2	0	0	0	0	0	0	0	1
0	0	0	0	0	0	0	0	0
0	0	0	0	0	0	0	0	0
0	0	1	4	1	0	0	0	0
0	0	0	1	0	0	1	0	0
0	0	0	0	0	0	0	0	0
0	0	0	0	0	0	0	0	0
0	0	0	0	0	1	0	0	0
0	0	0	0	0	0	0	0	0
0	0	1	0	0	0	0	2	0
0	0	0	0	0	0	0	0	0
0	0	0	0	0	0	0	0	0
0	0	0	0	0	0	0	0	0
0	0	0	1	0	0	1	3	0
0	0	0	0	0	0	0	0	0
0	0	0	0	0	0	0	0	0
0	0	0	0	0	0	0	0	0
0	0	0	0	0	0	0	0	0
0	0	0	0	0	0	0	0	0
0	0	0	0	0	0	0	0	0
0	0	1	0	0	0	0	0	0
0	0	0	2	0	0	0	0	1
0	0	0	0	0	1	0	0	0
0	0	0	0	0	0	0	0	0
0	0	0	0	0	0	0	0	0
0	0	0	0	0	0	0	1	0
0	0	0	0	0	0	9	0	0
0	0	0	0	0	0	0	0	0
92	0	0	0	0	0	0	0	0
1	0	0	0	0	0	0	0	1
0	0	0	36	1	0	0	0	0
0	0	0	0	0	0	1	0	0
0	0	0	0	0	0	0	0	0
0	0	0	0	0	0	0	0	0
0	0	0	0	0	0	0	0	0
0	0	0	0	0	0	0	0	0
0	0	0	0	0	0	0	0	0
0	0	0	0	0	0	0	0	0

0	0	0	0	0	0	0	2	0
0	0	0	0	0	0	0	0	0
0	0	0	0	0	0	0	0	0
0	0	0	0	0	0	1	0	0
0	0	1	0	0	0	0	0	0
0	0	0	0	0	0	0	0	0
0	0	0	0	0	0	0	0	0
0	0	0	0	0	0	0	0	0
0	0	0	0	0	0	0	0	0
0	0	0	0	0	0	0	0	0
0	0	0	0	0	0	0	0	0
0	0	0	0	0	0	0	0	0
0	0	0	0	0	0	0	0	0
0	0	0	0	0	0	0	0	0
0	0	0	0	0	0	0	0	0
0	0	0	0	0	0	0	0	0
0	0	0	0	0	0	0	0	0
0	0	0	0	0	0	0	0	0
0	0	0	0	0	0	0	0	0
0	0	0	0	0	0	0	0	0
1	1	0	0	0	0	0	0	0
0	0	0	0	0	0	0	0	0
0	0	1	0	0	0	0	0	0
0	0	0	0	0	0	0	6	0
0	0	0	0	0	0	0	0	0
0	0	0	0	0	0	0	0	0
0	0	0	0	0	0	1	0	0
1	3	0	0	0	0	0	0	2
0	0	0	0	0	0	0	0	0
1	2	0	0	0	0	0	0	0
0	0	0	0	0	0	0	0	0
0	0	0	0	0	0	0	0	0
2	3	0	0	0	0	0	0	1
0	0	0	0	0	0	0	0	0
0	0	0	0	0	0	0	0	0
0	0	0	0	0	0	0	0	0
0	0	0	0	0	0	0	0	0
0	0	0	0	0	0	0	0	0
0	0	0	0	0	0	0	0	0
0	0	0	0	0	0	0	0	0
4	3	0	0	0	0	0	0	4
0	0	0	0	0	0	0	0	0
0	0	0	0	0	0	0	0	0
0	0	0	0	0	0	0	0	0
0	0	0	0	0	0	0	0	0
0	0	0	0	0	0	0	0	0
0	0	0	1	0	0	0	0	0
0	0	0	0	0	0	0	0	0
0	0	0	0	0	0	0	0	0
0	0	0	0	0	0	0	0	0
0	0	0	0	0	0	0	0	0
0	0	0	0	0	0	0	0	0
0	0	0	0	0	0	0	0	0
0	0	0	0	0	0	0	0	0
0	0	0	0	0	0	0	0	0
0	0	0	0	0	0	0	0	0
0	0	0	0	0	0	0	0	0
0	0	0	0	0	0	0	0	0

0	1	0	0	0	0	0	0	0
0	0	0	0	0	0	0	0	0
0	0	0	1	0	0	0	0	0
0	0	0	0	0	0	0	0	0
0	0	0	0	0	0	0	0	0
0	0	0	0	0	0	0	0	0
0	0	0	0	0	0	0	0	0
0	0	0	0	0	0	0	1	0
0	0	0	0	0	0	0	0	0
0	0	0	1	1	0	0	0	0
0	0	0	2	0	0	0	0	0
0	0	0	0	0	0	0	0	0
0	0	0	0	0	0	0	0	0
88650	78729	61113	75703	92774	2232	25622	43361	91207

Ca-34	Ca-35	Ca-36	Ca-37	Ca-38	Ca-39	Ca-40	Ca-41	Ca-42
146	1485	1423	3093	9110	59	20	95834	2898
85	4484	57	82	1	9	9	63	16
3	20	29	34	33	4	2	16	18
477	0	0	0	0	5	15	7	101
22	2	1	18	6	68	48	1	0
0	0	0	0	0	0	0	0	0
19	15	1733	1815	72	64	4	2	3
1973	1	21	15	0	2806	372	1	0
1621	11	3449	21	6	3	0	9	1429
1230	70	0	1	3	0	108	22	19
13	14	105	19	1	1	0	0	2465
0	11	4561	9	0	0	0	0	5
0	0	1	1	3	0	2	0	0
0	0	0	0	1	1	67	0	0
2	0	0	0	0	11	0	0	0
0	0	0	0	0	0	0	0	0
29	2	4	53	0	1	2	193	3
2	0	0	0	0	0	0	0	0
0	0	0	0	0	0	0	0	0
0	0	0	0	0	0	0	0	0
18	5	407	13	4547	1	2	6	1
41	3621	72	60	20	0	28	1	1
0	15	2	0	0	0	0	30	0
0	3	9	2868	0	1	0	0	0
11	4	9	6	281	0	38	0	5
0	0	0	0	12	0	9	0	0
1	0	0	20	2	10	0	0	0
33	41	89	5320	3527	0	15	0	0
14	0	26	0	0	0	1	0	0
0	0	0	1	0	6	0	0	0
448	7	425	12	1182	0	9	1	1
733	29	281	1784	3	9	0	0	1
0	0	0	0	0	0	0	0	0
2	1	0	0	0	4	0	1	0
1	651	4	4	0	0	18	0	0
2	0	0	0	0	0	0	1	3
6	8	7	11	0	0	0	0	0
2315	0	0	6	0	0	0	0	0
0	6	11	3505	0	0	1	0	0
5	9	8	6	0	5	0	0	0
0	0	0	0	0	0	0	0	0
0	0	0	0	3	2	0	0	0
0	0	2	0	1	0	15	1	3656
0	0	0	0	0	224	0	0	0
0	0	0	0	0	0	0	0	0
0	3	0	0	0	0	0	0	0
5	0	0	17	0	0	0	0	0
0	0	0	0	0	5	0	0	0
1	7	13	2920	0	0	0	32	0
0	0	0	0	0	0	0	0	0
0	0	0	0	0	0	0	0	0
0	0	0	0	0	0	0	0	0
1	8	2557	3	0	0	0	143	2
0	0	0	0	0	0	1	7	7
0	0	0	0	0	0	0	0	0
0	0	0	0	0	0	0	0	0
617	3	820	6	0	0	3	0	3

0	0	0	0	0	0	0	0	0
0	0	0	0	0	0	0	0	0
1	0	0	0	0	0	0	1	2
0	683	1	5	3	0	31	0	0
3	0	0	0	1	0	2	0	0
0	0	0	12	0	1	0	0	0
0	0	0	0	0	0	0	1	1792
0	0	0	0	0	9	0	0	0
0	0	0	0	0	0	0	0	0
0	0	0	0	0	0	0	1	0
0	0	0	0	0	2	0	0	0
0	0	0	0	0	897	0	0	0
0	0	0	0	0	0	0	0	0
0	2	0	4	0	0	0	0	0
1	0	0	0	0	0	0	0	0
0	0	0	0	5041	0	0	0	0
0	0	0	0	1	0	0	0	0
380	0	0	1	0	5	2	1	1
0	0	0	0	0	0	0	0	0
0	0	0	0	4681	0	0	0	0
0	0	0	0	0	0	0	0	0
0	0	0	0	0	0	0	0	0
0	2	0	0	0	0	0	0	0
1	0	0	0	2	0	0	0	0
0	0	0	0	0	0	0	0	0
0	0	0	0	0	0	1	0	0
1208	1	0	0	0	0	0	0	0
0	0	0	0	0	0	0	0	0
0	0	0	0	0	0	0	0	0
0	0	0	0	0	0	0	0	0
0	7	5	8	0	0	0	0	0
0	0	0	0	0	0	0	0	0
0	0	0	0	0	0	0	0	0
0	1	0	0	0	0	0	0	0
0	1983	6	8	0	0	0	4	3
0	0	0	0	0	0	0	0	1
0	0	0	0	0	1	0	0	0
0	0	0	0	0	0	0	0	0
2	595	5	3	0	0	0	0	0
0	4	3	5	0	0	31	0	0
0	0	0	0	0	0	0	0	0
5	0	0	0	0	0	0	0	0
0	0	0	0	0	0	0	0	0
0	6	2	1	0	0	2	0	3
0	4	5	8	0	0	0	0	0
0	0	0	0	0	0	0	0	0
0	0	0	0	0	0	0	0	0
0	0	0	0	0	0	0	0	0
0	0	0	0	11	29	1	0	0
0	0	0	0	0	0	1	0	0
0	0	0	0	0	0	0	0	0
0	0	0	0	0	0	0	0	0
0	0	0	0	0	0	1	0	0
0	0	0	0	0	0	0	0	0
0	0	0	0	0	0	0	0	0
0	6	2052	11	0	1	0	0	0
0	0	0	0	2052	0	0	0	0
0	0	0	0	0	0	0	0	0

0	0	0	0	0	0	0	0	0
0	0	0	0	0	0	0	0	0
0	0	0	0	0	0	0	0	0
3	0	0	0	0	0	0	0	0
0	0	0	0	0	0	0	0	0
0	0	0	0	0	0	0	0	0
0	0	0	0	0	0	0	0	0
0	0	0	0	0	0	0	0	0
3	0	8	0	0	0	0	0	0
0	0	0	0	0	3	0	0	0
6	0	0	1	0	0	0	3	5
0	0	0	0	0	0	0	1	392
0	0	0	0	0	0	0	0	0
0	0	0	0	0	0	0	0	0
0	0	0	0	0	2	0	0	0
0	0	0	0	0	0	0	0	0
0	0	0	0	0	0	0	0	0
0	0	0	0	0	0	0	0	0
0	0	0	1	0	0	0	0	0
0	0	0	0	0	0	0	0	0
0	0	0	0	0	0	0	0	0
0	0	0	0	0	0	0	0	0
0	0	0	0	0	0	0	0	0
0	0	0	0	1	0	0	0	0
0	0	0	0	0	0	0	0	0
0	0	0	0	0	0	0	0	0
9	0	17	0	0	0	1	0	0
0	0	0	2	0	0	0	0	0
0	0	0	0	0	0	0	0	0
0	0	0	0	0	0	0	2	2
0	0	0	0	0	0	0	0	0
0	0	0	0	0	0	0	0	0
0	0	0	0	0	0	0	56	0
7	0	0	0	0	0	0	0	0
0	0	0	0	0	0	3	0	0
0	0	0	0	0	0	6	0	0
0	0	0	0	0	0	0	0	0
0	0	0	0	1	0	0	0	0
243	0	0	0	0	0	0	0	0
0	632	3	5	0	0	0	0	0
46	0	0	50	0	0	0	0	0
0	0	0	0	0	9	0	1	0
0	590	3	1	0	0	0	0	0
14	0	0	0	0	0	0	0	0
0	0	0	0	0	1	1	0	0
0	0	0	0	0	0	0	0	0
0	0	0	0	0	1	0	0	0
0	0	0	0	0	0	0	0	0
0	0	0	0	0	0	0	0	0
0	0	0	0	0	0	0	0	0
0	0	24	0	0	0	14	0	0
1	0	0	1	0	0	0	0	0
0	0	0	0	0	0	0	0	0
1	0	0	0	0	4	0	0	0
0	0	0	0	0	0	0	0	0
0	0	0	0	0	0	0	0	0
0	0	0	0	0	0	0	0	0

0	0	0	0	0	0	0	0	0
0	0	0	0	0	0	0	0	1
0	0	0	0	0	2	0	0	0
0	0	0	0	0	0	0	0	0
0	0	0	0	0	0	0	0	0
0	0	0	0	4	0	0	0	0
25	14	30	28	2	0	0	0	0
0	2	209	0	0	0	0	0	0
0	0	0	0	0	0	0	0	0
0	0	0	0	0	0	0	0	0
0	0	0	0	0	0	0	0	0
0	0	0	0	0	0	0	0	0
0	0	0	0	0	0	0	0	0
0	0	0	0	0	0	0	0	0
0	0	0	0	0	0	0	0	0
0	0	0	0	0	0	0	0	0
0	0	0	0	0	0	0	0	0
0	0	0	0	0	0	0	0	0
0	0	0	0	0	0	0	0	0
0	0	0	0	0	0	0	0	0
2	0	2	0	0	0	0	0	0
0	0	0	0	0	0	0	0	0
0	0	0	0	0	0	0	0	0
0	0	0	0	2	0	0	0	0
0	0	0	0	0	0	0	0	0
0	0	0	0	0	0	0	0	0
0	0	0	0	0	0	0	0	0
0	0	0	0	0	0	2	0	0
1	0	0	0	0	0	8	0	0
0	0	0	0	0	0	0	0	0
0	0	0	0	0	0	0	0	0
0	0	0	0	0	0	0	0	0
0	0	0	0	0	0	0	0	0
0	1	0	0	0	0	0	0	0
0	0	0	0	0	0	0	0	0
0	0	0	2	0	0	0	0	0
0	0	0	0	0	0	0	0	0
0	0	0	0	0	0	0	0	0
25	10	31	33	0	0	0	0	0
0	0	0	0	0	0	4	0	1
0	0	0	0	0	0	0	0	0
0	0	0	1	0	0	0	0	3
0	0	0	0	3	0	0	0	0
0	0	0	0	0	0	0	0	0
0	0	0	0	2	0	0	0	0
0	2	0	0	0	0	0	1	0
0	0	0	0	0	0	0	0	0
0	0	0	0	0	0	0	0	0
1	0	0	0	0	0	0	0	0
0	0	0	0	0	0	0	0	0
0	31	0	0	0	0	0	0	0
0	0	0	0	0	0	0	0	0
0	0	0	0	0	0	0	0	0
0	0	0	0	0	1	0	0	0
0	0	0	0	0	0	0	0	0
0	0	0	0	0	0	0	0	0
0	0	0	0	0	0	0	0	0
0	0	0	0	0	0	2	0	0

0	0	0	0	0	0	0	0	0
0	0	0	0	0	0	0	0	0
1	0	1	0	0	0	2	0	0
0	0	0	0	0	0	0	0	0
0	0	0	0	2	0	0	0	0
0	0	0	0	0	0	0	0	0
0	0	0	0	4	0	0	0	0
0	0	0	0	0	0	0	0	0
0	0	0	0	0	0	0	0	0
0	0	0	0	0	0	0	0	0
0	0	0	0	0	0	0	0	0
0	0	0	1	0	0	0	0	0
0	0	0	0	0	0	0	0	0
0	1	0	0	0	0	0	0	0
0	0	0	0	0	0	0	0	0
0	0	0	0	0	0	0	0	0
0	0	0	0	0	0	0	0	0
0	0	0	0	0	0	0	0	0
0	0	0	0	0	2	0	0	0
0	0	0	0	0	0	0	0	0
7	0	5	0	0	0	0	0	0
0	0	0	0	0	0	0	0	0
0	0	0	0	0	0	0	0	0
0	0	0	0	0	0	0	0	0
0	0	0	0	0	0	1	0	0
0	0	0	0	0	0	0	0	0
0	0	0	0	0	0	0	0	0
10	0	5	0	0	0	0	0	0
0	0	0	0	0	0	0	0	0
0	3	4	2	0	0	0	0	0
0	0	0	0	0	0	0	0	0
0	0	0	0	0	0	0	0	0
2	0	5	0	0	0	0	0	0
0	0	0	0	0	0	0	0	0
0	0	0	0	0	0	0	0	0
0	0	0	0	0	0	0	0	0
0	0	0	0	0	0	0	0	0
0	0	0	0	0	0	0	0	0
0	0	0	0	0	0	0	0	0
0	0	0	0	0	0	0	0	0
3	0	0	0	0	0	0	0	0
0	0	0	0	0	0	0	0	0
0	0	0	0	0	0	0	0	0
0	0	0	0	0	0	0	0	0
0	0	0	0	0	0	0	0	0
0	0	0	0	0	0	0	0	0
0	0	0	0	0	0	0	0	0
0	0	0	0	0	0	0	0	0
0	0	0	0	0	0	0	0	0
0	0	0	0	0	0	0	0	0
0	0	0	0	0	0	0	0	0
0	0	0	0	0	0	0	0	0
0	0	0	0	0	0	0	0	0
0	0	0	0	0	0	0	0	0
0	0	0	0	0	0	0	0	0
0	0	0	0	0	0	0	0	0
0	0	0	0	0	0	0	0	0
0	0	0	0	0	0	0	0	0

0	1	0	2	0	0	0	0	0
0	0	2	0	0	0	0	0	0
0	0	0	0	0	0	0	0	0
0	0	0	0	0	0	0	0	0
0	0	0	4	0	0	0	0	0
1	0	0	0	0	0	0	0	0
0	0	0	0	0	0	0	0	0
0	0	0	0	0	0	0	0	0
0	0	0	0	0	0	0	0	0
0	0	0	0	0	0	0	0	0
0	0	0	0	0	0	0	0	0
0	0	0	0	0	0	0	0	0
0	1	0	0	0	0	0	0	0
85157	85368	83918	73298	73646	11680	3824	1151	16082

Ca-43	Ca-44	Ca-45	N-1	N-2	N-3	N-4	N-5	N-6
1895	4641	17	739	1619	33	47	114	92
2031	1325	73	125	0	16	10	2	192
2	30	0	14	0	2	1	8	7
120	66	2	3	1	0	6	1	0
37	3	49	27	2322	44	36	1	146
0	0	0	0	1305	0	0	0	0
77	1017	76	1541	7507	290	3	0	6
0	0	11	48	0	9	11	1	1
9	3	7	22	0	0	0	0	6
24	11	1	10	0	191	4	0	0
7	2	2849	0	441	0	1	3	0
9	0	0	0	1691	0	6	0	0
1	3	0	0	76	0	2	1635	0
0	0	0	0	0	404	0	2	0
1	0	1	0	1226	1	64	3	0
0	0	0	0	2765	0	0	0	0
5	0	4	4910	6	0	0	0	0
0	0	0	0	2680	0	0	0	0
0	0	2	2	0	0	0	0	0
1	0	0	0	9	0	0	0	0
0	0	0	0	787	8	1	0	0
0	0	1	0	0	0	0	15	1
889	0	3	0	0	1	669	0	2
2	10	8	1	0	0	0	0	0
2	3	0	0	2	0	0	0	0
3	0	0	0	0	1	6	6	4
10	0	11	0	5	0	0	2	0
0	0	0	0	9	9	1	3	2
0	0	3	4	0	4	6	5951	8
0	1	0	0	0	10	0	0	1
0	5	0	5	0	2	1	1787	3
0	92	8	891	0	0	0	0	5
0	0	0	0	0	0	0	0	0
0	0	5295	0	2	4	4	0	0
2	4	2	9	61	2	2	3	0
417	20	0	11	0	1	0	5	0
0	3	0	2	0	0	0	0	0
1	0	0	0	161	1	0	0	0
12	5	0	0	0	0	1	598	3
0	0	0	0	54	1	0	2	0
0	0	0	0	6866	0	0	0	0
2	9	0	14	0	0	21	0	1616
7	10	2	0	0	259	0	4	0
0	0	0	9	0	0	0	0	0
0	0	0	0	32	0	4	0	0
8	7	7	0	0	0	93	0	0
0	0	0	0	0	0	0	0	0
0	0	0	0	0	2	0	0	0
2	15	0	0	0	0	4	0	0
0	0	0	0	15	0	0	0	0
0	0	0	0	0	0	0	0	0
2	0	0	0	0	0	0	0	0
0	0	0	0	12	0	1	3	0
0	0	1	0	0	1	0	0	0
0	13	0	0	0	0	0	0	0
0	0	3	0	0	0	0	0	0
0	0	0	0	0	0	0	1	0

0	0	1	0	0	0	0	0	0
3	0	0	0	0	0	0	0	0
1	0	0	0	21	0	0	0	0
0	0	0	0	0	0	2	1	0
3	4	0	2	0	1	37	10	59
0	0	5	0	0	0	0	0	0
0	0	0	0	0	0	0	0	0
0	0	0	0	0	0	0	0	1
0	0	0	0	4	0	0	0	0
0	0	0	0	0	0	7	0	0
0	0	0	0	0	1	0	0	1
0	0	0	0	0	1	0	0	5
0	0	1181	0	2	0	0	0	0
4	1	0	0	0	0	0	1	0
0	0	0	0	0	0	0	1	0
0	0	0	0	0	4	0	0	0
0	0	0	0	0	0	0	0	0
0	0	2	0	0	0	11	0	2
0	6	0	0	0	0	0	0	0
0	0	0	0	0	5	0	0	0
0	0	0	0	0	0	0	0	0
0	0	0	0	20	0	0	0	0
1	0	2	0	0	0	0	0	0
0	8	0	3	0	0	0	0	0
0	0	0	0	0	0	0	0	0
0	0	0	0	0	0	0	0	0
0	0	0	0	0	0	5	0	0
0	8	0	0	0	0	0	0	0
0	0	1	0	0	0	0	0	0
0	0	2	0	0	0	0	0	0
0	0	0	0	0	0	0	0	0
0	0	0	0	0	0	0	0	0
0	0	0	0	0	0	133	0	0
0	0	0	0	0	0	0	0	0
1	0	0	0	0	0	1	0	0
0	0	0	0	0	0	0	7	0
0	0	0	0	0	4	0	0	0
0	0	0	0	0	0	0	0	0
1	1	0	0	0	0	0	0	0
0	0	2	0	4	0	0	1	0
0	0	0	0	9	0	0	0	0
0	0	3	0	0	0	0	0	0
0	0	0	4	1	0	0	0	0
3	0	4	0	0	0	0	0	0
0	0	0	0	0	0	0	0	0
0	2	0	0	0	0	0	0	0
0	0	0	6	0	0	0	0	0
0	0	0	0	0	0	0	0	0
0	0	0	0	0	0	0	0	2
0	6	0	0	0	0	0	0	0
0	0	0	0	0	0	0	0	0
0	0	0	0	1	0	0	0	0
0	0	0	0	0	0	0	0	0
0	8	0	0	0	0	0	0	0
0	0	0	0	0	0	0	0	0
0	0	0	0	0	0	0	0	1
0	0	0	0	0	3	0	0	0
0	0	0	0	0	0	0	0	0

0	0	0	0	0	0	0	0	0
0	4	0	0	0	0	0	0	0
0	0	0	0	4	0	0	0	0
0	0	41	0	6	34	4	0	0
0	0	0	0	0	0	0	0	0
0	0	0	0	0	0	0	0	0
0	0	0	1	0	0	0	0	0
0	0	0	1	0	0	0	0	0
0	0	0	0	9	0	0	0	0
0	0	1	0	8	0	1	0	0
0	0	0	0	42	0	0	0	0
0	1	0	0	0	0	0	0	0
0	0	0	0	0	0	0	0	0
0	0	0	0	2	0	0	0	0
0	0	0	0	0	3	0	0	0
0	0	0	0	0	0	0	0	0
0	0	0	0	0	0	0	0	0
0	0	0	0	0	0	0	0	0
0	0	2	0	0	0	0	0	0
0	0	0	0	1	0	0	0	0
0	1	0	1	0	0	0	0	0
0	5	0	0	1	0	0	0	0
0	0	0	0	0	0	0	0	0
0	0	0	0	0	0	0	0	0
0	0	0	0	11	0	0	0	0
0	0	0	0	0	0	0	0	0
1	0	0	0	3	0	0	0	0
0	0	0	0	0	0	0	0	0
0	0	0	0	0	0	0	0	0
0	0	0	0	0	0	0	0	0
0	0	0	0	0	0	0	0	0
0	0	0	0	0	0	0	0	0
0	0	0	0	0	1	0	0	0
2	0	1	0	0	0	0	0	0
1	0	0	1	0	0	0	0	0
0	0	0	0	0	0	0	0	0
0	0	0	0	0	0	0	1	0
0	0	0	1	0	0	3	0	0
0	0	1	0	0	0	0	0	0
0	0	0	0	0	0	0	0	0
0	0	0	0	0	0	0	0	0
0	0	0	0	0	4	0	0	0
0	0	0	0	0	0	0	0	0
0	0	0	0	0	0	0	0	0
0	0	0	0	0	0	5	1	0
0	0	1	0	0	0	0	0	0
0	0	0	0	0	0	0	0	0
0	0	0	0	0	0	0	0	0
0	0	0	0	0	0	0	0	0
0	0	43	0	0	43	0	0	0
0	0	0	0	21	0	0	0	0
0	0	0	0	0	0	2	8	3
0	0	0	0	0	0	0	0	0
6	0	0	1	0	1	1	1	0
0	0	0	210	0	0	0	0	0
1	0	0	0	0	0	0	1	0
0	0	0	0	1	0	1	0	0

0	0	0	0	0	0	0	0	0
0	1	0	0	0	0	0	0	0
0	0	0	0	0	0	0	0	0
0	0	0	0	0	0	0	0	0
0	0	0	0	0	0	0	0	0
0	0	0	0	0	1	0	0	0
0	1	0	1	0	1	0	0	3
0	0	0	0	0	0	0	0	0
0	0	0	0	0	0	0	0	0
0	0	0	0	0	0	0	3	0
0	0	79	0	0	0	0	0	0
0	0	0	0	0	0	0	0	0
0	0	0	0	0	0	0	0	0
0	0	0	0	0	0	0	0	0
0	0	0	0	0	0	0	0	0
0	0	0	19	0	0	0	0	0
0	0	0	0	1	0	0	0	0
0	0	0	0	0	0	0	0	0
0	0	0	0	0	0	0	0	0
0	0	0	0	0	0	0	0	0
0	0	0	0	0	0	0	0	0
0	0	0	0	0	0	0	0	0
0	0	0	0	0	0	0	0	0
0	0	0	0	0	0	2	0	0
2	0	2	0	0	3	0	0	0
0	0	0	0	22	0	0	0	0
0	0	0	0	2	0	0	0	0
0	0	0	0	0	0	0	0	0
0	0	0	0	0	0	0	0	0
0	1	0	0	0	1	1	0	4
0	0	0	0	0	0	0	0	0
0	0	0	0	0	0	0	0	0
0	0	0	0	0	0	0	0	0
8	4	0	0	0	0	0	0	0
0	0	2	0	0	0	0	0	0
0	0	0	0	0	0	0	0	0
8	0	0	0	0	0	2	0	0
0	0	0	0	0	0	0	0	0
0	0	0	0	0	0	0	0	0
0	0	0	0	3	0	0	0	0
0	0	0	0	0	0	0	0	0
2	1	0	0	0	0	0	0	0
0	0	0	0	0	0	0	0	0
0	0	0	0	75	0	0	0	0
0	0	0	0	0	0	0	0	0
0	0	0	0	2	0	0	0	0
0	5	0	4	0	0	0	0	0
0	0	0	0	0	0	0	0	0
0	0	0	0	0	0	0	0	0
0	0	0	0	0	0	0	0	0
0	0	0	0	0	0	0	0	0
0	0	0	0	0	0	0	0	0
0	0	0	0	0	0	0	0	0
0	0	0	0	0	0	0	0	0
0	0	0	0	0	0	0	0	0
0	0	0	0	0	0	0	0	0
0	0	0	0	0	0	0	0	0
0	0	0	0	0	0	0	0	0

2	0	0	0	0	0	0	0	0
0	0	0	0	0	0	0	0	0
0	0	0	0	0	0	0	0	0
0	0	0	0	0	0	0	0	0
0	0	0	0	0	0	0	0	0
0	0	0	0	0	0	0	0	0
0	0	0	0	12	0	0	0	0
1	0	0	0	0	0	0	0	0
0	0	0	0	0	0	0	1	0
0	0	0	0	0	0	0	0	0
0	0	0	0	0	0	0	0	0
0	0	0	0	0	0	0	0	0
0	1	0	0	0	0	0	0	0
0	0	0	0	0	0	0	0	0
0	0	0	0	0	0	0	0	0
0	0	0	0	0	0	0	0	0
0	0	0	0	0	0	0	0	0
0	0	0	0	0	0	0	0	0
0	0	0	0	0	0	0	0	0
1	0	0	0	0	0	0	0	0
0	0	0	0	0	0	0	0	0
0	0	0	0	0	0	0	0	0
0	0	0	0	0	0	0	0	0
0	0	0	0	0	0	0	0	0
0	0	0	0	0	0	0	0	0
0	0	0	0	0	0	0	0	0
0	0	0	0	0	0	0	0	0
0	0	0	0	0	0	0	0	0
3	0	0	0	0	0	0	0	0
0	0	0	0	0	0	0	0	0
0	0	0	0	0	0	0	0	0
0	0	1	0	0	0	0	0	0
0	0	0	0	0	0	0	0	0
0	0	0	0	0	0	0	0	0
0	0	0	0	0	0	0	0	0
0	0	0	0	0	0	0	0	0
0	0	0	0	0	0	0	0	0
1	0	0	0	0	0	0	0	0
0	0	0	0	0	0	0	0	0
0	0	0	0	0	0	0	0	0
0	0	0	0	0	0	0	0	0
0	0	0	0	0	0	0	0	0
0	0	0	0	0	0	0	0	0
0	3	0	0	0	0	0	0	0
0	0	0	0	0	0	0	0	0
0	0	0	1	0	0	0	0	0
0	0	0	0	0	0	0	0	1
0	0	0	0	0	0	0	0	0
0	0	0	0	1	0	0	0	0
0	0	0	0	0	0	0	0	0
0	0	0	0	3	0	0	0	0
0	0	0	0	0	0	0	0	0
0	0	0	0	0	0	0	0	0
0	0	0	0	0	0	0	0	0
0	0	0	0	3	0	0	0	0
0	0	0	0	0	0	0	0	0
0	0	0	0	0	0	0	0	0
0	0	0	0	0	0	0	0	0

0	0	0	0	0	0	0	0	0
0	0	0	0	0	0	0	0	0
0	0	1	0	0	0	0	0	0
0	0	0	0	0	0	0	0	0
0	0	0	0	0	0	0	0	0
0	0	0	0	0	0	0	0	0
0	0	0	0	0	0	0	0	0
0	0	0	0	0	0	0	0	0
0	0	0	0	0	0	0	0	0
0	0	0	0	0	0	0	0	0
0	0	0	0	0	0	0	0	0
0	0	0	0	0	0	0	0	0
0	0	0	0	0	0	0	0	0
8529	23581	17769	42457	44344	73841	2750	23145	24332

N-7	N-8	N-9	N-10	N-11	N-12	N-13	N-14	N-15
4169	9	240	15	212	793	797	171	255
588	0	2	3	897	9	85	5887	3
19	0	0	0	2619	12416	0	8	0
89210	1	0	0	17	2	0	26852	0
121	186	5463	4693	4	2	2694	5268	2716
0	24061	91	70	0	0	10862	0	24210
7	2605	56	51	1433	19	2498	453	619
10	3	1	1	12	20	56	5	1
252	0	1	2	3	0	421	1	0
12	0	0	0	5	11	0	29096	0
0	67	13	18	4	17	1845	0	11
6	76	2483	2866	0	0	318	0	46
8	78	3197	2648	1	2	77	0	748
0	0	0	0	0	0	0	0	0
2	3711	749	1343	6	72	1926	0	3850
0	80	1865	2371	0	0	162	0	694
0	3	618	628	295	4	4	0	8
0	12673	130	76	0	0	2566	0	5663
0	0	1	8	0	0	1159	0	2
3	0	20	402	0	0	175	5	3
1	0	0	0	2	0	0	0	0
1	0	0	13	1	0	0	0	0
0	0	0	0	0	0	0	0	0
6	0	0	1	0	6	139	0	0
12	0	0	1	1	0	0	1	0
0	0	0	0	0	0	0	0	0
0	0	4	12	3	0	867	0	9
0	10	0	0	7	7	0	7	0
0	0	0	0	0	0	0	0	0
0	0	0	0	1	0	0	0	0
13	4	0	1	3	2861	130	1	0
40	0	0	0	0	0	0	0	0
0	0	0	0	4	0	0	0	0
0	0	0	0	0	6	0	7	0
0	2	3	27	4	3	101	390	1
1284	0	0	0	2	5	0	0	0
3	0	434	1	14	15	66	0	0
4	310	1	0	0	0	0	0	1863
0	0	0	1	0	1	136	0	0
0	337	22	9	1	0	621	2299	42
0	443	15	20	0	0	553	0	2021
0	0	1	0	0	2	0	0	0
0	0	0	0	0	0	0	14	0
0	0	0	0	1165	4	0	0	0
0	2570	2	1004	0	0	436	0	0
6	0	0	0	3	16	0	0	0
0	0	0	0	0	0	83	0	1
0	0	0	0	0	0	0	0	0
1	2	5	10	929	19	7	0	5
0	13	964	787	0	0	240	0	26
0	0	0	0	0	0	0	0	0
3	1	1	0	0	0	91	8	1190
0	0	0	110	0	0	215	0	0
0	0	0	0	0	0	0	1	0
17	0	0	0	0	0	0	0	0
0	0	0	0	0	0	0	0	0
2	0	0	0	0	0	0	2991	0

0	0	0	0	0	0	0	0	0
0	0	0	0	0	0	0	0	0
3	10	0	0	0	0	0	0	0
0	0	0	0	2	0	0	1	0
0	0	1	645	1	27	4	2	0
0	0	0	0	0	0	0	0	0
0	0	0	0	0	0	0	0	0
0	0	0	0	0	0	0	0	8
0	2	239	0	3	3	0	0	0
0	4	1	465	0	4	6	0	841
0	0	0	0	0	0	0	0	0
0	0	0	0	0	0	0	0	0
0	0	3	1	0	0	0	0	561
27	0	0	1	0	0	0	0	0
8	0	0	0	1	0	0	0	0
0	0	0	0	0	0	0	0	0
0	0	2	6	0	0	482	0	0
1	0	0	0	0	3	0	0	0
4	0	0	0	0	0	0	0	0
0	0	0	0	0	0	0	0	0
0	0	0	0	0	0	0	0	0
0	1474	15	2	0	0	552	0	114
0	0	0	0	0	0	0	0	0
0	0	0	0	0	0	0	0	0
0	0	0	0	0	0	0	0	0
0	0	0	0	0	0	0	0	0
1	0	0	0	0	0	0	0	0
0	0	0	1	0	0	137	0	1
0	0	0	0	0	0	0	0	0
0	0	0	0	0	0	0	0	0
0	0	0	0	0	0	0	0	0
0	0	0	0	0	0	0	0	0
0	0	0	0	0	0	0	0	0
0	0	0	0	0	0	0	0	0
1	0	2	2	0	0	1	3	0
0	0	0	0	0	3	86	1	0
0	0	0	0	0	0	0	0	0
0	0	0	0	0	0	0	0	0
0	0	0	0	0	0	0	0	0
3	331	1	0	2	1	45	0	1
0	0	1	4	0	0	191	0	0
0	0	0	0	0	0	0	0	0
0	12	1	0	0	0	0	0	4
0	0	0	0	0	0	0	0	0
0	0	0	0	0	0	0	0	0
1	0	0	0	4	1	0	0	0
0	0	0	0	12	0	0	0	0
0	0	0	0	0	0	0	0	0
0	0	0	0	0	0	0	0	0
0	0	0	0	0	0	0	0	0
0	0	0	0	0	0	0	0	0
0	0	0	3	0	0	0	0	0
0	0	0	0	0	0	0	0	0
4	1	0	1	0	0	0	0	0
0	0	0	0	0	0	0	0	0
0	0	0	0	0	0	0	0	0
0	0	0	0	0	0	0	0	0
0	11	627	0	0	0	0	0	0

0	0	0	0	3	4	0	0	0
0	0	0	0	0	0	0	0	0
0	0	0	1	0	0	276	0	0
3	101	7	7	1	12	248	0	0
0	0	0	0	0	0	0	0	2
0	0	0	0	0	0	0	0	0
0	0	0	0	0	0	0	0	0
0	0	0	16	0	0	130	0	4
0	886	0	0	0	0	0	0	0
0	440	0	4	0	3	1	0	0
0	30	13	14	6	5	222	0	0
0	0	0	0	0	0	0	0	0
1	0	0	0	0	0	0	0	0
0	0	0	0	0	0	1	0	0
0	0	0	0	0	0	0	0	0
0	1	7	3	0	0	1	0	11
1	0	0	0	0	0	0	0	0
0	0	0	0	0	0	0	0	0
0	0	0	0	0	0	0	0	0
0	0	0	0	0	708	0	0	2
0	0	0	0	0	0	0	0	0
0	1	1	1	0	0	252	0	599
0	0	0	0	0	0	0	0	0
0	0	0	0	0	0	0	1	0
0	254	6	7	0	0	542	0	0
0	0	0	0	0	0	0	0	0
0	495	0	0	2	0	0	0	0
0	0	0	0	0	0	0	0	0
0	0	0	0	1	3	0	0	0
0	0	0	0	0	0	0	0	3
0	0	1	6	0	0	695	0	0
0	0	0	0	0	0	0	0	0
0	3	14	5	0	0	464	0	0
2	0	0	0	0	0	0	0	0
0	0	0	0	0	0	0	0	0
0	0	0	0	0	0	0	0	0
0	0	0	0	0	0	0	0	0
0	0	0	2	6	1	464	0	0
1	0	0	0	0	0	0	0	0
0	0	0	0	0	0	0	0	0
0	0	0	0	0	0	0	0	0
0	0	0	0	0	1	0	0	0
0	0	0	0	0	0	0	0	0
0	0	0	0	0	0	0	0	0
0	1	4	0	0	3	0	0	3
0	0	0	0	0	0	0	0	0
181	0	3	2	0	0	281	0	1
0	0	1	0	0	0	0	0	0
0	0	0	0	0	0	0	0	0
0	0	0	0	0	0	0	0	0
0	26	0	0	0	0	0	0	20
0	0	0	1	0	11	0	0	0
0	0	0	5	0	0	391	0	10
1	0	0	0	0	0	0	11	0
0	0	0	0	0	0	0	0	0
0	0	0	3	0	0	339	0	0
0	216	50	0	0	0	2	0	0

0	1	0	2	0	0	0	0	0
0	0	0	1	0	0	205	0	0
0	0	0	0	0	0	0	0	0
0	0	0	0	0	0	0	0	0
0	0	0	0	2	0	0	0	0
0	0	0	0	0	0	0	0	0
0	0	0	0	1	0	0	1	0
0	0	0	0	0	0	0	0	0
0	0	0	0	0	0	0	0	0
0	0	0	0	0	0	0	0	0
0	0	0	0	0	0	0	0	0
0	0	0	0	0	0	0	0	223
0	0	0	2	0	1	243	0	0
0	0	0	58	0	0	0	0	0
0	0	0	4	0	0	226	0	0
0	0	0	0	9	0	0	0	0
0	0	0	2	0	0	217	0	0
0	0	0	0	0	0	0	0	0
0	0	0	7	0	0	199	0	0
0	0	0	2	0	0	199	0	0
0	0	0	3	0	0	193	0	0
0	1	0	2	0	0	189	0	0
0	0	0	0	0	0	190	0	0
1	0	0	0	0	0	0	0	0
0	0	0	0	0	0	0	0	0
0	25	19	16	0	0	0	0	0
0	154	0	0	0	0	0	0	0
0	0	0	0	0	0	0	0	0
1	0	0	0	0	0	0	0	0
0	0	0	0	0	1	0	0	0
0	0	0	0	0	0	0	0	0
0	0	0	0	0	0	0	0	0
0	0	0	0	0	0	0	0	0
0	2	0	0	2	0	0	0	0
0	0	0	0	0	0	0	0	0
0	0	0	0	0	0	0	0	0
0	0	0	0	0	0	0	0	0
0	0	0	0	0	0	0	0	0
0	0	0	0	0	0	0	0	0
0	0	0	0	0	0	2	3	0
0	0	0	0	0	0	0	0	0
0	0	0	0	0	0	0	0	0
0	0	0	0	0	0	0	0	0
0	0	0	0	0	0	0	0	0
0	0	0	0	0	0	0	0	0
0	4	11	2	0	0	0	1	0
0	0	0	0	0	0	0	0	0
0	0	0	0	0	0	0	0	0
0	0	0	0	0	0	0	0	0
0	0	0	0	0	0	0	0	0
0	0	0	0	0	0	0	0	0
0	0	5	4	0	0	0	0	0
0	0	0	0	0	0	72	0	0
0	0	0	0	0	0	0	0	0
0	0	0	0	0	0	0	0	0
0	0	0	0	0	0	15	0	0
0	20	0	1	0	0	19	0	4
2	0	0	0	0	0	0	0	0

0	0	0	0	0	0	0	0	0
0	0	0	0	0	0	0	0	0
1	0	0	0	0	0	0	0	0
0	0	0	0	0	0	0	0	0
0	0	0	0	0	0	0	0	0
0	0	0	0	0	0	0	0	0
0	6	1	6	0	0	0	0	0
0	0	1	0	0	0	0	0	0
0	0	0	0	0	0	0	0	0
0	0	0	0	0	0	0	0	0
0	0	15	0	0	0	0	0	0
0	0	0	0	0	0	0	0	0
0	0	0	0	0	0	0	0	0
0	0	0	0	0	0	0	0	0
0	0	0	0	0	0	0	0	0
0	0	0	0	0	0	0	0	0
0	8	4	0	0	0	0	0	0
0	0	0	0	1	4	0	0	0
0	0	0	0	0	0	0	0	0
0	0	0	0	0	0	0	0	0
0	0	0	0	0	0	0	0	0
0	0	0	0	0	0	17	0	0
0	0	0	0	0	0	0	0	0
0	0	0	0	0	0	0	0	0
0	0	0	0	0	0	0	1	0
0	0	0	0	0	0	0	0	0
1	0	0	0	0	0	0	0	0
0	0	0	0	0	0	0	0	0
0	0	0	0	0	0	0	0	0
0	0	0	0	0	0	0	0	0
0	0	0	0	0	0	0	0	0
0	0	0	0	0	0	0	0	0
0	0	0	0	0	0	0	0	0
0	0	1	0	0	0	0	0	4
0	0	1	2	0	0	0	0	0
0	0	0	0	0	0	0	0	0
0	0	3	0	0	0	0	0	1
0	0	0	0	0	0	0	0	0
0	0	0	0	0	0	0	0	0
0	0	0	0	0	0	1	0	0
0	0	0	0	0	0	0	0	0
0	0	0	0	0	0	0	0	0
0	0	1	0	0	0	1	0	0
0	0	0	0	0	0	0	0	0
0	0	3	0	0	0	0	0	0
0	0	0	0	0	3	0	0	0
0	0	0	0	0	0	0	0	0
0	0	0	0	0	0	0	0	0
0	0	0	0	0	0	0	0	0
0	0	0	0	0	0	0	0	0
0	1	0	0	0	0	0	0	0
0	0	2	0	0	0	0	0	0
0	0	0	0	0	0	0	0	2
0	0	0	0	0	0	0	0	0
0	0	0	0	0	0	0	0	0
0	0	1	0	0	0	0	0	0
0	0	0	1	0	0	0	0	0
0	0	0	0	0	0	0	0	0

0	0	0	0	0	0	0	0	0
0	0	0	0	0	0	0	0	0
0	0	0	0	0	0	0	0	0
0	0	0	0	0	0	0	0	0
0	0	0	0	0	0	0	0	0
0	0	0	0	0	0	0	0	0
0	0	0	0	0	0	0	0	0
0	0	0	0	0	0	0	0	0
0	0	0	0	0	0	0	0	0
0	0	0	0	0	0	0	0	0
0	0	0	0	0	0	0	0	0
0	0	0	0	0	0	0	0	0
0	0	0	0	0	0	0	0	0
10665	39460	15019	21222	8444	49525	52017	36468	30533

N-16	N-17	N-18	N-19	N-20	N-21	N-22	N-23	N-24
314	25	152	685	11	4	418	5330	99
4	18	0	8	1	0	1733	33	1
0	2337	1	0	0	0	18	9721	0
49	0	0	0	8	10	37	0	0
129	21	23	1461	6890	4554	41	0	3965
8	0	0	10993	649	12	0	0	13734
130	62	165	64	215	468	895	15	574
1	12	0	2	0	1	54	0	129
0	2	0	0	0	0	8515	16	2
0	0	1	0	0	0	7	5	0
557	0	0	2293	553	149	0	135	219
2221	0	1	21	3358	3146	0	16	1219
24	0	1	1	2395	1196	0	0	1359
0	718	1120	0	0	0	0	0	0
1577	4	21	66	3207	3025	1	0	944
73	0	0	4	1529	3702	0	0	1746
4	0	3	5	1458	665	1	4	635
29	0	0	2878	109	160	0	0	4972
4	0	0	9	1	0	1	0	7
2	0	0	3	1	2	0	0	1
5	2	0	0	6	120	0	6	0
0	8	0	0	0	0	0	29	1
0	0	1	0	0	0	0	0	0
2	3	0	0	0	5	3	8	4
238	0	0	1	0	2	0	1008	0
0	101	20	0	0	0	0	0	0
13	0	0	1481	4	185	12	0	5
786	0	0	5	0	53	0	37	0
0	0	1	0	0	0	12	0	0
0	6	2	0	0	0	4	0	0
0	0	11	0	0	0	7	724	0
0	0	1	0	0	0	764	43	0
0	0	0	0	0	0	0	0	0
0	0	0	0	1	0	0	0	0
267	0	0	162	134	4	46	2	40
0	8	0	0	0	0	1832	0	0
0	4	0	0	0	0	1068	4687	0
8	0	0	6580	969	1	3	0	0
0	0	0	0	0	0	12	12	0
1753	0	0	25	17	1182	0	4	71
51	0	0	38	136	65	0	0	62
4	0	1	2	2	0	530	0	0
0	1	0	0	0	52	0	0	0
0	373	0	0	0	0	4	0	0
1396	0	0	8	13	6	0	0	10
0	2	0	0	0	0	0	0	0
0	0	0	0	218	0	1	0	0
0	1205	0	0	0	0	0	0	0
5	2	11	1275	0	0	1	5	563
809	0	0	8	1	881	0	0	310
0	0	0	0	0	0	0	0	0
1	0	2	7	0	16	1	0	0
0	0	94	0	0	0	0	7	1
0	0	0	0	0	0	0	0	0
0	0	0	0	0	0	0	0	0
0	0	0	0	0	0	0	0	0
0	0	7	0	0	0	0	3	0

0	0	0	0	0	0	0	0	0
0	0	0	0	0	0	0	0	0
0	0	0	0	990	0	0	0	0
0	7	4	0	0	0	0	27	1
0	2	9	0	0	0	2	2	1
0	0	0	0	0	0	0	0	0
0	0	0	0	0	0	1	0	0
0	0	0	0	5	14	5	0	0
1549	0	0	10	4	7	0	0	0
4	0	0	7	3	18	0	0	12
0	562	0	0	0	0	0	0	0
0	0	0	0	0	0	0	0	0
0	0	0	5	0	3	0	0	0
0	0	0	0	0	0	277	2	0
0	2	0	0	0	0	7	0	0
0	0	0	0	0	0	0	0	0
1	3	0	0	0	2	0	0	2
0	0	0	0	0	0	50	0	0
0	0	0	0	0	0	0	2	0
0	0	0	0	0	0	0	0	0
0	0	0	0	0	0	0	0	0
4	0	0	5	24	20	0	0	18
0	0	0	0	0	0	1	2	2
0	0	0	0	0	0	0	0	0
0	0	0	0	0	0	0	0	0
0	0	0	0	0	0	0	0	0
0	0	0	0	0	0	0	0	0
1	0	0	0	0	70	9	0	1
0	0	0	0	0	0	0	0	0
0	0	0	0	0	0	0	0	0
0	0	0	0	0	0	0	2051	0
0	0	0	0	0	0	0	0	0
0	0	1	0	0	0	0	0	0
0	0	0	0	0	0	0	1	0
2	0	4	0	0	0	0	10	340
2	0	5	2	0	77	0	0	0
0	0	0	0	0	0	0	0	0
0	0	79	0	0	0	0	0	0
0	0	0	0	0	0	0	2	0
7	0	0	2	0	1	0	3	47
1413	0	0	4	2	9	0	0	0
0	0	0	0	0	0	0	0	0
25	0	0	25	4	10	3	0	6
0	0	0	0	0	0	0	0	0
0	0	0	0	0	0	0	2227	0
0	2	0	0	0	0	0	0	0
0	0	0	0	0	0	0	0	0
0	0	0	0	0	0	0	0	0
0	0	0	0	0	0	4	0	0
0	0	0	0	0	0	7	0	0
0	0	0	0	0	0	0	0	0
0	0	0	0	0	1	0	0	0
0	0	0	0	0	0	0	0	0
0	0	0	0	0	1	0	0	0
0	0	0	0	0	0	0	0	0
0	0	0	0	0	0	0	6	0
0	0	0	0	0	0	0	0	0
0	0	0	0	0	0	0	0	9

0	2	0	0	0	0	0	0	0
0	0	0	0	0	0	0	1	0
815	0	0	5	0	4	0	0	1
7	1	0	3	10	0	0	0	0
8	0	0	1231	0	0	0	0	0
0	0	0	0	0	0	0	0	0
0	0	0	0	0	0	2	0	0
22	0	1	446	3	0	0	0	0
0	0	0	0	5	0	0	0	5
2	0	0	4	3	1	0	0	312
2	0	0	1	16	7	0	0	0
0	0	0	0	0	0	0	0	0
0	0	0	0	0	0	2	0	0
734	0	0	10	0	3	0	0	1
0	0	0	0	0	0	0	0	0
0	0	0	0	5	213	0	0	0
0	0	0	0	0	0	0	0	0
0	0	0	0	0	0	0	0	0
0	0	0	0	0	0	0	0	0
0	0	0	0	0	66	0	0	1
0	0	0	0	0	0	0	0	0
0	0	0	4	1	3	3	0	1
0	0	0	0	0	0	2	0	0
0	0	0	0	0	0	0	10	0
13	0	0	2	7	0	0	0	5
0	0	0	0	0	0	0	0	0
0	0	0	0	2	0	0	0	1
0	0	0	0	0	0	1	0	0
0	0	0	0	0	0	0	0	0
3	0	0	500	0	2	0	0	0
0	0	0	1	0	2	0	0	5
0	0	0	0	0	0	0	0	0
11	0	0	13	9	1	0	0	4
0	0	0	0	0	0	0	0	0
0	3	0	0	0	0	258	0	0
0	0	0	0	0	0	0	0	0
0	1	0	0	0	0	0	0	0
0	0	0	0	1	0	4	0	4
0	0	0	0	0	0	0	0	0
0	0	0	0	0	0	0	4	0
0	0	0	0	0	0	0	0	0
0	2	0	0	0	0	0	0	0
0	0	0	0	0	0	0	3	0
0	0	0	0	0	0	0	0	0
0	0	12	0	7	5	0	0	0
0	0	7	0	0	0	0	0	0
0	0	0	2	0	0	0	0	0
3	0	0	399	0	0	0	0	1
0	0	0	0	0	0	0	0	0
0	1	0	0	0	0	0	0	0
0	0	0	10	15	0	0	0	0
0	0	2	0	0	0	2	0	1
0	0	0	0	0	0	0	0	1
0	6	0	0	0	0	0	0	0
0	0	0	0	0	0	1	0	0
0	0	0	1	0	0	0	0	1
0	0	0	0	0	0	0	0	0

0	0	0	0	0	0	0	0	241
0	0	0	0	0	0	0	0	0
0	1	1	0	0	49	0	0	0
0	0	0	0	0	0	0	0	0
0	0	0	0	0	0	0	0	0
0	0	1	0	0	0	0	0	0
0	0	0	0	0	0	0	19	0
0	0	0	0	0	0	0	0	0
0	0	0	0	0	0	0	0	0
0	0	0	0	0	0	0	0	0
0	0	0	0	0	0	0	0	0
0	0	0	0	0	0	0	0	0
0	0	0	0	0	0	0	0	0
0	14	0	0	0	0	0	0	0
0	0	0	0	0	0	0	0	1
0	0	0	0	0	0	33	0	0
0	0	0	0	0	0	0	0	2
0	0	0	0	0	0	0	0	0
0	0	0	0	0	0	0	0	0
0	0	0	0	0	0	0	0	1
0	0	0	0	0	0	0	0	0
0	0	0	0	0	0	0	0	1
0	0	0	0	0	0	0	0	1
0	0	0	0	0	0	0	0	0
0	4	0	0	0	0	0	0	0
0	0	0	0	18	0	0	0	0
0	0	0	0	3	0	0	0	1
0	0	0	0	0	44	0	0	0
0	0	0	0	0	0	0	0	0
0	1	0	0	0	0	0	0	0
87	0	0	3	0	0	0	0	2
0	0	0	0	0	0	0	0	0
0	0	0	0	0	0	0	0	0
0	0	0	0	1	0	0	0	0
0	0	0	0	0	0	0	0	0
0	0	0	0	0	0	0	0	0
0	0	0	0	0	0	0	0	0
0	0	0	0	0	0	0	0	0
0	0	0	0	0	0	0	20	0
0	0	0	0	0	0	0	0	1
0	0	0	0	0	0	0	0	0
0	1	0	0	0	0	0	0	0
0	0	0	0	0	0	0	0	0
0	0	0	0	0	0	0	0	0
0	0	0	0	0	0	0	0	0
0	0	0	4	3	0	0	1	0
0	0	0	0	0	0	3	0	0
6	0	0	5	4	0	1	0	1
0	0	0	0	0	0	0	0	0
0	0	0	0	0	0	0	0	0
0	0	0	0	0	0	0	1	0
5	0	0	2	0	13	3	0	0
0	0	0	0	0	0	0	0	0
0	0	1	0	0	0	0	0	0
0	0	0	0	0	0	0	0	0
11	0	0	11	6	1	0	0	1
0	0	0	0	1	0	0	0	0
0	0	0	0	0	0	0	0	0

0	1	0	0	0	0	0	0	0
0	0	0	0	0	0	0	0	0
0	0	0	0	0	0	0	0	0
0	0	0	0	0	0	0	0	0
0	0	0	0	0	0	0	0	0
0	0	0	0	0	0	0	0	0
0	0	0	0	4	0	0	0	0
0	1	0	0	0	0	0	0	0
0	0	0	0	0	0	0	0	0
0	0	0	0	0	0	0	0	0
0	0	0	0	0	0	0	0	0
0	0	0	0	0	0	0	0	0
0	0	0	0	0	0	1	0	0
0	0	0	0	0	0	0	0	0
0	0	0	0	0	0	0	0	0
0	0	0	0	0	0	0	0	0
0	0	0	0	0	0	0	0	1
0	0	0	0	0	0	0	0	0
0	0	0	0	0	0	0	0	0
0	0	0	0	0	0	0	0	0
0	0	0	0	0	0	0	0	0
0	0	0	0	3	0	0	0	0
0	0	0	0	0	0	0	0	0
0	0	0	0	0	0	0	18	0
0	0	0	0	0	0	0	0	0
0	0	0	0	0	0	0	0	0
0	0	0	0	0	0	1	0	0
0	0	0	0	0	0	0	0	0
0	0	0	0	0	0	0	0	0
0	0	0	0	0	0	0	1	0
0	0	0	0	0	0	0	0	0
0	0	0	0	0	0	0	0	0
0	0	0	0	0	0	0	0	0
0	0	0	1	0	0	0	0	0
0	0	0	0	0	0	0	0	1
0	0	0	1	0	0	0	0	0
0	0	0	1	0	0	0	0	0
0	0	0	0	0	0	0	0	0
0	0	0	0	0	0	0	0	0
0	0	0	0	0	0	0	0	8
0	0	0	0	0	0	0	0	0
0	0	0	0	0	0	0	0	0
0	0	0	0	0	1	0	0	1
0	0	0	0	0	0	0	0	0
0	0	0	0	0	0	0	0	0
0	0	0	0	0	0	0	0	0
0	0	0	0	0	0	0	0	0
0	0	0	0	0	0	0	0	0
0	0	0	0	1	0	0	0	0
0	0	0	0	0	0	0	0	0
0	0	0	1	0	0	0	0	0
0	0	0	0	0	0	0	0	0
0	0	0	1	0	0	1	0	0
0	0	0	0	0	0	0	0	2
1	0	0	0	0	0	0	0	0
0	0	0	0	0	0	0	0	0
1	0	0	1	0	0	0	0	0
3	0	0	0	0	0	0	0	0

0	0	0	0	0	0	0	1	0
0	0	0	0	0	0	0	0	0
0	0	0	0	0	0	0	0	0
0	0	0	0	0	0	0	0	0
0	0	0	0	0	0	0	0	0
0	0	0	0	0	0	0	0	0
0	0	0	0	0	0	0	0	0
0	0	0	0	0	0	0	0	0
0	0	0	0	0	0	0	0	0
0	0	0	0	0	0	0	0	0
0	0	0	0	0	0	0	0	0
0	0	0	0	0	0	0	0	0
0	0	0	0	0	0	0	0	0
40391	4414	2565	47585	23554	23006	28795	75889	27257

N-25	N-26	N-27	N-28	N-29	N-30	N-31	N-32	N-33
25	169	1	127	846	184	0	0	0
3	4	1	171	2434	15	16	3797	2619
2173	0	0	1	6749	5	1	0	0
3	0	0	3	0	0	4	1288	0
4	0	3310	93	13	0	2	1	2279
0	0	1488	0	0	0	0	0	0
11	2	4472	66	8	7	0	0	0
0	4706	5	52	13	6	0	2	12
7	0	0	13	1596	19	0	0	0
2	1156	0	0	0	1	0	1	51
2	0	16	475	7	14	2664	171	3368
2	0	1237	0	1	0	0	0	0
1	0	3550	1	0	1	1	999	524
0	0	0	0	0	0	0	0	1670
0	0	1018	6	96	1	0	0	0
0	0	2529	0	0	0	0	0	0
0	1804	3	85	0	0	0	0	0
0	0	127	0	0	0	0	0	0
0	0	0	33	0	0	0	0	19
0	0	0	0	0	0	17366	1705	1
397	0	0	0	0	0	3077	14	11981
22	2	0	33	131	38	0	0	0
0	0	0	23	1	0	0	3	0
0	0	0	1694	1	6	5	2	0
3	0	0	2	4	1	5739	16	412
8	0	0	0	0	0	6845	3383	1841
0	0	4	6943	0	1	0	1	5
3	0	0	32	58	35	0	0	0
0	0	0	0	0	1	3	6	0
0	0	0	0	0	0	0	0	0
2	919	0	1	8	0	1	763	935
2	0	0	7	54	20	0	0	0
0	0	0	0	0	0	0	0	0
0	9	0	25	4	17	0	0	0
1	501	1	2	0	602	8	27	390
0	0	0	1	0	0	0	0	0
0	0	0	0	368	0	0	0	0
0	0	3	1	0	0	5	2	4
0	0	0	8	2105	2	0	0	0
1	0	466	1	0	3	0	0	0
0	0	23	0	0	0	0	0	0
0	0	0	0	0	0	7	19	458
0	0	3	0	0	0	0	0	0
0	0	0	0	5	2	0	0	231
0	3383	9	0	0	0	0	0	0
0	0	0	4	0	0	0	0	0
0	0	60	10	0	0	2	6378	0
3	0	0	0	0	0	0	0	0
0	0	4	11	22	1	0	0	0
0	0	938	0	0	0	0	0	0
0	0	0	0	0	0	8	6645	0
0	0	3	0	0	0	0	0	0
0	0	0	1	18	5	0	0	0
0	0	0	0	0	0	0	0	5920
0	0	0	0	0	0	0	0	0
0	0	0	7	0	0	0	0	0
0	0	0	1	0	0	0	0	2311

0	0	0	3	0	0	0	0	0
0	0	0	0	0	0	0	0	0
0	0	0	0	0	0	0	0	0
13	279	0	0	213	0	0	0	6
7	1	0	0	30	3	0	0	0
0	0	0	2285	0	0	0	0	0
0	0	0	0	0	0	0	0	0
0	0	11	0	0	0	13	5112	0
0	0	0	0	0	0	0	1	0
0	0	1264	0	5	1	0	0	0
0	0	0	0	0	0	0	0	0
0	0	0	0	0	0	0	0	0
0	0	5	15	0	4	0	0	0
0	0	0	0	0	0	11	8	8
0	0	0	2	2	0	0	0	0
0	0	0	0	0	0	0	0	0
0	0	1	0	0	0	8	3884	0
0	0	0	798	0	0	0	0	0
0	0	0	0	0	0	0	0	0
0	0	0	0	0	0	0	0	0
0	0	0	0	0	0	23	1	0
0	0	27	0	0	0	0	0	0
0	0	0	3	489	1	1502	7	0
0	0	0	0	0	0	0	0	0
0	0	0	0	0	0	23	0	2
228	0	0	0	0	0	0	0	0
0	1905	1	0	2	0	0	0	0
0	0	0	0	0	0	0	0	0
0	0	0	0	2	624	0	0	0
0	0	0	0	2	510	0	3	1
0	0	0	0	8	0	0	0	0
0	0	0	0	0	0	0	0	3007
0	0	0	0	0	0	0	0	0
0	536	0	0	997	1	0	0	21
0	1	2	0	1	0	0	0	0
0	0	0	0	0	0	0	0	0
0	0	0	0	0	0	0	0	1
0	0	0	0	0	0	945	4	0
0	0	0	0	0	0	0	0	0
2	0	7	6	0	0	4	133	42
0	0	0	0	0	0	0	0	0
0	0	0	0	0	13	0	0	0
0	0	1047	0	0	0	0	0	0
0	0	0	3	1	0	0	0	0
0	0	0	0	6	0	0	0	0
0	0	0	0	0	69	0	0	0
0	0	0	0	0	0	612	0	7
0	0	0	0	0	0	10	0	5
0	0	0	0	0	0	1	786	0
0	0	0	0	0	0	0	0	0
0	0	0	0	0	0	6	0	4
0	0	0	0	0	0	1700	8	0
0	0	0	0	0	0	1723	12	0
0	0	0	0	0	1	0	0	0
0	0	0	0	0	0	4	1905	0
0	0	0	0	0	0	0	0	0
0	0	0	0	0	0	0	0	0
0	0	6	0	0	0	0	0	0

1	0	0	0	0	0	0	0	647
0	0	0	0	0	0	0	0	0
0	0	0	0	0	0	0	0	0
0	0	0	1	11	3	0	0	0
0	0	0	0	0	0	0	0	0
0	0	0	0	0	0	0	0	0
0	0	0	0	0	0	0	0	0
0	0	0	0	0	0	0	0	47
0	0	5	0	0	0	0	0	395
0	0	3	6	7	0	0	0	0
1	0	0	0	5	3	0	0	0
0	0	0	0	0	0	0	0	0
0	0	0	0	0	0	0	0	0
0	0	0	0	0	0	0	0	0
0	11	0	1	1	0	0	0	0
0	0	307	0	0	0	0	0	0
0	0	0	0	0	0	0	0	0
0	0	0	0	0	0	0	0	0
0	0	0	434	0	0	0	0	0
0	0	0	0	0	0	0	0	0
0	0	0	0	0	0	0	0	0
0	0	5	0	0	0	0	0	0
0	0	0	0	0	0	0	0	0
0	0	0	0	0	1	0	0	0
0	0	1	0	0	0	0	0	0
0	0	0	0	0	0	3	822	0
0	0	1	0	0	0	0	0	0
0	0	0	0	0	0	0	0	0
0	0	0	0	0	0	0	0	0
0	0	63	0	0	0	0	0	0
0	0	0	0	0	0	0	0	0
0	0	0	0	0	0	0	3	1
0	0	5	0	0	0	0	0	1
0	0	0	0	0	6	0	0	0
1	0	0	0	0	0	0	0	0
9	0	0	0	0	0	0	0	0
0	0	0	0	0	0	9	625	0
0	0	0	0	1	0	0	0	0
0	0	0	2	0	0	0	0	0
0	0	0	0	0	0	0	0	0
0	0	0	34	0	0	0	0	0
0	0	1	0	0	0	0	0	0
0	0	0	0	0	0	0	0	0
0	0	0	0	0	0	0	0	508
0	0	2	3	4	3	0	0	0
0	0	0	0	0	0	0	0	0
0	0	0	0	0	0	0	0	0
0	0	0	0	0	0	0	0	0
0	0	0	0	0	1	0	408	0
0	0	0	1	0	2	0	0	0
0	0	0	0	0	0	0	0	0
5	0	0	1	11	0	0	0	0
0	0	0	0	0	0	0	0	0
3	1	0	2	0	0	0	0	0
0	0	0	0	0	0	0	0	0
0	0	0	0	0	0	0	0	0
0	0	0	0	0	1	0	0	0

0	0	0	0	0	0	0	0	0
0	0	0	0	0	0	0	0	0
0	0	0	0	0	0	6	10	1
0	0	0	0	0	0	1	0	0
0	0	0	0	0	0	0	0	0
0	0	0	0	0	0	0	0	0
0	0	0	1	1	1	0	0	0
0	0	0	0	0	0	1	0	0
0	0	0	0	0	0	0	0	0
0	0	0	0	0	0	0	0	0
0	0	0	0	0	0	0	0	0
0	0	0	0	0	0	0	0	0
0	0	0	1	0	0	0	0	0
0	0	0	0	0	0	0	0	0
0	0	0	0	0	0	0	0	0
0	0	0	0	0	0	0	0	0
0	0	0	0	0	0	0	0	0
0	0	0	0	0	0	0	0	226
0	0	0	0	3	0	0	0	0
0	0	0	0	0	0	0	0	0
0	0	0	0	0	0	0	0	0
0	0	0	0	0	0	0	0	0
0	0	0	0	0	0	0	0	0
0	0	0	0	0	0	0	0	133
0	0	0	2	0	0	0	0	0
0	0	0	0	0	0	0	0	0
0	0	1	0	0	0	0	0	0
0	0	1	0	0	0	0	0	0
0	0	0	0	0	0	0	0	0
0	0	0	0	0	0	0	0	76
0	0	0	0	0	0	0	0	0
0	0	0	0	0	0	0	0	0
0	0	0	0	0	0	0	0	0
0	0	0	0	0	0	0	0	0
0	0	0	0	0	0	0	0	0
0	0	0	0	4	0	0	0	0
0	0	0	0	0	0	0	0	0
0	0	0	0	0	0	0	0	109
0	0	0	0	0	0	0	0	0
0	0	0	0	0	0	0	0	0
0	0	0	0	0	0	0	0	0
0	0	0	0	0	0	0	0	0
0	0	0	0	0	0	0	0	0
0	0	0	0	0	0	0	0	0
0	0	0	0	0	1	0	0	0
1	3	0	0	1	0	0	0	0
0	0	0	0	0	0	0	0	0
0	0	0	0	0	0	0	0	0
0	0	0	0	0	0	0	0	0
5	0	0	0	0	0	0	0	0
0	0	0	0	0	0	0	0	0
0	0	0	0	0	0	0	0	0
0	0	0	0	0	0	0	0	0
0	0	0	4	0	5	0	0	0
0	0	0	0	0	0	0	0	5
0	0	0	0	0	0	0	0	0
1	0	0	0	0	0	0	0	0
0	0	0	0	0	0	0	0	0

0	0	0	0	0	0	0	0	0
0	0	0	0	0	10	0	0	0
0	0	0	0	0	0	0	0	0
0	0	0	0	0	0	0	0	0
0	0	0	0	0	0	0	0	0
0	0	0	0	0	0	0	0	0
0	0	0	0	0	0	0	0	0
0	0	0	0	0	0	0	0	0
0	0	0	0	0	7	0	0	0
0	0	0	0	1	0	0	0	0
0	0	0	0	0	0	0	0	0
0	0	0	0	0	0	0	0	0
1	0	0	0	0	0	0	0	0
0	0	0	0	0	5	0	0	0
0	0	0	0	0	0	0	0	0
0	0	0	0	0	0	0	0	0
0	0	2	0	0	0	0	0	0
0	0	0	0	0	0	0	0	0
0	0	0	5	0	0	0	0	0
0	0	0	0	0	0	0	0	0
0	0	0	0	0	0	0	0	0
0	0	0	0	0	0	0	0	0
1	0	0	0	0	1	0	0	0
0	0	0	0	0	0	0	0	0
0	0	0	0	0	0	0	0	0
0	0	0	0	0	0	0	0	0
0	0	0	0	0	0	0	0	0
0	0	0	0	0	0	0	0	0
0	0	0	0	0	0	0	0	0
0	0	0	0	0	0	0	0	0
1	0	0	0	0	0	0	0	0
0	0	0	2	0	0	0	0	0
0	0	0	0	0	0	0	0	0
0	0	0	0	0	0	0	0	0
0	0	3	0	0	0	0	0	0
0	0	0	0	0	0	0	0	0
0	0	0	0	0	0	0	0	0
0	0	0	0	0	0	0	0	0
0	0	0	0	0	0	0	0	0
0	0	0	0	0	0	0	0	0
0	0	0	0	0	0	0	0	0
0	0	0	0	0	0	0	0	0
0	0	0	0	0	0	0	0	0
0	0	0	0	0	0	0	0	0
0	0	0	0	0	0	0	0	0
0	0	0	0	0	0	0	0	0
0	0	0	0	0	0	0	0	0
0	0	0	0	0	0	0	0	0
0	0	0	0	0	0	0	0	0
0	0	0	0	0	0	1	3	0
0	0	0	0	0	0	0	0	0
0	0	0	0	0	0	0	0	0
0	0	0	0	0	0	0	0	0
0	0	1	0	0	0	0	0	0
0	0	0	0	0	0	0	0	0
0	0	1	0	0	0	0	0	0
0	0	0	0	0	0	0	0	0
0	0	0	0	0	0	0	0	0

0	0	0	0	0	0	0	0	0
0	0	0	1	0	0	0	0	0
0	0	0	0	0	0	0	0	0
0	1	0	0	0	0	0	0	0
0	0	0	0	0	0	0	0	0
0	0	0	0	1	0	0	0	0
0	0	0	0	1	0	0	0	0
0	0	0	0	0	0	0	0	0
0	0	0	0	0	0	0	0	0
0	0	0	0	0	0	0	0	0
0	0	0	0	0	0	0	0	0
0	0	0	0	0	0	0	0	0
0	0	0	0	0	0	0	0	0
2763	64600	18080	21567	24910	14507	41939	53986	48486

N-34	N-35	N-36	N-37	N-38	N-39	N-40	N-41	N-42
0	0	29	1	23095	2494	88	33955	15762
1034	4979	0	1	38	67	16	1972	12557
0	0	0	0	28	0	2	15249	18
0	6	24	0	1096	0	10	1513	319
1071	105	2	6364	3912	35	5116	5	6
0	0	0	157	0	0	0	0	0
0	0	82	123	24	486	68	6	109
21	638	1	99	0	8	9169	1	0
0	0	0	0	1	6	0	6926	3304
13	522	135	0	4	0	1	6	40
1580	2893	0	1191	0	5	0	2	0
0	0	0	2070	0	0	0	3774	5
281	9	1	77	0	0	0	13	3619
3	4	0	0	0	0	4	0	0
0	0	0	94	0	18	0	5	1
0	0	0	3727	0	0	0	0	0
0	0	0	2698	0	3	3	0	0
0	0	0	776	0	1	0	0	0
22	22	0	4	0	5679	0	0	0
8730	17	2	2	0	0	0	0	0
67	747	5	1	0	0	5	0	0
0	0	0	0	0	356	8	3	0
0	0	0	0	0	12	0	1	1
0	0	0	1	7	4	0	21	12
1331	13	372	0	2	0	7	5492	1374
8834	488	0	0	0	0	0	0	0
10	10	0	6	2	6	0	0	0
0	0	0	0	0	0	12	1	0
0	0	0	0	0	0	1	0	0
0	0	0	0	1	0	7589	1	0
1	44	12	2	0	3	0	6	1614
0	0	0	0	2	15	0	12	2528
0	0	1218	0	0	0	0	0	0
0	0	1	0	0	7	0	0	0
259	2869	0	2	3	0	0	19	1
0	0	2	0	9	1	1	12	12
0	0	0	0	8	11	0	6	2
7	4	0	7	0	0	0	1	0
0	0	0	1	7	4	0	30	0
0	0	0	1832	0	0	1	0	0
0	0	0	72	0	0	0	0	0
2	1	4	0	0	2	0	0	12
0	0	0	1	0	0	1	0	3
1	1	2	0	0	0	1	2	0
0	0	0	3	0	0	0	0	0
0	0	0	0	0	2	0	5	2440
3	1648	0	0	0	1	0	0	0
0	0	0	0	1	0	4	0	0
0	0	3	5	1	0	1	0	8
0	0	0	31	0	0	0	0	0
3	1294	0	0	0	0	0	0	0
0	0	0	9	0	0	0	3	2
0	0	0	2	0	0	10	1	0
18	8	0	0	1	1	0	0	0
0	0	0	0	0	0	0	18	8036
0	0	0	0	0	1388	0	0	0
3	4	0	0	0	3	0	0	46

0	0	0	0	0	1260	0	0	0
0	0	0	0	0	5	0	0	0
0	0	0	0	2	0	0	0	0
5	5	0	0	0	0	0	0	0
0	0	2	0	15	0	0	19	14
0	0	0	0	0	3	0	0	0
0	0	0	0	0	0	0	0	0
1	15	0	6	0	0	0	0	0
0	0	0	0	0	0	0	0	0
0	0	0	16	0	0	0	1	0
0	0	0	0	0	0	0	0	0
0	0	0	0	0	0	1	0	0
0	0	0	4	0	4	0	0	0
886	1	2	0	0	2	1	0	0
0	0	0	0	0	0	0	8	7
0	0	3	0	0	0	0	0	0
0	18	1	2	0	0	0	0	0
0	0	0	0	0	142	0	0	0
0	0	0	0	4	0	0	6	0
0	0	6	0	0	0	0	0	0
3893	0	0	0	0	0	0	0	0
0	0	0	1076	0	0	0	0	0
5	232	0	0	0	0	0	0	0
0	0	0	0	3197	0	0	0	0
3609	6	0	0	0	0	0	0	0
0	0	0	0	0	0	0	0	0
0	0	0	0	0	0	0	0	0
0	0	0	2	4	0	0	0	0
0	0	0	0	0	0	0	0	0
0	217	0	0	0	0	0	0	0
0	0	0	0	0	0	0	0	0
6	8	0	0	0	0	0	0	0
0	0	0	0	0	0	0	0	0
0	0	0	0	0	0	0	0	0
0	0	0	0	0	0	0	0	0
0	0	0	0	0	1	0	0	0
1	0	0	0	0	0	904	1	0
6	0	0	0	0	0	0	0	0
0	0	0	0	0	0	0	0	0
0	3	0	2	0	1	0	417	6
0	0	0	3	0	0	0	0	0
0	0	0	0	0	0	0	0	0
0	0	0	6	0	0	0	0	0
0	0	0	0	0	3	0	0	0
0	0	0	0	0	0	0	0	0
0	0	0	0	0	0	0	0	0
1325	11	4	0	0	0	0	0	0
2116	2	0	0	0	0	0	0	0
0	8	0	0	0	0	0	0	0
0	0	0	0	1	0	0	0	0
1905	6	0	0	0	0	0	0	0
15	0	0	0	0	0	0	0	0
9	2	0	0	0	0	0	0	0
0	0	0	0	4	0	0	0	0
0	6	0	0	0	0	0	0	0
0	0	0	0	1	0	0	0	0
4	2	0	0	0	0	0	0	0
0	0	0	0	0	0	0	0	0

1	0	0	0	0	0	0	1	0
0	0	0	0	2	0	0	2	0
0	0	0	2	0	0	0	0	0
0	0	0	6	0	16	31	0	0
0	0	0	0	0	0	0	0	0
0	0	0	0	0	0	0	0	0
0	0	0	0	0	0	0	0	0
0	178	0	1	0	0	0	0	0
0	0	0	0	0	0	0	0	0
0	0	0	2	0	2	0	0	0
0	0	0	6	0	0	0	0	0
0	0	0	0	1	0	0	0	0
0	0	0	0	0	0	0	5	2
0	0	0	0	0	0	0	0	0
0	0	0	0	0	0	391	0	0
0	0	0	8	0	0	0	0	0
0	0	0	0	0	0	0	1320	1
0	0	0	0	0	0	0	0	0
0	0	0	0	0	0	0	0	0
0	0	0	0	0	0	0	0	0
0	0	0	0	900	0	0	0	0
0	0	0	7	0	0	0	0	8
0	0	0	0	242	0	0	0	0
0	0	0	0	0	0	0	0	0
0	0	0	2	0	0	0	0	0
0	5	0	0	0	0	0	0	0
0	0	0	0	0	0	0	0	0
0	0	0	0	2	0	0	0	0
0	0	0	0	0	0	0	0	0
0	0	0	0	0	0	0	0	0
0	0	0	7	0	0	0	0	0
0	288	0	0	0	0	0	0	0
3	16	0	4	0	0	0	0	0
0	0	0	0	0	0	0	0	0
0	0	0	0	0	0	1	0	0
0	0	0	0	0	0	0	0	0
0	0	0	0	0	0	0	0	0
0	0	1	5	0	0	0	0	0
0	0	0	0	0	1	0	1	0
0	0	0	0	0	0	0	0	0
0	0	0	0	0	0	0	0	0
0	0	0	0	0	0	1	0	0
0	0	0	0	0	0	0	0	0
0	2	0	0	0	0	0	0	0
0	0	0	1	0	3	0	0	0
0	0	0	0	0	6	0	0	0
0	0	0	2	0	0	0	1	1
0	0	0	0	0	0	0	0	0
0	0	0	0	0	0	0	0	0
0	0	0	0	0	4	0	0	0
0	0	0	0	0	0	0	0	0
0	0	0	0	0	3	1	2	2
0	0	0	6	0	0	0	0	0
0	0	1	0	0	3	1	0	1
0	0	0	0	3	0	0	0	0
0	0	1	5	0	0	0	0	0
0	0	0	0	0	0	0	0	0

0	0	0	0	0	0	0	0	0
0	0	0	1	0	0	0	0	0
3	2	0	0	0	0	1	0	0
341	3	0	0	0	0	0	0	0
0	0	0	0	0	0	0	0	0
0	0	0	0	0	0	0	0	0
0	0	3	0	0	4	2	0	1
104	3	0	0	0	0	0	0	0
0	113	0	0	0	0	0	0	0
0	0	0	0	0	0	0	0	0
0	0	0	0	0	1	0	0	0
0	0	0	1	0	0	0	0	0
0	0	0	0	1	0	0	0	0
0	0	0	0	0	0	0	0	0
0	0	0	2	0	0	0	0	0
0	0	0	0	0	0	0	21	0
0	0	0	3	0	0	0	0	0
0	0	0	0	0	0	0	0	0
0	0	0	0	0	0	0	0	0
0	0	0	2	0	0	0	0	0
0	0	0	0	0	0	0	0	0
0	0	0	1	0	0	0	0	0
0	0	0	1	0	0	0	0	0
0	0	0	0	0	0	0	0	0
0	0	0	0	0	0	2	0	0
0	0	0	0	0	0	0	0	0
0	0	0	0	0	0	0	0	0
0	0	0	0	0	0	0	0	0
0	0	0	0	0	0	0	0	0
0	0	0	0	7	0	0	0	0
0	0	0	0	0	0	0	0	0
0	0	0	0	0	0	0	0	0
0	0	0	0	0	0	0	0	0
0	0	1	0	7	0	0	0	0
0	0	0	0	0	2	0	0	0
0	0	0	0	0	0	0	0	0
0	0	0	0	0	0	0	0	0
1	0	0	0	0	0	0	0	0
0	0	0	0	0	0	0	0	0
0	0	0	0	0	0	0	0	0
0	0	0	0	0	0	0	0	0
0	1	0	0	0	0	0	0	0
0	0	0	0	0	0	0	0	0
0	0	0	0	0	0	0	0	0
0	0	5	0	0	0	0	0	0
0	0	0	0	2	0	0	0	0
0	0	1	0	0	0	0	0	9
0	0	0	0	0	0	0	0	0
0	0	0	0	0	0	0	0	0
0	0	0	0	0	0	0	0	2
0	0	0	0	0	0	0	0	0
0	0	0	0	0	0	0	0	0
0	0	0	0	0	0	0	0	0
0	0	0	0	0	0	0	3	0
24	15	0	0	0	0	0	0	0
0	0	0	0	0	0	0	0	0
0	0	0	0	0	0	0	0	0
0	0	0	0	0	0	0	5	3

0	0	0	0	0	0	0	3	0
3	2	0	0	0	0	0	0	0
0	0	1	0	0	0	0	1	0
0	0	0	0	0	1	0	0	0
0	0	0	0	0	0	0	0	0
0	20	0	0	0	0	0	0	0
0	0	0	0	0	0	0	0	0
0	0	0	0	0	0	0	0	0
0	0	0	0	0	0	0	0	1
0	0	0	0	0	0	0	0	0
0	0	0	0	0	0	0	0	0
0	0	0	0	0	0	0	0	0
0	0	0	0	0	0	1	0	0
0	0	0	0	0	0	0	0	0
0	0	0	0	0	0	0	0	0
0	0	0	0	0	0	0	0	0
0	0	0	0	0	0	0	0	0
0	0	0	0	0	0	0	0	0
0	0	0	0	0	0	0	0	0
0	0	1	0	0	0	0	0	0
0	0	0	0	0	0	0	2	2
0	0	0	0	0	0	0	0	0
0	0	0	0	0	0	0	0	0
0	0	0	0	1	0	0	0	0
0	0	0	0	0	0	0	0	0
0	0	0	0	0	0	0	0	0
0	0	0	0	0	0	0	3	1
0	0	0	0	0	0	0	0	0
0	0	0	0	0	0	0	0	0
0	0	0	0	0	0	0	0	1
0	0	0	0	1	0	0	0	0
0	0	0	0	0	1	0	0	0
0	0	0	0	0	0	0	0	0
0	0	0	0	0	0	0	0	0
0	0	0	0	0	0	0	0	0
0	0	0	0	0	0	0	0	0
0	0	0	1	0	0	0	0	0
0	0	0	0	0	1	0	0	0
0	0	0	0	0	2	0	0	0
0	0	0	0	0	0	0	0	0
0	0	0	0	0	0	0	0	0
9	0	0	0	0	0	0	0	0
0	0	0	0	0	0	0	0	0
0	0	0	0	1	0	0	0	0
0	0	0	0	0	0	0	0	0
0	0	0	0	0	0	0	0	0
0	0	0	0	0	0	0	0	0
0	0	0	0	0	0	0	0	0
0	0	0	2	0	0	0	0	0
1	0	0	0	0	0	0	0	0
0	0	0	0	0	0	0	0	0
0	0	0	0	0	0	0	0	0
0	0	0	0	0	0	0	0	0
0	0	0	0	0	0	0	0	0
0	0	0	0	0	0	0	0	0
0	0	0	0	0	0	0	0	0
0	0	0	0	0	0	0	0	0
0	0	0	0	0	0	0	0	0

0	0	0	0	0	0	0	0	0
0	0	0	0	0	0	0	0	0
0	0	0	0	0	0	0	0	0
0	0	0	0	0	0	0	0	0
0	0	0	0	0	0	0	0	0
0	0	0	0	0	0	0	0	0
0	0	0	0	0	0	0	0	0
0	0	0	0	0	0	0	0	0
0	0	0	0	0	0	0	0	0
0	0	0	0	0	0	0	0	0
0	0	0	0	0	0	0	0	0
0	0	0	0	0	0	0	0	0
0	0	0	0	0	0	0	0	0
48448	17640	3558	39669	47049	2397	6850	30876	30978

N-43	N-44	N-45
10278	35548	18115
13955	2784	7662
15	27	0
9318	806	0
242	4	1115
0	0	0
808	7032	1616
0	0	21
7669	10	3
9	3	1
0	2	13
2	0	0
7	0	0
0	0	0
595	4	3
0	0	0
0	0	2219
0	0	0
0	0	14
0	0	0
288	0	1
0	0	18
5	0	4279
6334	4	8
26	0	1
0	0	0
0	5	13
2	3	12
3	0	0
0	0	2
199	3	134
626	2	1
0	0	0
0	0	10
1	2734	5
7	5291	38
239	6	0
0	0	1
0	2960	4
0	0	0
0	0	0
0	1	0
0	0	0
0	0	0
0	0	0
8	0	2126
6	0	0
0	0	0
0	0	3
0	0	0
0	0	0
6	2	0
0	0	0
0	0	0
9	0	0
0	0	2
0	0	0

0	0	3
0	0	0
0	0	0
0	0	1
2	8	5
0	0	9
0	0	0
0	0	0
0	0	0
0	0	0
0	0	0
0	0	0
0	0	0
0	332	0
3	5	0
0	0	0
0	0	0
1	0	4
0	5	0
0	0	0
0	0	0
0	0	0
0	0	682
155	2	0
0	0	0
0	0	0
0	0	0
0	3677	0
0	0	0
0	0	0
0	0	0
0	0	7
0	0	0
0	0	0
0	0	5
2	0	0
1	0	0
0	0	1
0	0	1
9	0	313
0	0	0
0	0	0
0	0	0
0	0	2374
0	0	0
0	2	0
0	0	0
0	0	0
0	0	0
0	2394	0
0	0	0
0	0	0
0	0	0
0	6	0
0	0	0
0	0	0
0	0	0
0	0	0

0	0	0
0	3	0
0	0	0
0	0	0
0	0	0
1	0	0
0	0	0
0	49	0
0	0	0
193	0	0
0	0	0
0	0	0
1	0	0
0	0	0
0	0	0
0	0	0
2	0	0
0	0	0
0	0	0
0	1	1
0	2	0
0	0	0
0	823	0
1	0	1
0	0	0
0	0	0
0	0	0
0	932	0
0	0	0
0	0	0
0	0	0
0	0	0
0	0	0
0	0	0
0	0	0
0	0	0
0	0	0
0	0	0
260	0	2
0	0	0
0	0	0
0	0	0
0	0	3
0	0	0
0	0	0
0	0	0
0	0	0
0	0	0
0	0	0
0	0	3
0	0	0
0	0	0
0	0	0
1	0	8
0	0	0
0	0	0
0	0	0

0	0	0
0	0	0
0	0	0
0	0	0
0	0	0
0	0	0
0	0	1
0	0	0
0	0	0
0	0	0
0	0	0
0	0	0
0	0	3
0	0	0
0	0	0
0	0	0
0	0	0
0	0	0
0	0	0
0	0	0
0	0	0
0	0	0
0	0	0
0	0	0
0	0	5
0	0	0
0	0	0
1	0	0
1	0	0
0	4	0
0	0	0
0	0	0
0	0	0
0	2	0
0	0	0
0	0	0
0	0	0
0	0	0
0	0	0
0	0	0
0	0	0
0	0	1
0	0	0
0	0	0
0	0	0
0	0	0
0	0	0
0	0	0
0	0	0
0	0	0
0	0	1
0	0	0
0	0	0
0	0	0
0	0	1
0	0	0
0	0	0
1	0	0

0	0	0
0	0	0
0	0	0
0	0	0
0	0	0
0	0	0
0	0	0
0	0	2
0	0	0
0	0	0
0	0	0
0	0	0
0	0	0
0	0	0
20	0	0
0	0	0
0	0	0
0	0	0
0	0	0
0	0	1
2	0	0
0	0	0
0	0	0
0	0	0
0	0	0
15	0	0
3	0	0
0	0	0
0	0	0
0	0	0
0	0	0
0	0	1
0	0	0
0	0	0
0	0	0
0	0	0
0	0	0
0	0	0
0	0	1
0	0	0
0	0	0
0	0	0
0	0	0
0	0	0
0	0	0
0	0	0
0	0	0
0	0	0
0	0	0
0	0	0
0	0	0
0	0	0
0	0	0
0	0	0
0	0	0
0	0	0
0	0	0
0	0	0

0	0	0
0	0	0
0	0	0
0	0	0
0	0	0
0	0	0
0	0	0
0	0	0
0	0	0
0	0	0
0	0	0
0	0	0
0	1	0
14437	44076	53616

Supplementary Table 8. The values of alpha diversity indexes.

Group	Sobs	Shannon	Simpson	Chao	Ace	Goods_coverage
**GC**	116.9111	2.597738	0.650631	149.2669	151.1759	0.998435
**Control**	143.2889	3.764508	0.841553	179.8205	176.1494	0.998582

Supplementary Table 9. Guild's fungal function classification prediction		
Guild	GC	Control
Undefined Saprotroph	47.157542	40.164516
Animal Pathogen-Endophyte-Lichen Parasite-Plant Pathogen-Soil Saprotroph-Wood Saprotroph	7.938322	2.486869
Animal Pathogen-Dung Saprotroph-Endophyte-Epiphyte-Plant Saprotroph-Wood Saprotroph	1.871642	4.823560
Plant Pathogen	1.815502	3.591951
Animal Pathogen-Endophyte-Plant Pathogen-Wood Saprotroph	3.073987	1.147318
Animal Pathogen	1.059553	3.129244
Fungal Parasite-Plant Pathogen-Plant Saprotroph	0.991824	2.882824
Endophyte-Plant Pathogen	0.944687	2.796316
Fungal Parasite-Undefined Saprotroph	0.806376	2.060411
Animal Pathogen-Plant Pathogen-Undefined Saprotroph	1.568940	0.964582
Ectomycorrhizal-Fungal Parasite	0.367029	0.543816
Plant Saprotroph-Wood Saprotroph	0.408202	0.500789
Animal Pathogen-Clavicipitaceous Endophyte-Fungal Parasite	0.034298	0.753560
Animal Pathogen-Undefined Saprotroph	0.223653	0.532358
Plant Pathogen-Undefined Saprotroph	0.173900	0.576887
Endophyte-Plant Pathogen-Wood Saprotroph	0.532571	0.112258
Animal Pathogen-Endophyte-Epiphyte-Plant Pathogen	0.084342	0.539867
Dung Saprotroph-Undefined Saprotroph	0.110238	0.456638
Animal Pathogen-Soil Saprotroph	0.068787	0.448036
Ericoid Mycorrhizal	0.447129	0.030198
Animal Pathogen-Endophyte-Plant Pathogen-Undefined Saprotroph	0.113880	0.297780
Endophyte-Litter Saprotroph-Soil Saprotroph-Undefined Saprotroph	0.025218	0.362407
Ectomycorrhizal	0.137973	0.240547
Fungal Parasite-Wood Saprotroph	0.369187	0.000242
Wood Saprotroph	0.179791	0.177322
Endophyte-Lichen Parasite-Plant Pathogen-Undefined Saprotroph	0.146122	0.209860
Undefined Saprotroph-Wood Saprotroph	0.101171	0.242291
Animal Pathogen-Dung Saprotroph-Endophyte-Lichen Parasite-Plant Pathogen-Undefined Saprotroph	0.297462	0.045718
Animal Pathogen-Endophyte-Fungal Parasite-Plant Pathogen-Wood Saprotroph	0.091649	0.241422
Dung Saprotroph	0.004456	0.319953
Dung Saprotroph-Wood Saprotroph	0.094887	0.220780
Fungal Parasite	0.042667	0.272773
Endophyte	0.004816	0.308051
Plant Pathogen-Plant Saprotroph	0.037367	0.233469

Dung Saprotroph-Endophyte-Litter Saprotroph-Undefined Saprotroph	0.000000	0.240960
Endophyte-Fungal Parasite-Plant Pathogen	0.073222	0.149400
Animal Endosymbiont-Animal Pathogen-Endophyte-Plant Pathogen-Undefined Saprotroph	0.094816	0.124331
Ectomycorrhizal-Undefined Saprotroph	0.010751	0.173000
Animal Pathogen-Wood Saprotroph	0.095147	0.019336
Plant Saprotroph	0.018264	0.094009
Fungal Parasite-Litter Saprotroph	0.051036	0.058064
Clavicipitaceous Endophyte-Plant Pathogen	0.000000	0.099047
Lichenized	0.002304	0.094622
Epiphyte-Plant Pathogen	0.000842	0.091720
Plant Pathogen-Wood Saprotroph	0.000187	0.084578
Fungal Parasite-Protistan Parasite	0.042427	0.042042
Soil Saprotroph	0.052373	0.027889
Endophyte-Plant Pathogen-Plant Saprotroph	0.075529	0.002242
Dung Saprotroph-Soil Saprotroph	0.024607	0.048756
Epiphyte	0.000496	0.058591
Ectomycorrhizal-Orchid Mycorrhizal-Root Associated Biotroph	0.018351	0.040218
Dung Saprotroph-Plant Saprotroph	0.000318	0.054453
Animal Pathogen-Endophyte-Lichen Parasite-Plant Pathogen-Wood Saprotroph	0.000044	0.050929
Plant Pathogen-Undefined Parasite-Undefined Saprotroph	0.000000	0.042702
Dung Saprotroph-Endophyte-Undefined Saprotroph	0.000831	0.030091
Dung Saprotroph-Soil Saprotroph-Undefined Saprotroph	0.030447	0.000313
Lichen Parasite	0.000000	0.029898
Undefined Saprotroph-Undefined Biotroph	0.015040	0.010680
Animal Pathogen-Plant Pathogen-Soil Saprotroph-Undefined Saprotroph	0.000000	0.022967
Endophyte-Lichen Parasite-Undefined Saprotroph	0.001276	0.015884
Animal Pathogen-Fungal Parasite-Undefined Saprotroph	0.000000	0.015491
Leaf Saprotroph-Plant Pathogen-Undefined Saprotroph-Wood Saprotroph	0.000042	0.012771
Endophyte-Litter Saprotroph-Wood Saprotroph	0.000000	0.009456
Ectomycorrhizal-Fungal Parasite-Plant Pathogen-Wood Saprotroph	0.000269	0.009176
Ectomycorrhizal-Fungal Parasite-Plant Saprotroph-Wood Saprotroph	0.008691	0.000218
Dung Saprotroph-Ectomycorrhizal-Litter Saprotroph-Undefined Saprotroph	0.006404	0.000307
Dung Saprotroph-Plant Saprotroph-Wood Saprotroph	0.000491	0.005820
Arbuscular Mycorrhizal	0.000318	0.005084

Ectomycorrhizal-Endophyte-Ericoid Mycorrhizal-Litter Saprotroph-Orchid Mycorrhizal	0	0.004811
Dung Saprotroph-Ectomycorrhizal	0.001427	0.003162
Orchid Mycorrhizal	0.001769	0.002318
Bryophyte Parasite-Litter Saprotroph-Wood Saprotroph	0.00018	0.002731
Endomycorrhizal-Plant Pathogen-Undefined Saprotroph	0.000311	0.002207
Epiphyte-Undefined Saprotroph	0.00224	0.000236
Animal Pathogen-Dung Saprotroph-Endophyte-Plant Saprotroph-Soil Saprotroph-Wood Saprotroph	0.001469	0.00066
Endophyte-Soil Saprotroph	0.001264	0.000153
Ectomycorrhizal-Fungal Parasite-Soil Saprotroph-Undefined Saprotroph	0.001018	0
Animal Pathogen-Endophyte-Plant Saprotroph-Soil Saprotroph	0	0.000542
Endophyte-Plant Pathogen-Undefined Saprotroph	2.00E-05	0.000504
Lichenized-Undefined Saprotroph	0.000316	6.22E-05
Lichen Parasite-Wood Saprotroph	9.33E-05	0
Endophyte-Leaf Saprotroph-Plant Pathogen	2.22E-05	5.11E-05
Unassigned	28.0402	26.50293

Supplementary Table 10. Trophic's fungal function classification prediction		
Trophic	GC	Control
Pathogen-Saprotroph-Symbiotroph	0.091649	0.241422
Pathotroph	2.960149	7.108611
Pathotroph-Saprotroph	3.237184	5.530389
Pathotroph-Saprotroph-Symbiotroph	14.93753	11.70505
Pathotroph-Symbiotroph	1.951549	5.003922
Saprotroph	48.55197	42.31377
Saprotroph-Symbiotroph	0.06376	0.8445
Symbiotroph	0.166027	0.749431
Unassigned	28.0402	26.50293
